# Supplementary material for: Dose- and genotype-dependent cardiac arrhythmia and sudden death in rats following microdystrophin gene therapy
Source: Mol Ther. 2025 Oct 24;34(1):140–60. doi: 10.1016/j.ymthe.2025.10.041 (PMC12925808; doi:10.1016/j.ymthe.2025.10.041)
Supplement: Document S1. Figures S1–S10, Tables S1–S4, and supplemental materials and methods [file mmc1.pdf]

YMTHE, Volume *34*

Supplemental Information

Dose- and genotype-dependent cardiac arrhythmia and sudden death in rats following microdystrophin gene therapy

Caroline Le Guiner, Gilles Toumaniantz, Thibaut Larcher, Sylvie Marchand, Laurine Buscara, Grégory Cedrone, Cladice Varela Moreira, Amandine Lancelot, Christophe Georger, Aude Lafoux, Célia Couzinié, David Augereau, Corinne Jounier, Agnès Hivonnait, Estelle Creoff, Stéphanie Blaie, Corinne Huchet, Oumeya Adjali, Nathalie Daniele, Gérald Perret, and Serge Braun

SUPPLEMENTAL MATERIALS AND METHODS

Vector genome quantification

For the animals which survived to 6 months p.i., vector quantification was based on transgene (hMD1)-specific qPCR analysis of genomic DNA(gDNA) from tissue samples (biceps femoris muscle, heart [left ventricle, right ventricle, left atrium, right atrium, interventricular septum], diaphragm, liver [central lobe], lung, kidney, adrenal glands) obtained just after sacrifice under conditions that minimized cross-contamination and avoided qPCR inhibition. In particular, the samples were directly snap-frozen in liquid nitrogen and then stored at ≤-65°C. gDNA was extracted from snap frozen tissues using TissueLyserII (Qiagen) and NucleoSpin® Tissue kits (Macherey Nagel) according to the manufacturer's instructions. Simplex qPCR analyses were conducted on a QuantStudio7 light cycler (Applied Biosystems) using 600 ng of gDNA. Reactions were performed in triplicate in a final volume of 30 µL containing template DNA, qPCR Rox mix (Abgene), 200 nmol/L of each primer, and 100 nmol/L of Taqman probe. Vector copy numbers were determined using the following primer/probe combination, designed to amplify a region of the hMD1 transgene: Forward primer : 5'- CCAACAAAGTGCCCTACTACATC-3', Reverse primer : 5'- GGTTGTGCTGGTCCAGGGCGT- 3', Probe : 5'- CCGAGCTGTACCAGAGCCTGGCC- 3'. For each sample, cycle threshold (Ct) values were compared with those obtained from a linearized standard curve derived from various dilutions of hMD1 expression plasmid. The Lower Limit of Quantification (LLOQ) of the assay was determined as 50 vector genomes per µg of gDNA.

Immunological analyses

For the animals which survived to 6 months p.i. and injected with Vehicle or with 4.2×10^{14} vg/kg of GNT0004, anti-hMD1 IgG was analyzed in sera obtained before injection and at 6 months p.i. by Western-blot. Briefly, hMD1-positive protein extracts acquired from HEK293 cells transfected with a pCMV-hMD1 plasmid were subjected to precast polyacrylamide gel electrophoresis using the NuPAGE Large Protein Blotting Kit (3%–8% Tris acetate precast polyacrylamide gel; Invitrogen) and subsequently blotted. After overnight saturation, the membranes were incubated with rat sera collected before and after treatment at a dilution of 1/500. After hybridization with a peroxidase-conjugated anti-rat IgG antibody, proteins were visualized by enhanced chemiluminescence. For each Western-blot membrane, an anti-Dystrophin antibody (MANEX1011C) was used as a positive control. To validate the specificity of the assay (anti-hMD1 IgG detection in rat sera), the results from sera tested on non-transfected (hMD1-negative) cell extracts were compared to those obtained for transfected (hMD1-positive) cell extracts.

On the same animals, anti-hMD1 and anti-AAV8 cellular responses were analyzed using IFNγ ELISpot assays (Mabtech) on splenocytes isolated from fresh spleen samples obtained at sacrifice. T-cell responses were evaluated with an IFN-γ ELISpot assay using the following antigens: overlapping peptide libraries covering the entire sequences of the hMDl protein (length, 15 mers; overlap, 10 amino acids; total peptides, 238) or of the AAV8 capsid protein (length, 15 mers; overlap, 10 amino acids; total peptides, 146). The hMDl peptide library was divided into five peptide pools, and the AAV8 peptide library into three peptide pools. For each specimen, the negative control consisted of unstimulated cells (Medium) and the positive control was cells stimulated with mitogenic Concanavalin A (ConA). Each stimulation antigen and control condition were tested in triplicate. IFN-γ secretion was measured as the number of spot-forming cells (SFC)

per 10^6 cells. The threshold of positivity for IFN- γ secretion was defined for each sample as three-fold the spot-forming cell (SFC)/10^6 value obtained for the negative control (Medium) or at least 50 SFC/10^6 cells.

Table S1: Summary of premature deaths observed during the course of the previous GLP toxicology study, per experimental group.

Experimental group	Duration of follow-up	Premature deaths / total animals	Days (D) of occurrence	Comments on previous condition	Cause of death determined by histopathology
**WT + Vehicle**	8 days p.i.	1/11	D4	Low body weight. Pallor, dyspnoea, aggressivity.	Cecal ulcer, considered as the cause of the death.
	93 days p.i.	0/10	N/A	N/A	N/A
	183 days p.i.	1/10	D182	Death just after water gavage for urine collection. No clinical signs prior the death.	No lesion to report. Misgavage was considered as the cause of the death.
**WT + 6x10^{13} vg/kg**	8 days p.i.	0/10	N/A	N/A	N/A
	93 days p.i.	0/10	N/A	N/A	N/A
	183 days p.i.	0/10	N/A	N/A	N/A
**WT + 4.2x10^{14} vg/kg**	8 days p.i.	0/10	N/A	N/A	N/A
	93 days p.i.	1/10	D76	No clinical signs before death	Heart: Minimal to slight focal or multifocal inflammatory cell infiltration and multifocal to diffuse vacuolation of myofibers, occasionally accompanied by congestion Liver: Minimal to moderate centrilobular hepatocytic vacuolation and congestion → **Lesions suggest the heart being the primary organ for toxicity and death, with liver changes being anoxic in nature and consecutive to heart failure**
	183 days p.i.	7/10	D61 - D85 - D87 D93 - D121 D140 - D149	No clinical signs before death	
**TOTAL**		**10/91**			

p.i. = post-injection – N/A = not applicable

Table S2: Summary of premature deaths during the course of the present study, per experimental group.

Experimental group	Premature deaths / total animals	Death occurring during telemetry*	Animal found dead in housing cage	Day (D) of occurrence	Histopathological observations**	Cause of death determined by histopathology
**WT + Vehicle**	0 / 10	0	0	N/A	N/A	N/A
**WT + 2.1x10^{14} vg/kg**	4 / 11	0	1	D3	Thoracic and jugular hemorrhages<br>Lu: mediastinal hemorrhage<br>Li: Extra-medullary hematopoiesis<br>S: Extra-medullary hematopoiesis	Coagulation defect
		0	1	D78	Lu: Acute passive congestion	Inconclusive (autolysis of most of the tissues)
		1	0	D113	Lu: Acute vascular congestion and alveolar edema<br>K: Nephropathy (hyalin casts, glomerulopathy)	Chronic progressive nephropathy
		1	0	D137	Lu: Alveolar emphysema (terminal dyspnea)	No identified cause of death
**WT + 4.2x10^{14} vg/kg**	3 / 10	1	0	D96	Lu: Foci of spumous macrophage<br>Li: acute passive congestion	No identified cause of death
		0	1	D105	Lu: Acute passive congestion	Inconclusive (autolysis of most of the tissues)
		1	0	D180	Lu: Alveolar emphysema<br>K: Bowman capsule thickening	No identified cause of death
**TOTAL**	**7 / 31**	**4**	**3**			

Experimental group	Premature deaths / total animals	Death occurring during telemetry*	Animal found dead in housing cage	Day (D) of occurrence	Histopathological observations**	Cause of death determined by histopathology
**DMD^{mdx} + Vehicle**	3 / 11	1	0	D29	M: Dystrophic lesions + Malignant-hyperthermia lesions***<br>H: Ventricular dilatation + inflammatory foci<br>Lu: Acute passive congestion	Malignant hyperthermia-like syndrome / Cardiac failure (linked to DMD pathology)
		1	0	D54	M: Dystrophic lesions + Malignant-hyperthermia lesions<br>H: Dilated cardiomyopathy + inflammatory foci<br>Lu: Focal atelectasia<br>Li: Acute passive congestion	Malignant hyperthermia-like syndrome (linked to DMD pathology)
		1	0	D71	M: Dystrophic lesions + Malignant-hyperthermia lesions<br>H: Dilated cardiomyopathy + inflammatory foci<br>Lu: Avelolar emphysema<br>Li: Acute passive congestion	Malignant hyperthermia-like syndrome (linked to DMD pathology)
**DMD^{mdx} + 2.1x10^{14} vg/kg**	2 / 10	1	0	D60	M: Few centronucleated fibers<br>H: Right ventricular dilatation + blood vascular stasis + small inflammatory cell foci<br>Li: Acute passive congestion	Cardiac failure (linked to DMD pathology)
		0	1	D107	M: Early signs of MH<br>Lu: Alveolar emphysema (terminal dyspnea)<br>K: glomerulopathy-related lesions	Malignant hyperthermia-like syndrome (linked to DMD pathology)
**DMD^{mdx} + 4.2x10^{14} vg/kg**	4 / 10	0	1	D107	M: Dystrophic lesions + Malignant-hyperthermia lesions<br>Lu: Alveolar transudate	Malignant hyperthermia-like syndrome (linked to DMD pathology)
		0	1	D142	Mu: Very mild dystrophic lesions<br>Lu: Alveolar emphysema (terminal dyspnea)	No identified cause of death
		1	0	D157	M: Dystrophic lesions<br>H: Foci of inflammation and fibrosis<br>Lu: Alveolar emphysema (terminal dyspnea)	No identified cause of death
		0	1	D174	Mu: Very mild dystrophic lesions<br>Lu: Acute vascular congestion and alveolar edema<br>K: Hyaline casts, glomerulopathy-related lesions	Chronic progressive nephropathy
**TOTAL**	**9 / 31**	**5**	**4**			

*: cardiorespiratory arrest during handling before or after the telemetry session (i.e. installation or deinstallation of the telemetry probe + jacket, and placement in the telemetry cage) or animal found dead in its telemetry cage.
**: the following tissues were obtained and analyzed: biceps femoris muscle (BM) / heart (H) / lung (Lu) / liver (Li) / kidney (K) / spleen (S). Only observed lesions are reported in the table. If not noted, no abnormalities were detected in the other analyzed tissues.
***: Dystrophic lesions = isolated hyalin fibers, small clusters of degenerative fibers associated with muscle fiber regeneration foci, centro-nucleated fibers and anisocytosis with a few inflammatory cells in a slightly increased endomysial space corresponding to mild fibrosis. Malignant-hyperthermia lesions = large clusters of round hypereosinophilic fibers (hypercontracted fibers), some of them displaying fragmented cytoplasm and/or hyperchromatic condensed or fragmented nucleus indicative of necrosis (severe rhabdomyolysis) + huge optically empty space between fibers corresponding to massive edema.

<u>Table S3</u>: Proportion of animals presenting anti-hMD1 or anti-AAV8 cellular responses at 6 months after administration of vehicle or GNT0004 at the 4.2x10^{14} vg/kg dose. Analyses were performed on samples obtained from animals which survived to 6 months p.i. (number of positive rats / number of rats analyzed at sacrifice)

Experimental group	Number of rats positive for anti-hMD1 IgG antibodies	Number of rats positive for anti-hMD1 cellular response	Number of rats positive for anti-AAV8 cellular response
WT + Vehicle	0/10 (0%)	0/10 (0%)	0/10 (0%)
DMD^{mdx} + Vehicle	0/8 (0%)	0/8 (0%)	0/8 (0%)
**TOTAL (Vehicle-treated animals)**	**0/18 (0%)**	**0/18 (0%)**	**0/18 (0%)**
WT + 4.2x10^{14} vg/kg	1/6 (17%)	0/8 (0%)	5/8 (63%)
DMD^{mdx} + 4.2x10^{14} vg/kg	5/6 (83%)	0/6 (0%)	2/6 (33%)
**TOTAL (GNT0004-treated animals)**	**6/12 (50%)**	**0/14 (0%)**	**7/14 (50%)**

Table S4: Mean percentage of endogenous dystrophin-positive fibers *versus* hMD1-positive fibers in cardiac muscle.

Analyses were performed on samples obtained from animals which survived to 6 months p.i. Data are presented as mean ± SEM

Experimental group	Number of animals	Mean percentage of endogenous dystrophin-positive fibers (NCL-DYS2 staining)	Mean percentage of hMD1-positive fibers (NCL-DYS3 staining)
**WT + Vehicle**	10	100% ± 0.0	0.0% ± 0.0
**WT + 2.1x10^{14} vg/kg**	7	99.1% ± 1.1	93.2% ± 3.9
**WT + 4.2x10^{14} vg/kg**	7	98.2% ± 3.1	94.6% ± 14.2
**DMD^{mdx} + Vehicle**	8	3.6% ± 4.5	0.0% ± 0
**DMD^{mdx} + 2.1x10^{14} vg/kg**	8	0.8% ± 1.6	96.6% ± 2.7
**DMD^{mdx} + 4.2x10^{14} vg/kg**	6	0.0% ± 0.0	100% ± 0.0

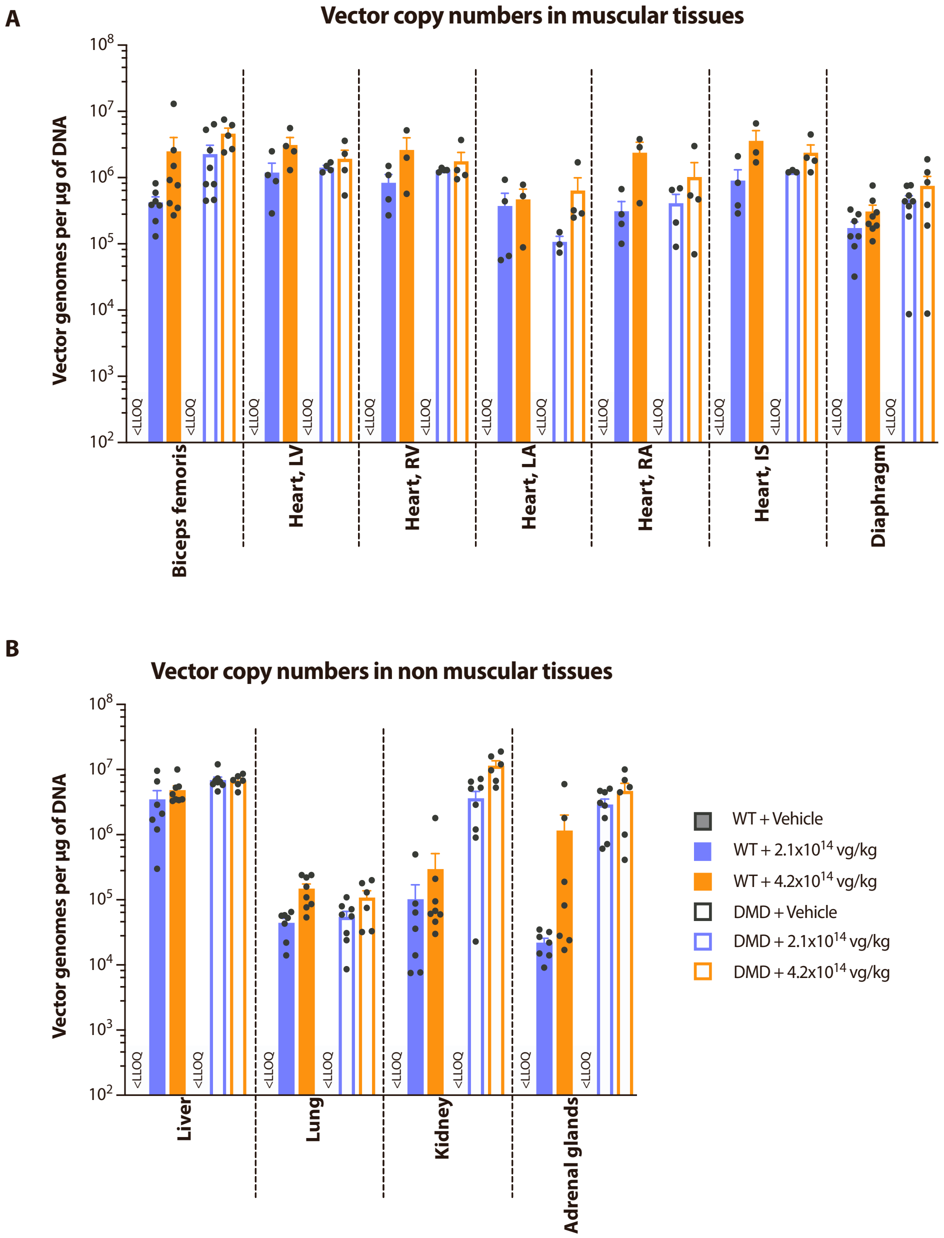

Figure S1: GNT0004 biodistribution (vector genome copies per µg of DNA) in tissue samples from WT and DMD*mdx* rats sacrificed at 6 months post-injection.

Analyses were performed on samples obtained from animals which survived to 6 months p.i. (i.e. n=3 to 5 per experimental group for the heart samples and n=6 to 10 per experimental group for the other tissues). (A) Vector genome copies per µg of DNA in muscle tissues, (B) Vector genome copies per µg of DNA in non-muscle tissues. Vector copy numbers per diploid genomes were determined using qPCR. Results are expressed as the mean ± SEM.

Statistical analyses, performed using nonparametric Kruskal-Wallis test followed by a post hoc Dunn's multiple comparisons test, showed no significant differences between the different groups injected with GNT0004. LLOQ = Lower Limit of Quantification = 50 vector genomes per µg of genomic DNA.

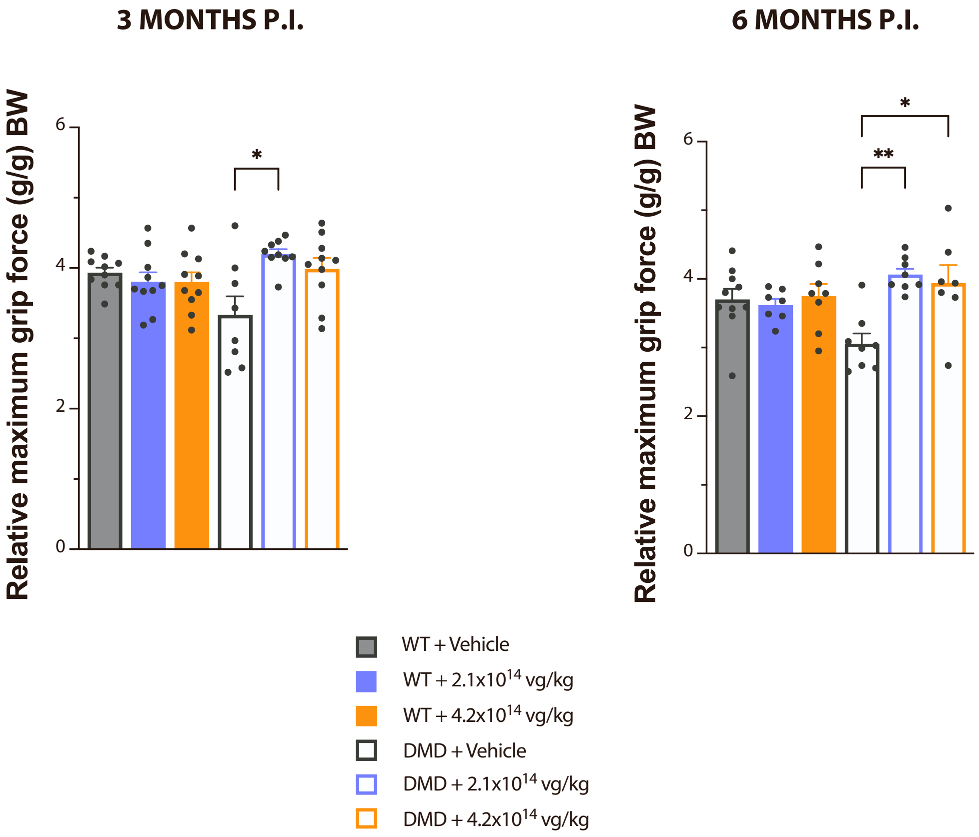

<u>Figure S2</u>: Maximum forelimb grip forces measured at 3 and 6 months after vehicle or GNT0004 administration.

Analyses were performed on animals which survived to the time of analysis (i.e. n=7 to 10 per experimental group).The highest grip force value obtained over five successive grip test trials was considered as the maximum forelimb grip force and was plotted as relative force (in g/g normalized to body weight [BW]). Data are shown as mean values ± SEM. Statistical analyses were performed using nonparametric Kruskal-Wallis test followed by a post hoc Dunn's multiple comparisons test (**$p<0.01$; *$p<0.05$).

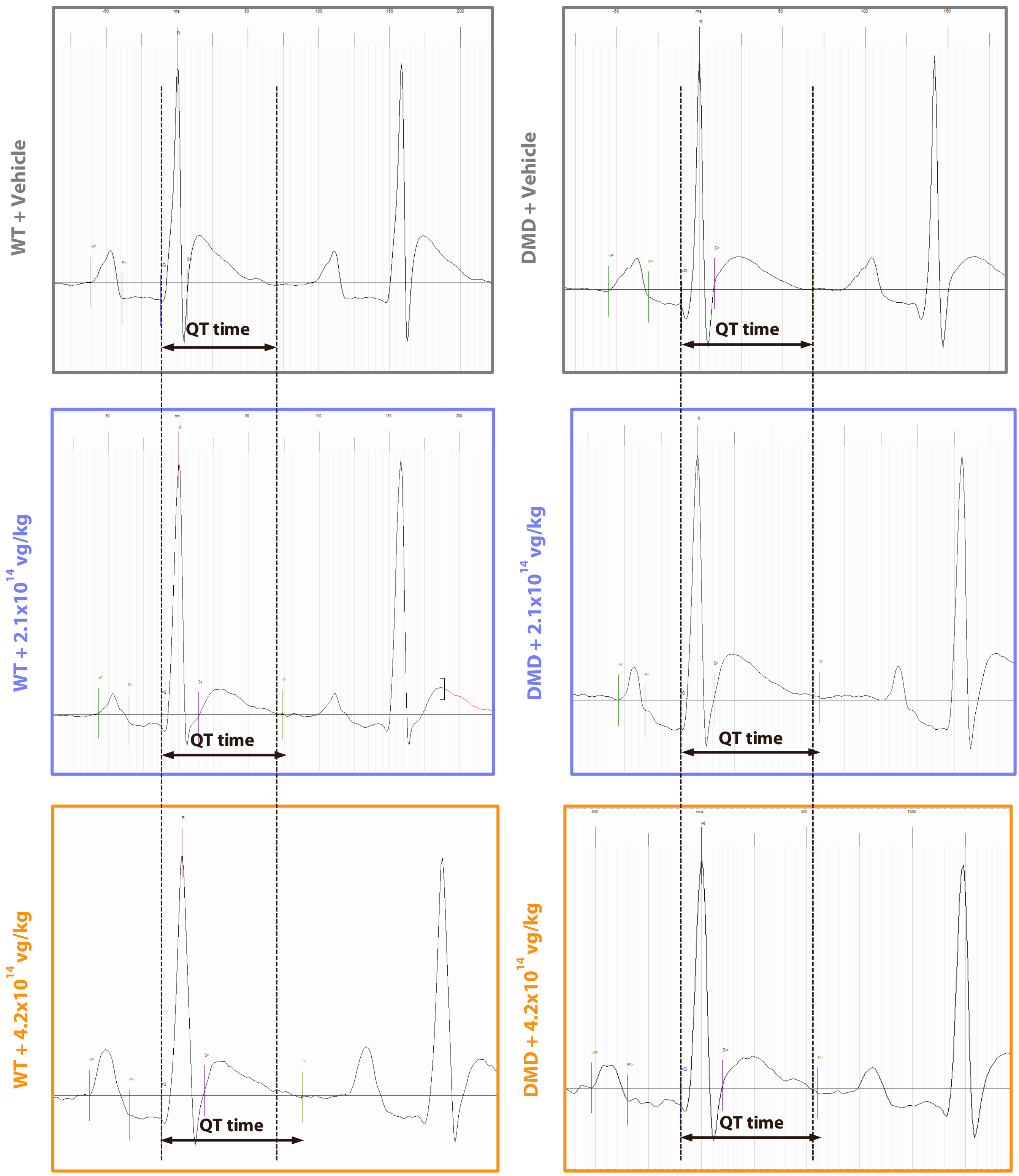

<u>Figure S3</u>: Assessment of hMD1 expression on electrocardiographic (ECG) parameters at 6 months after vehicle or GNT0004 administration. Representative ECG traces from each experimental group. QT intervals measurements are shown, with the dashed lines indicating the QT interval measured in each vehicle-injected control group.

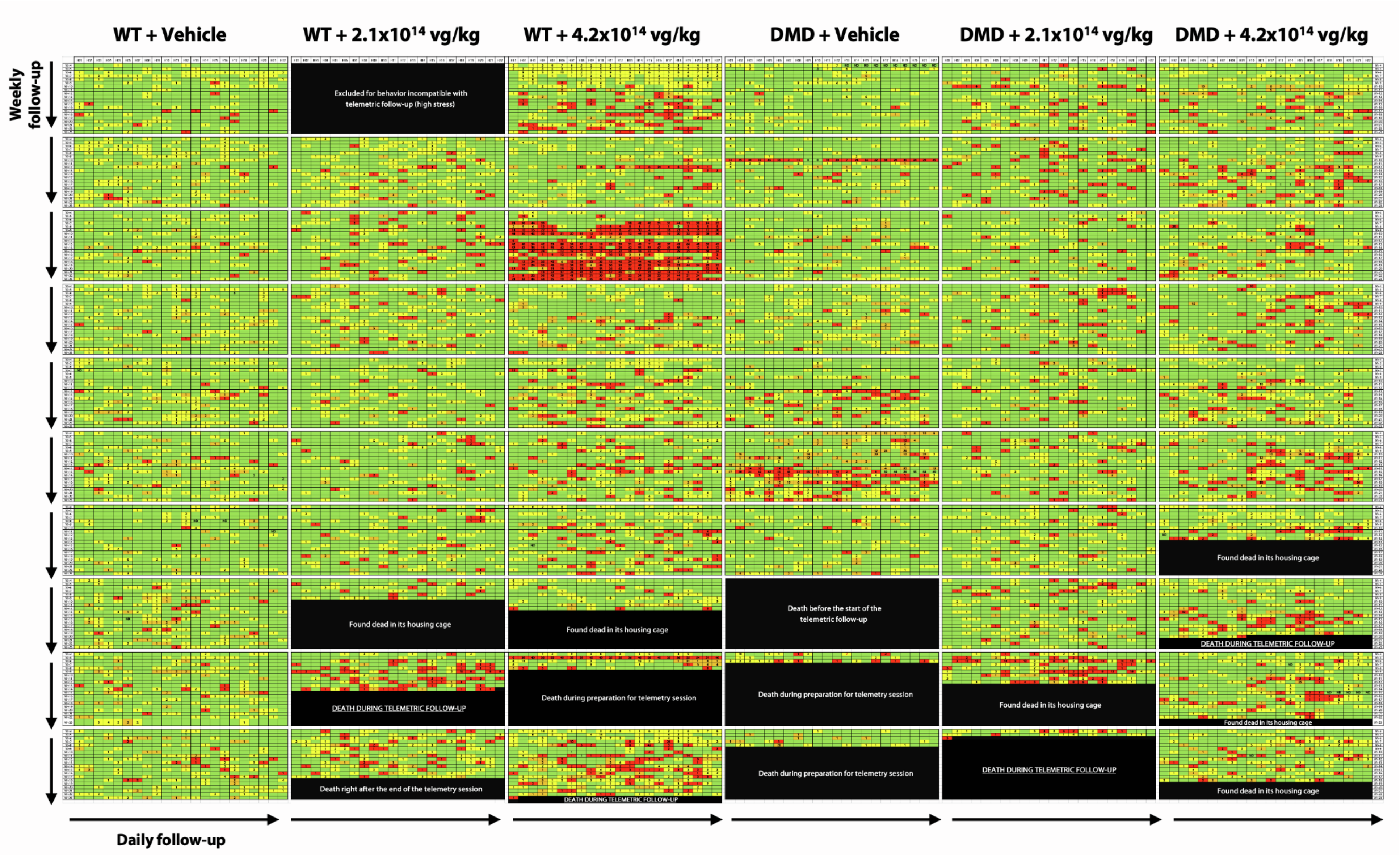

WT + Vehicle
WT + 2.1x10^14 vg/kg
WT + 4.2x10^14 vg/kg
DMD + Vehicle
DMD + 2.1x10^14 vg/kg
DMD + 4.2x10^14 vg/kg
Weekly follow-up
Daily follow-up
Excluded for behavior incompatible with telemetric follow-up (high stress)
Found dead in its housing cage
Death before the start of the telemetric follow-up
DEATH DURING TELEMETRIC FOLLOW-UP
Death during preparation for telemetry session
Death right after the end of the telemetry session

Figure S4 (previous page): Schematic representation of ECG results obtained using telemetry in the different groups of animals.

A color coding system was used to represent the rhythm disorders for each hour of ECG recorded during 20 sessions of 22 hours (daily follow-up) conducted every week during the course of the study (weekly follow-up). The events observed during each one-hour period were classified into three categories: events with low (yellow), medium (orange), or high (red) potential impact on cardiac electrical function. Absence of events are represented in green. The number of events observed each hour is indicated in the corresponding box. Black boxes denote animals that died prematurely during the study.

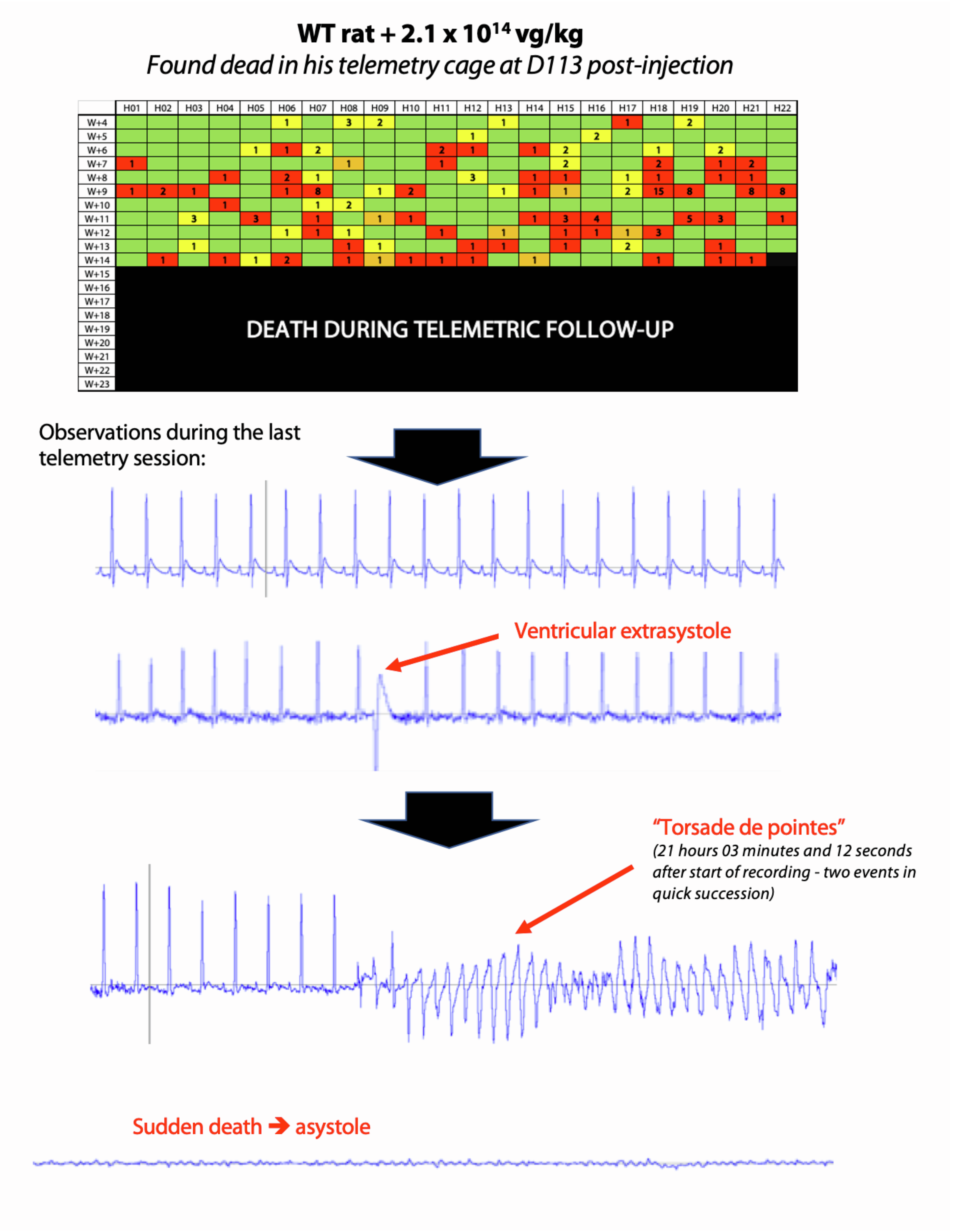

<u>Figure S5</u>: Schematic representation of ECG telemetry results in a rat from the WT + 2.1x10^{14} vg/kg group that died prematurely during a telemetry session (during the 21st hour of the 11th session).

Representative ECG traces obtained during this recording session are presented. One ventricular extrasystole was observed during this session. Major rhythm disorders were observed prior to "torsade de pointes", leading to sudden death (asystole) of the animal the following minute.

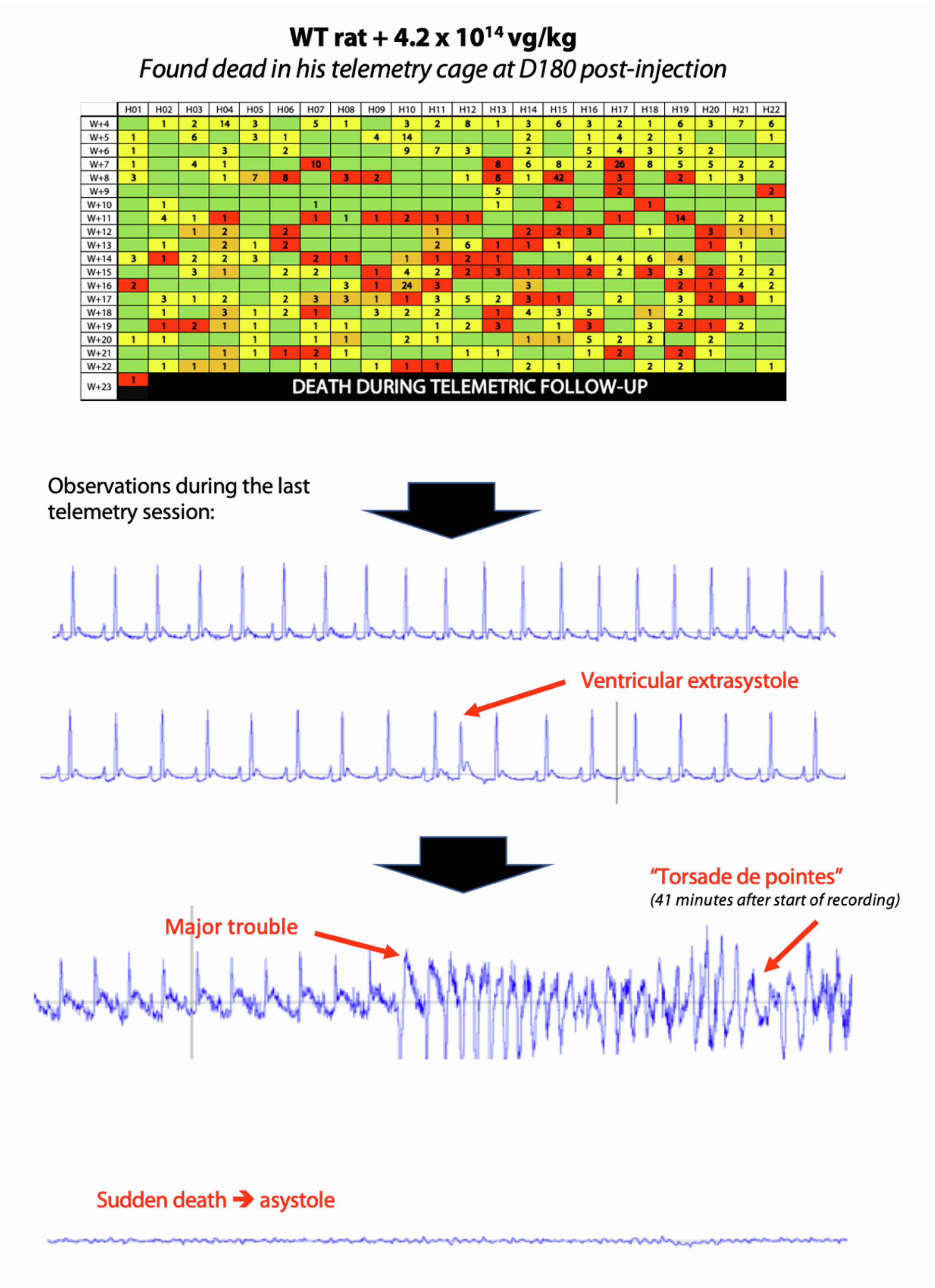

<u>Figure S6</u>: Schematic representation of ECG telemetry results in a rat from the WT + 4.2x10^{14} vg/kg group that died prematurely during a telemetry session (during the 1st hour of the 20th session).

Representative ECG traces obtained during this recording session are presented. One ventricular extrasystole was observed during this session. Major rhythm disorders were observed prior to "torsade de pointes", leading to sudden death (asystole) of the animal the following minute.

DMD^{mdx} rat + 4.2 x 10^{14} vg/kg

Found dead in his telemetry cage at D157 post-injection

	H01	H02	H03	H04	H05	H06	H07	H08	H09	H10	H11	H12	H13	H14	H15	H16	H17	H18	H19	H20	H21	H22
W+4	3			1		1			2	5			1						1			
W+5	1	2		1	1		1	2	1		1	2	1						1			
W+6		6			1		1								1							
W+7				3	2		6		1	3		1		3	3	4				1		
W+8	5	4					3				8				1			1				
W+9							4				1	1					1		1			
W+10								1		1					3	1						
W+11												3	2				2					
W+12			1	2					1						2							
W+13	3	1	32	1				2	1			1	1			2	1			4		
W+14		4			2			1	2	2	1	2		1	3	1	2				3	2
W+15	2	3		1					2	1	1	1			1	1			1			
W+16						1					1	1			3		1	1		1	1	
W+17			1	1	1		1	1		1	1			1	1	1			1			
W+18	1		1		2	1	2	2			2	2		1	1		1					
W+19			1	1	2	1			1	2			3	2			1	1	1			
W+20		1	1		4	1		2	1													
W+21	DEATH DURING TELEMETRIC FOLLOW-UP																					
W+22																						
W+23																						

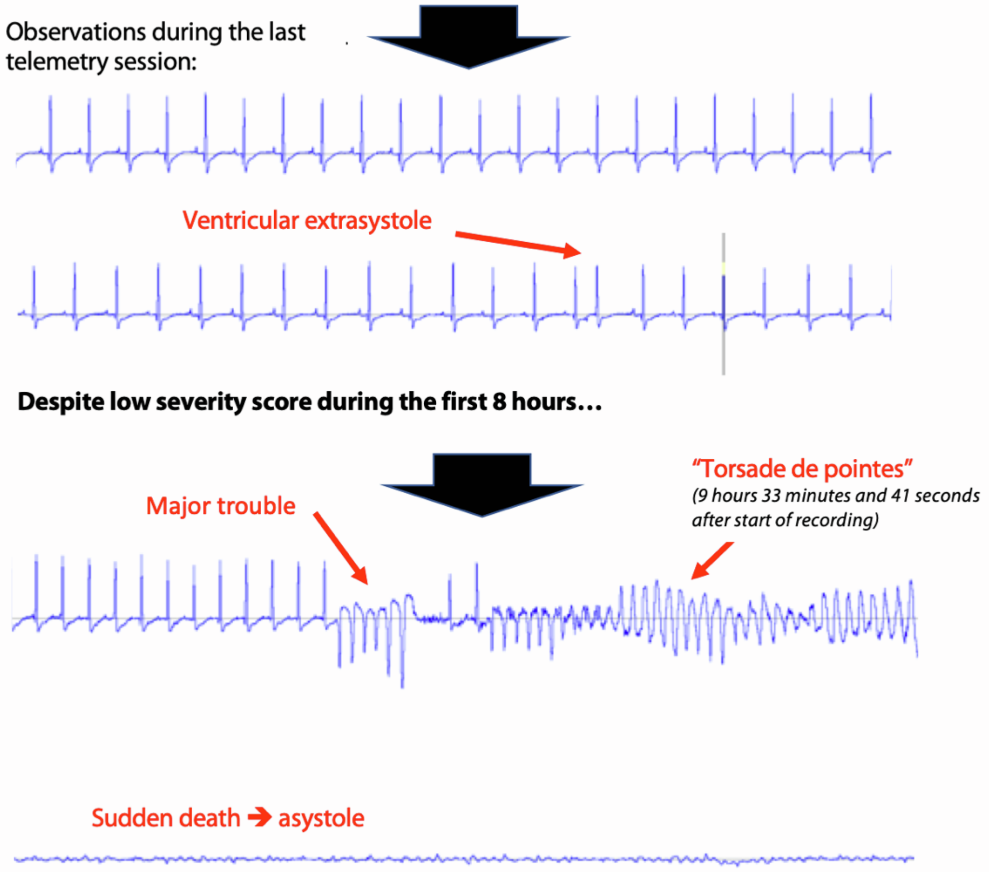

<u>Figure S7</u>: Schematic representation of ECG telemetry results in a rat from the DMD^{mdx} + 4.2x10^{14} vg/kg group that died prematurely during a telemetry session (during the 9th hour of the 17th session).

Representative ECG traces obtained during this recording session are presented. One ventricular extrasystole was observed during this session. Major rhythm disorders were observed prior to "torsade de pointes", leading to sudden death (asystole) of the animal the following minute.

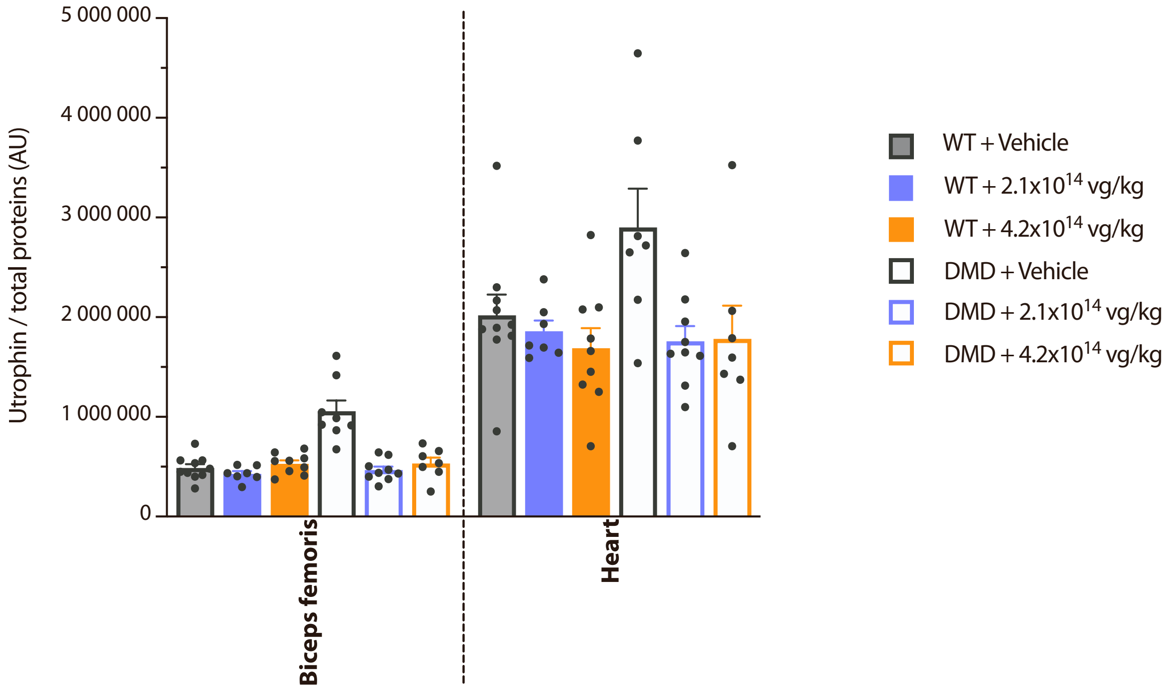

<u>Figure S8</u>: Levels of utrophin protein expression in muscles from WT and DMD*mdx* rats 6 months after administration of vehicle or GNT0004.

Analyses were performed on samples obtained from animals at 6 months p.i. (i.e. n=6 to 10 per experimental group). Quantification of utrophin protein expression by Simple Western from total proteins extracted from biceps femoris and heart. Quantification was done respective to the level of total proteins and expressed as arbitrary units (AU). Individual and mean values (± SEM) are shown. Statistical analyses, performed using nonparametric Kruskal-Wallis test followed by a post hoc Dunn's multiple comparisons test, showed no significant differences between the different groups injected with GNT0004.

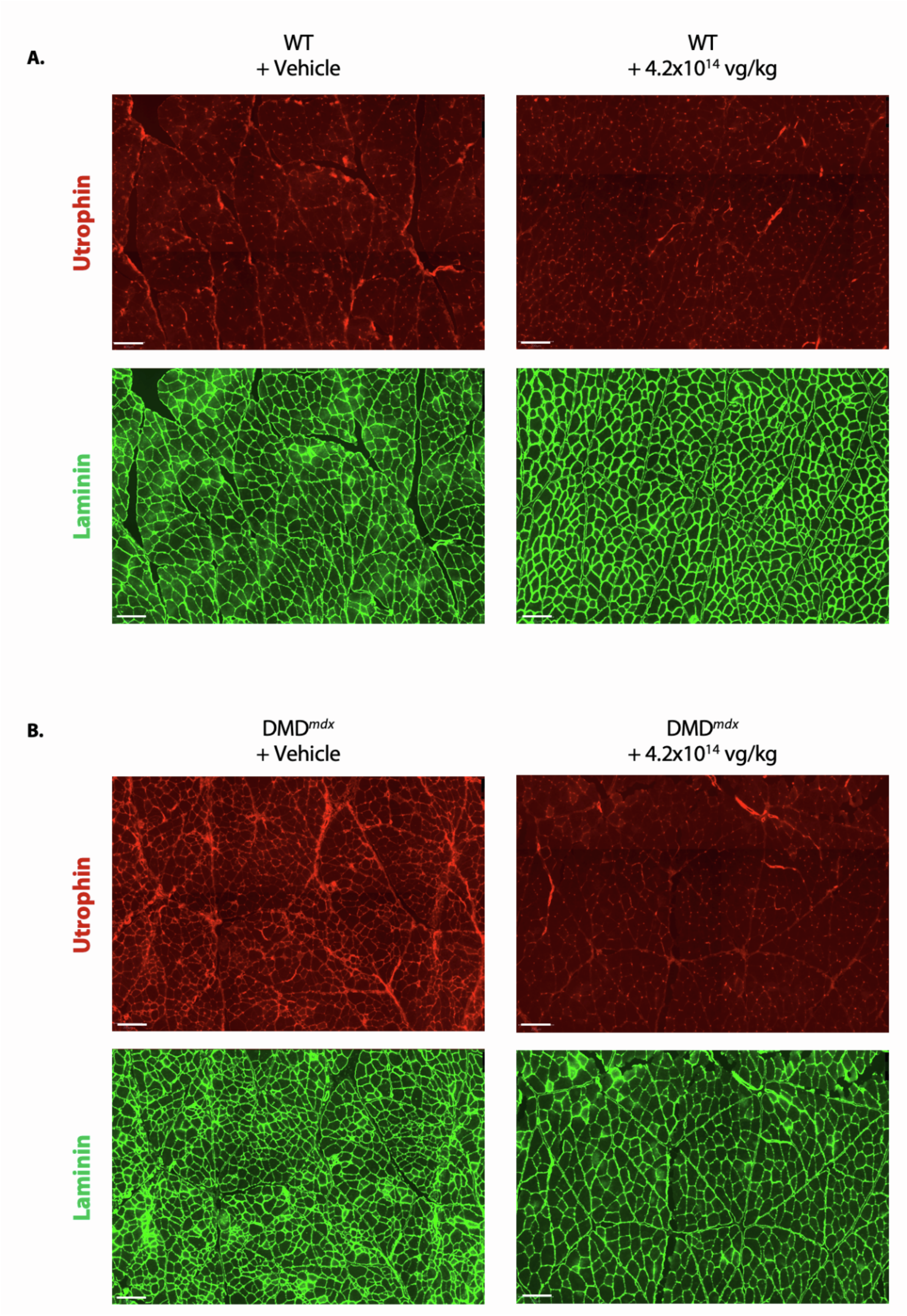

<u>Figure S9</u>: Utrophin protein immunostaining in *biceps femoris* muscle of WT and DMD^{mdx} rats 6 months after administration of vehicle or GNT0004.
Immunohistochemical analysis of utrophin expression in the *biceps femoris* of injected WT rats (A) and injected DMD^{mdx} rats (B). Representative results are presented for one animal of each experimental group. Laminin immunostaining was used to identify the basal membrane. Scale bar = 200µm.

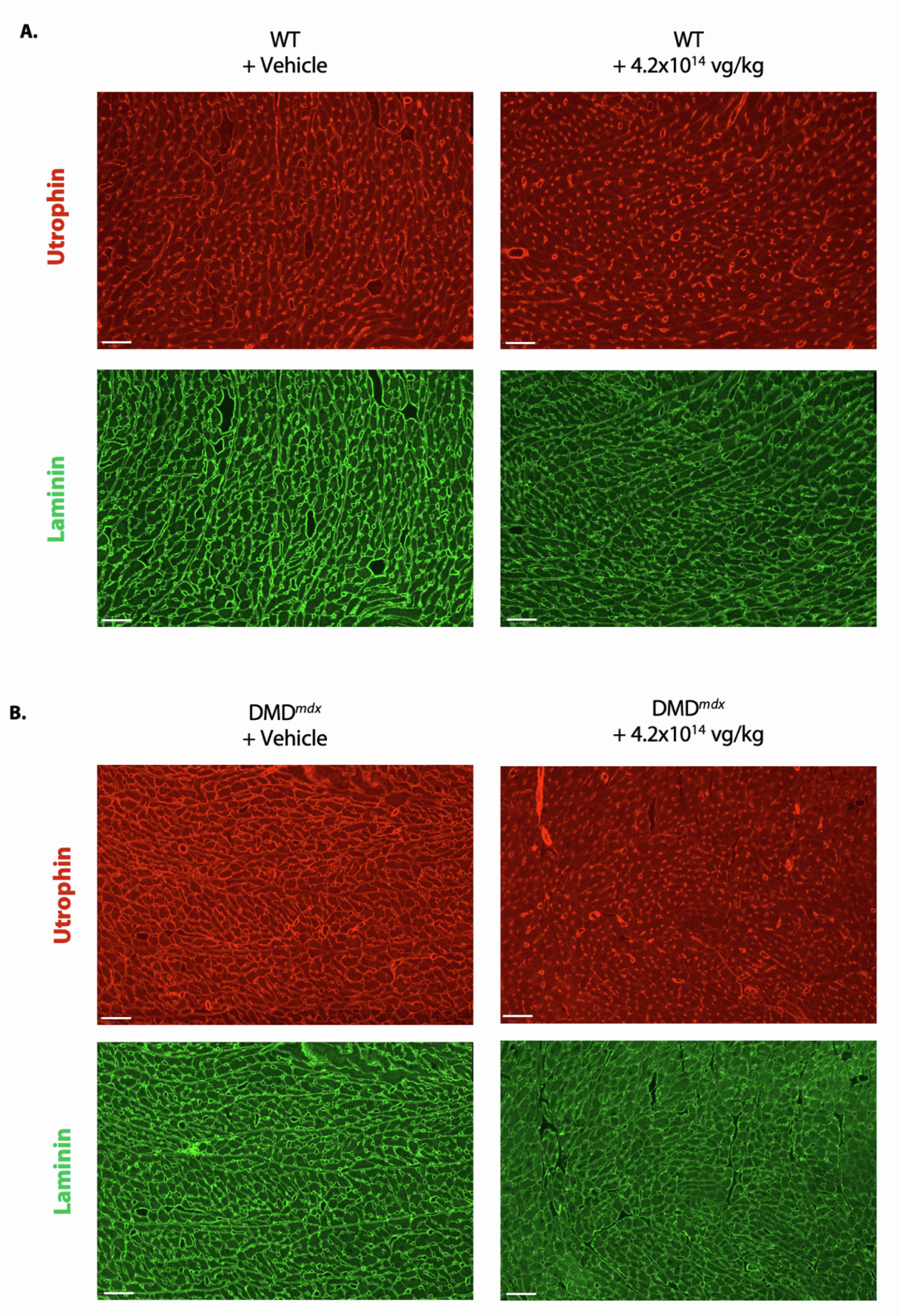

<u>Figure S10</u>: Utrophin protein immunostaining in cardiac muscle of WT and DMD^{mdx} rats 6 months after administration of vehicle or GNT0004.

Immunohistochemical analysis of utrophin expression in the hearts of injected WT rats (A) and injected DMD^{mdx} rats (B). Representative results are presented for one animal of each experimental group. Laminin immunostaining was used to identify the basal membrane. Scale bar = 200µm.
