# Supplementary material for: Dose- and genotype-dependent cardiac arrhythmia and sudden death in rats following microdystrophin gene therapy
Source: Mol Ther. 2025 Oct 24;34(1):140–60. doi: 10.1016/j.ymthe.2025.10.041 (PMC12925808; doi:10.1016/j.ymthe.2025.10.041)
Supplement: Document S2. Article plus supplemental information [file mmc2.pdf]

Molecular Therapy
Original Article

Dose- and genotype-dependent cardiac arrhythmia and sudden death in rats following microdystrophin gene therapy

Caroline Le Guiner,[1] Gilles Toumaniantz,[2] Thibaut Larcher,[3] Sylvie Marchand,[4] Laurine Buscara,[4] Grégory Cedrone,[4] Cladice Varela Moreira,[4] Amandine Lancelot,[4] Christophe Georger,[4] Aude Lafoux,[1,5] Célia Couzinié,[1,6] David Augereau,[1,7] Corinne Jounier,[1,7] Agnès Hivonnait,[2] Estelle Creoff,[4] Stéphanie Blaie,[4] Corinne Huchet,[1,5] Oumeya Adjali,[1] Nathalie Daniele,[4] Gérald Perret,[4] and Serge Braun[4,8]

[1]Nantes Université, CHU de Nantes, INSERM UMR 1089, Translational Gene Therapy Laboratory (TaRGeT), 44200 Nantes, France; [2]Nantes Université, CNRS, INSERM, l'Institut du Thorax, 44007 Nantes, France; [3]INRAE Oniris, UMR 703, PanTher, APEX, 44300 Nantes, France; [4]Genethon, 91000 Evry, France; [5]Therassay Platform, Capacités, Nantes Université, 44200 Nantes, France; [6]Capacités Biotherapeutics Solutions (CBS), Capacités, Nantes Université, 44200 Nantes, France; [7]Centre de Boisbonne, Oniris, INSERM, 44300 Nantes, France; [8]AFM Telethon, 91000 Evry, France

Recombinant adeno-associated virus (rAAV) vectors encoding microdystrophin (MD) are a promising treatment for Duchenne muscular dystrophy (DMD). GNT0004, an rAAV2/8 vector expressing MD1, is currently being tested in patients with DMD. Here, we explored supra-optimal intravenous doses of GNT0004 (2.1 $\times$ 10^{14} and 4.2 $\times$ 10^{14} vg/kg, up to 14 times the therapeutic dose) in wild-type (WT) and DMD^{mdx} rats. In all cohorts, robust MD1 protein expression was observed. In DMD animals, creatine kinase levels were normalized, and skeletal muscle and heart histology and functions were improved. However, unexpected sudden deaths occurred at the highest dose. In WT animals, deaths were observed at both doses and were associated with increased arrhythmic events, which may promote structural and functional heart issues. Immunohistological analysis suggested that overexpression of MD1 may disrupt the dystrophin-associated protein complex, increasing the risk of arrhythmias and sudden death. In DMD^{mdx} rats, the 2.1 $\times$ 10^{14} vg/kg dose was well tolerated, but some deaths occurred at 4.2 $\times$ 10^{14} vg/kg, for which a causal link to GNT0004 cannot be excluded. At this dose, increased arrhythmic risk and cardiac pathological remodeling were observed. These observations highlight the potential risk of MD overexpression in the heart and suggest a need for careful monitoring of patients with DMD treated with gene therapy.

INTRODUCTION

Duchenne muscular dystrophy (DMD) is a lethal X-linked recessive neuromuscular disorder caused by mutations in the *DMD* gene. These mutations result in absent or non-functional dystrophin, a 427-kDa cytoskeletal protein that enables the stability, functionality, and strength of myofibers in skeletal and cardiac muscles.[1] Dystrophin establishes a mechanical link between cytoskeletal actin and the extracellular matrix through the dystrophin-associated protein complex (DAPC).[2] When dystrophin is absent, the mechanical and signaling functions of the skeletal, smooth, and cardiac muscle fibers are compromised.[3,4] Most individuals affected by DMD (incidence of 1/3,500–5,000 male births) are diagnosed before the age of 5, when their physical decline becomes apparent.[5,6] As muscle weakness progresses, patients typically experience a decline in walking distance around the age of 7 and lose independent ambulation by their early teens. Despite several palliative treatment options, the progressive muscle damage leads to a definitive loss of muscle function and, ultimately, to life-threatening cardiac and/or respiratory failure in the patient's third or fourth decade.[7]

Historically, US Food and Drug Administration (FDA)-, European Medicines Agency (EMA)-, and/or Pharmaceuticals and Medical Devices Agency (PMDA)-approved treatments for DMD were based on glucocorticoid derivatives with only moderate therapeutic effects. The mechanisms of action of these therapies are not yet fully elucidated but are assumed to be linked to their anti-inflammatory properties.[8,9] More recently, RNA-targeted therapies, such as exon-skipping agents and compounds promoting ribosomal read-through of premature stop codons, have been approved for the treatment of DMD.[10,11] Their approval was primarily based on a modest increase of truncated dystrophin in skeletal muscle (a less than 6% increase compared to baseline).[12] Furthermore, these RNA-targeting drugs are indicated only for specific genetic subtypes representing a small fraction of patients with DMD. Nonsense stop

Received 15 March 2025; accepted 15 October 2025;
https://doi.org/10.1016/j.ymthe.2025.10.041.

Correspondence: Caroline Le Guiner, Nantes Université, CHU de Nantes, INSERM UMR 1089, Translational Gene Therapy Laboratory (TaRGeT), 44200 Nantes, France.
E-mail: caroline.le-guiner@univ-nantes.fr
Correspondence: Serge Braun, Genethon, 91000 Evry, France.
E-mail: sbraun@afm-telethon.fr

Published by Elsevier Inc. on behalf of The American Society of Gene and Cell Therapy.
This is an open access article under the CC BY license (http://creativecommons.org/licenses/by/4.0/).

codon mutations account for ∼10% of patients, while the combined exon-skipping drugs are adapted for only ∼36% of patients with DMD.[3,13] More recently, gene replacement therapy, a direct way to restore expression of the dystrophin protein, has shown promising results. The current state-of-the-art viral vectors for gene therapy are the ones derived from recombinant adeno-associated viruses (rAAVs). rAAVs can efficiently spread in skeletal and heart muscle tissues and have advantageous safety profiles over other viruses, including their non-integrative nature, which reduces the risk of genotoxicity.[14,15] As the gene encoding the full-sized dystrophin protein exceeds the packaging capacity of a single rAAV vector, microdystrophin (MD) genes have been designed to fit inside rAAV vectors and yet include critical domains to maintain protein function.[16,17] Based on convincing widespread expression of MD in most myofibers and significant reduction of pathological dystrophic features in rodent and dog DMD models,[18–25] several clinical trials are underway to evaluate the safety and efficacy of systemic administration of different rAAV vectors encoding various forms of MD, under the control of different promoters.[26–28] Recently, one of these AAV-MD drug candidates, Elevidys, has been approved by the FDA for the treatment of ambulatory and non-ambulatory pediatric patients of at least 4 years of age[29] (https://investorrelations.sarepta.com/news-releases/news-release-details/sarepta-therapeutics-announces-expanded-us-fda-approval-elevidys).

Among the other investigational medicinal products, GNT0004 is a genetically modified rAAV vector composed of a serotype 8 capsid and a serotype 2 backbone containing a transgene composed of the single-stranded codon-optimized complementary DNA (cDNA) sequence encoding for the human MD (hMD1) protein,[18] under the transcriptional control of the synthetic muscle and cardiac restricted promoter Spc5.12[30–32]: rAAV2/8-Spc5.12-hMD1. GNT0004 (also referred to as AAV8-hMD1) is currently being investigated in a phase 3 multicentric clinical trial involving a single intravenous (i.v.) administration in ambulant boys with DMD aged 6 to 10 years old.

Several preclinical studies were performed to support the Investigational New Drug (IND) applications of GNT0004. In a study performed in the golden retriever muscular dystrophy (GRMD) dog model, systemic i.v. administration without immunosuppression resulted in significant and sustained levels of canine MD1 in skeletal muscles throughout the body (40%–80% of cMD1+ positive fibers) and reduced dystrophic clinical symptoms for more than 10 years without any safety issues.[19,23] A dose-optimization study was then carried out in a DMD^{mdx} rat model that mimics both the skeletal and cardiac alterations (cardiac hypertrophy followed by an initiation of dilated cardiomyopathy) observed in patients with DMD.[33] This exploratory dose study showed favorable safety at all doses tested (1×10^{13}, 3×10^{13}, 6×10^{13}, and 1×10^{14} vg/kg) up to 6 months after injection, the restoration of muscle and cardiac histology and function, and normalization of the circulating biomarkers. Based on these data, a minimum effective dose (MED) was defined in the range of 1×10^{13} to 3×10^{13} vg/kg (data not shown). A Good Laboratory Pratice (GLP)-compliant toxicology study was then performed in Sprague-Dawley wild-type (WT) male rats. Two different doses of GNT0004 (6×10^{13} vg/kg, corresponding to the highest intended-to-treat dose, and 4.2×10^{14} vg/kg) were injected i.v. Between 2 and 5 months post-injection (p.i.), several cases of sudden death were observed in the 4.2×10^{14} vg/kg group, without prior (clinical or biological) signs of toxicity. No fatal cases were observed in the 6×10^{13} vg/kg group. It was postulated that the sudden deaths observed after the injection of the 4.2×10^{14} vg/kg dose might have been secondary to cardiac dysfunction. To further characterize and understand the potential cardiac toxicity after injection of this dose, as well as to ensure a comprehensive safety assessment, including identification of the no observable adverse effect level (NOAEL), the effects of the 2.1×10^{14} and 4.2×10^{14} vg/kg GNT0004 doses were evaluated in both WT and DMD^{mdx} rats.

RESULTS

Previous observations in toxicology study

Prior to the present study, a GLP-compliant toxicology study was conducted on healthy 4-week-old specific-pathogen-free (SPF) male Sprague-Dawley WT rats. The objective was to assess the toxicity and biodistribution of GNT0004. Two i.v. dose levels were selected: 6×10^{13} vg/kg, corresponding to the highest intended therapeutic dose, and 4.2×10^{14} vg/kg, which represents a 7-fold higher dose than the projected maximum dose for human use. Animals were monitored and euthanized at three time points p.i.: days 8, 93, and 183. Each experimental group (i.e., dose × time point) included 10–11 animals, for a total of 91 animals. The following parameters were evaluated: morbidity/mortality, full clinical observations, functional and neurobehavioral assessments, body weight (BW), food consumption, ophthalmologic examination, hematology and coagulation profiles, clinical chemistry and urinalysis, complement activation, circulating inflammatory cytokine levels, anti-transgene and anti-rAAV8 immune responses, vector shedding, biodistribution, and transgene expression. Following GNT0004 administration, dose-related vector biodistribution was observed in blood and across all standard toxicology tissues, with transgene expression in muscle tissues. A dose-related humoral immune response targeting both the AAV8 capsid and the human transgene was detected. However, this immune activation was not accompanied by systemic inflammation and did not prevent long-term transgene expression (data not shown). While the 6×10^{13} vg/kg dose was well tolerated and free of adverse effects, administration of 4.2×10^{14} vg/kg resulted in spontaneous mortality in 8 out of 20 animals, starting approximately 2 months p.i., in the absence of preceding clinical signs (Table S1). In these high-dose animals, reduced BW and food consumption were observed, alongside increased liver weight, suggestive of metabolic dysfunction. At day 93 p.i., animals receiving the high dose exhibited cardiac abnormalities, including increased heart weight, inflammatory cell infiltration, and myofiber vacuolation (data not shown). In animals found dead, similar microscopic cardiac changes were observed, as well as centrilobular hepatic vacuolation and liver congestion. According to the interpretation of a certified veterinary pathologist, the primary toxic

effects were likely cardiac, with hepatic alterations considered secondary to cardiac dysfunction (Table S1). These results indicated that a single i.v. injection of GNT0004 at 6 × 10^{13} vg/kg is safe in WT rats and is defined as the NOAEL. In contrast, the 4.2 × 10^{14} vg/kg dose was associated with adverse cardiac toxicity, warranting further investigation to better characterize and understand the underlying mechanisms.

Comparative GNT0004 safety in WT rats and DMD^{mdx} rats: Study design

In the present study, GNT0004 was i.v. administered in both 1-month-old WT and DMD^{mdx} rats at two high doses: 4.2 × 10^{14} and 2.1 × 10^{14} vg/kg. The 4.2 × 10^{14} vg/kg dose was the highest tested dose, causing severe adverse events in WT rats during the GLP-compliant toxicology study. The 2.1 × 10^{14} vg/kg dose was selected as an intermediate dose between the higher dose and 1 × 10^{14} vg/kg, previously identified as safe and efficient in DMD^{mdx} rats (Genethon project team, unpublished data). Controls were age-matched vehicle-injected WT and DMD^{mdx} rats. A total of 62 animals (31 DMD^{mdx} rats and 31 WT rats, 10–11 animals per experimental group) were included in this comparative study with a 6-month follow-up period. The animals underwent regular clinical assessments and analyses of skeletal muscle and cardiac function, including weekly non-invasive telemetry-based electrocardiograms (ECGs) on conscious animals. After euthanasia at 6 months p.i., vector copy numbers in tissues, muscle histology, expression levels of exogenous hMD1, expression levels of endogenous rat dystrophin (rDys), DAPC composition, levels of circulating DMD biomarkers, and immune responses were analyzed.

Genotype-specific premature deaths observed in WT and DMD^{mdx} rats after GNT0004 administration

During the study, several premature deaths occurred, and these are summarized in Table S2. Of the 62 animals included in the study, 16 died prematurely. Except for one animal that was found dead 3 days p.i., the deaths occurred between 1 and 6 months p.i. (median: 107 ± 43 days). No death was observed in the WT + vehicle group, while 3 deaths were observed in the DMD^{mdx} + vehicle group. After GNT0004 injection, deaths were observed in both WT and DMD^{mdx} rats and with both doses. The global incidence of premature deaths did not significantly differ between WT (7 deaths/21 animals injected with GNT0004) and DMD^{mdx} (6 deaths/20 animals injected with GNT0004) rats.

Autopsies were performed shortly (<4 h) after discovery of death. Histopathology analyses on a series of key tissue samples (*biceps femoris* muscle, heart, lung, liver, kidney, and spleen) were used to determine the cause of death. As presented in Table S2, histopathological findings showed that the cause of death appeared to be different between WT and DMD^{mdx} animals. In DMD^{mdx} rats, most of the deaths (7/9, i.e., 3/3 in the DMD^{mdx} + vehicle group, 2/2 in the DMD^{mdx} + 2.1 × 10^{14} vg/kg group, and 2/4 in the DMD^{mdx} + 4.2 × 10^{14} vg/kg group) were concluded to be unrelated to GNT0004 treatment but were instead linked to the DMD pathology itself or to non-specific lesions (such as chronic progressive nephropathy lesions), commonly observed in male Sprague-Dawley rats.[34] Five of the DMD^{mdx} rats, including those injected with vehicle, had muscle lesions typical of malignant hyperthermia-like syndrome, a syndrome that is described in both patients and animal models with myopathy.[35,36] Stress can specifically trigger malignant hyperthermia, particularly in DMD animal models that exhibit increased sensitivity to stress.[37,38] The occurrence of death in some animals during manipulation and telemetry ECG jacket measurements strongly suggests that these events were likely attributable to the high level of stress caused by this procedure. Finally, one DMD^{mdx} rat injected with the 2.1 × 10^{14} vg/kg dose died of acute cardiac failure after decompensation, a phenomenon previously observed and linked to the DMD pathology itself.[33,39] These results suggest that in DMD^{mdx} animals, most of the deaths observed after the injection of GNT0004 were related to the DMD pathology and not to the administration of GNT0004. However, a link to GNT0004 administration cannot be dismissed for two of the deaths observed after the injection of 4.2 × 10^{14} vg/kg. In contrast, a link with the GNT0004 product cannot be excluded for most WT premature deaths. Only one case was attributed to non-specific lesions consistent with chronic progressive nephropathy. For the remaining cases, the involvement of GNT0004 cannot be ruled out: one animal exhibited an early coagulation defect (3 days p.i. with the 2.1 × 10^{14} vg/kg dose), while the cause of death in the others could not be determined by the histopathological analyses performed.

The potential biological processes that could account for these unexplained deaths, in both WT and DMD^{mdx} rats, were next explored.

GNT0004 high dose negatively impacts BW gain

Besides the premature deaths previously described, no additional adverse clinical events could be attributed to GNT0004 injections. Clinical signs of stress were, however, observed in all animals due to the manipulation and restraints during telemetry sessions, particularly in the placement and removal of ECG jackets. In particular, chromodacryorrhea, a clinical sign associated with discomfort and stress, was occasionally noted. Chromodacryorrhea was exclusively associated with telemetry sessions (ECG recording) and, therefore, was not attributed to GNT0004. However, BW curves (Figure 1) showed that, regardless of genotype (WT or DMD^{mdx}), the 4.2 × 10^{14} vg/kg dose rats' weight gain was lower than that of their vehicle-treated counterparts, with the greatest impact observed in WT animals. Conversely, the 2.1 × 10^{14} vg/kg GNT0004 dose had no impact on the weight curve of WT animals, while it showed a positive trend (i.e., improvement of BW) in DMD^{mdx} animals.

GNT0004 gene transfer efficacy was similar between WT and DMD^{mdx} rats

Using a quantitative PCR (qPCR) assay, with a probe specific for the transgene sequence, GNT0004 vector genome copy numbers (VCNs) were evaluated in tissue samples obtained at euthanasia (i.e., at 6 months p.i.). VCNs were detected and quantifiable in all GNT0004-treated animals, whereas all specimens from vehicle-treated

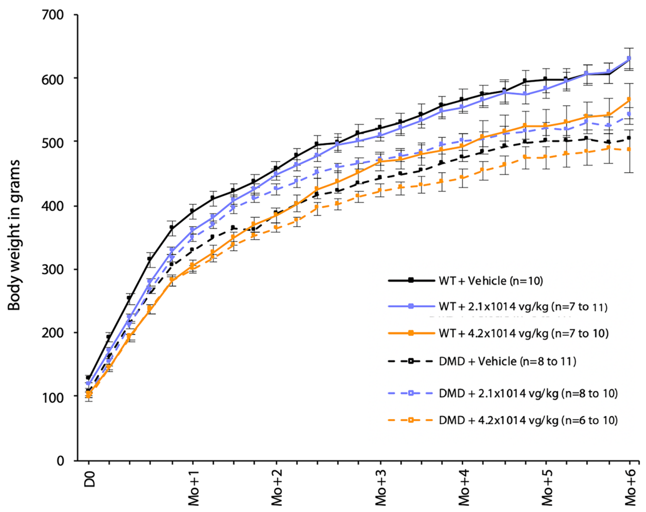

Figure 1. Follow-up on WT and DMD^{mdx} rat body weights after injection with vehicle or GNT0004 product

Data are shown as mean ± SEM. Mo, month.

animals were found below the qPCR assay's lower limit of detection (Figure S1). In muscle tissues, similar patterns of biodistribution and dose effects were observed in WT and DMD^{mdx} treated rats. Skeletal muscles (*biceps femoris* and diaphragm) and heart regions (both ventricles and atria and the interventricular septum) showed comparable VCN levels (between $\sim 1 \times 10^5$ and $\sim 4 \times 10^6$ vg/µg of DNA, depending on the injected dose) (Figure S1A). In non-muscle tissues, the highest VCN levels were observed in the livers of both WT and DMD^{mdx} rats and the kidneys and adrenal glands of DMD^{mdx} rats (between $\sim 3 \times 10^6$ and $\sim 1 \times 10^7$ vg/µg of DNA, depending on the injected dose). Lower VCNs were observed in the lungs (between $\sim 4 \times 10^4$ and $\sim 2 \times 10^5$ vg/µg of DNA, depending on the injected dose). Besides the one-to-two-log higher VCN levels in the kidneys and adrenal glands of the DMD^{mdx} rats, no differences were observed between WT and DMD^{mdx} rats (Figure S1B).

GNT0004 induced high expression of hMD1 protein in skeletal muscles and heart, independent of dose and genotype

For the animals that survived to 6 months p.i., expression of the transgenic hMD1 protein encoded by GNT0004 was assessed in the *biceps femoris* muscle, heart, and diaphragm. Western blot analysis showed that, as expected, no hMD1 protein was detected in any tissues from vehicle-treated WT or DMD^{mdx} rats. Conversely, all animals that received GNT0004 (4.2×10^{14} or 2.1×10^{14} vg/kg) expressed the hMD1 protein regardless of genotype (WT or DMD^{mdx}) (Figure 2A).

In parallel to this semi-quantitative western blot analysis, a more accurate quantification of hMD1 expression was performed using Simple Western (Figure 2B). Although slightly higher dystrophin signals were seen in the *biceps femoris* and diaphragm of DMD^{mdx} animals treated with the highest dose (4.2×10^{14} vg/kg) and slightly lower signals in the heart of animals treated with the intermediate dose (2.1×10^{14} vg/kg), none of these differences reached statistical significance. This is likely due to intra-group variability, particularly in the high-dose groups. Overall, similar levels of dystrophin expression were observed across the different doses, regardless of the animal genotype or tissue analyzed. These findings support the hypothesis of transgene expression saturation at doses $\geq 2.1 \times 10^{14}$ vg/kg.

Despite high levels of VCNs being found in the livers, kidneys, and adrenal glands of GNT0004-treated rats (see Figure S1), no hMD1 protein was detected in these tissues. This is consistent with the muscle-specific promoter (Spc5.12) used in the rAAV.

The expression and localization of the transgenic hMD1 protein encoded by GNT0004 were also analyzed by immunohistochemistry in the *biceps femoris* muscle and heart. The NCL-DYS3 antibody, which targets parts of the H1-R1 domains of the human dystrophin protein and does not cross-react with native rDys, was used (Figures 2C and 2D). As expected, no hMD1 expression was seen in vehicle-treated animals. In all GNT0004-treated WT and DMD^{mdx} rats, intense, continuous subsarcolemmal hMD1 expression was seen in >90% of the muscle fibers. Expression of hMD1 in 100% of the fibers in the *biceps femoris* of the WT + 4.2×10^{14} vg/kg group and in the *biceps femoris* and heart of the DMD^{mdx} + 4.2×10^{14} vg/kg group was even achieved. Focal subsarcolemmal-positive deposits were sporadically observed in animals treated with the 4.2×10^{14} vg/kg dose, possibly reflecting abnormal accumulation of the hMD1 protein. These observations were not associated with any other morphological alterations of these fibers.

Peripheral anti-hMD1 and anti-rAAV8 immune response elicited by GNT0004 delivery did not induce a deleterious effect

Peripheral humoral and cellular immune responses against hMD1 and cellular immune responses against the AAV8 capsid were assessed in serum samples and splenocytes obtained at 6 months p.i. from animals treated with vehicle or with the highest dose (4.2×10^{14} vg/kg) of GNT0004 (Table S2). No humoral or cellular immune response was detected in vehicle control animals regardless of genotype. Anti-hMD1 immunoglobulin (Ig)G antibodies, analyzed by western blot on serum samples, were detected in one and five of the six GNT0004-injected WT and DMD^{mdx} rats, respectively. This suggested a higher susceptibility of dystrophin-negative animals toward the xenogenic hMD1 protein. The results of an interferon (IFN)γ ELISpot assay suggest that anti-hMD1 cellular immunity was not detected in any animal. In contrast, a cellular anti-AAV8 immunity (with IFNγ secretion) was found in five of the eight WT rats and two of the six DMD^{mdx} rats injected with GNT0004.

The variability of the immune responses in the treated animals may be related to individual immune responsiveness and influenced by the genetic heterogeneity of the outbred Sprague-Dawley rat strains used in the study, as shown in other models.[40,41] Importantly, no deleterious effects on histology (see Figure 3) or transgene expression (see Figure 2) were observed in the animals that developed these

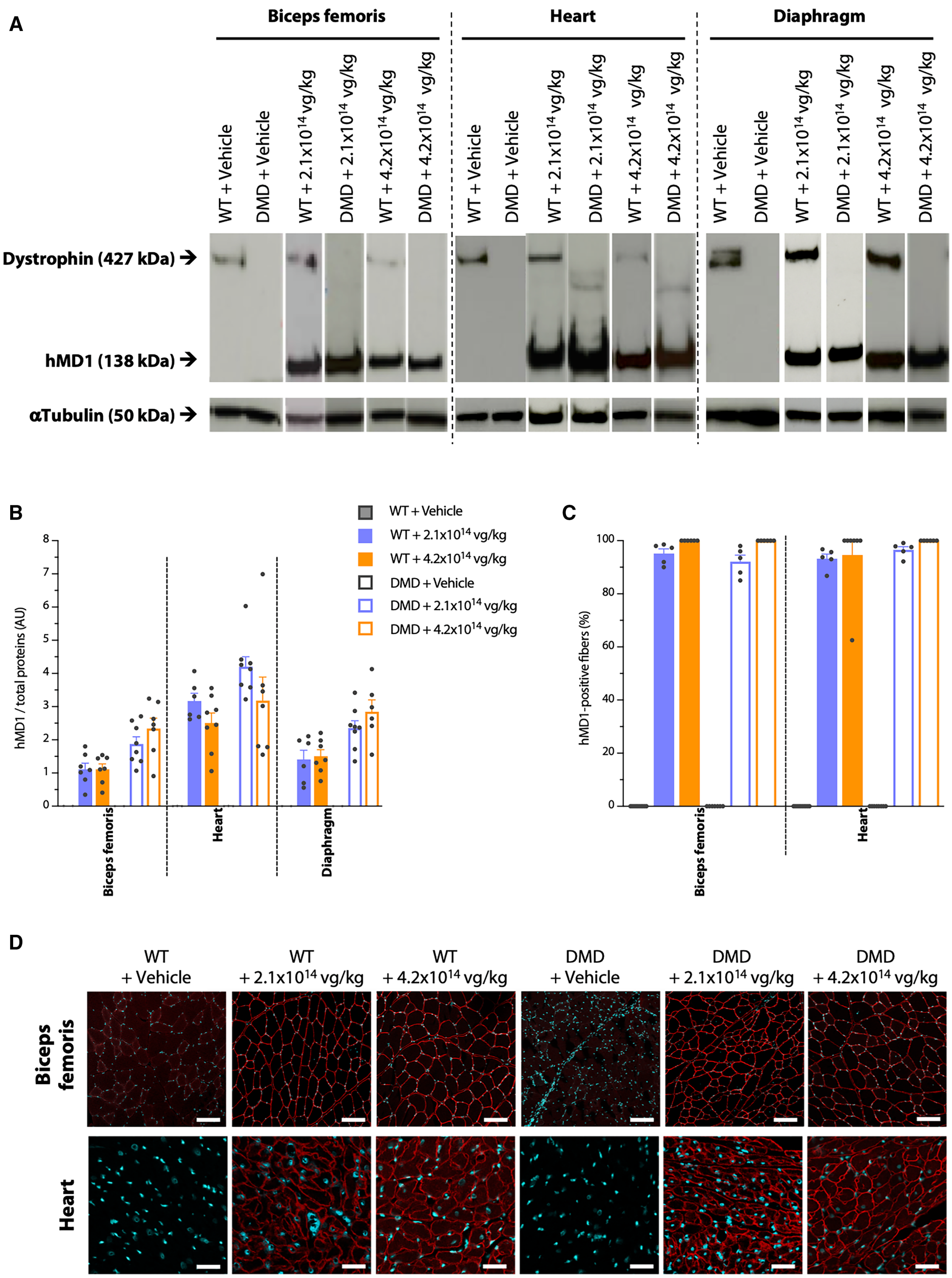

Figure 2. hMD1 protein in WT and DMD^{mdx} rats' muscles 6 months after administration of vehicle or GNT0004

Analyses were performed on samples obtained from animals still alive at 6 months p.i. (i.e., *n* = 6–10 per experimental group). (A) Western blot analysis of hMD1 expression on total proteins extracted from *biceps femoris*, heart, and diaphragm of injected rats. Representative results are presented for one animal of each experimental group. Each blot was stained with MANEX-1011C to reveal the presence of the 427-kDa dystrophin protein (in WT rat) and the 138-kDa hMD1 protein. Anti-α-tubulin antibody was used as the

(legend continued on next page)

immune responses. No link between the observed unexpected sudden deaths and the immune responses could be identified.

Improved muscle pathology and circulating biomarkers specific to DMD^{mdx} rats after GNT0004 delivery

Histopathological evaluations were performed on skeletal muscles (including diaphragm) and the heart, as well as on non-muscle tissues (i.e., liver, lung, kidney, adrenal glands, and aorta), 6 months p.i. In non-muscle tissues, no lesions that could be linked to the test item were observed in any of the animals (data not shown). Muscle lesions were analyzed to detect any differences in the histopathological patterns between the different experimental groups. Muscles from all vehicle-treated DMD^{mdx} rats analyzed exhibited myopathic lesions typical of the DMD^{mdx} rat model.[22,33] The myopathic lesions were composed of the presence of regenerative activity, as evidenced by centronucleated fibers and small foci of regeneration; the presence of degenerated fibers, isolated or in small clusters; tissue remodeling; and fiber replacement by fibrotic or adipose tissues. The severity of the lesions varied considerably between individuals and muscle types. In view of this level of variability and to provide a global evaluation of the total dystrophic lesions, skeletal and cardiac muscle lesions were semi-quantitatively scored on a scale of 0 (no lesion) to 3 (severe lesions). A mean individual muscle lesion score was then calculated based on the scores obtained from the *biceps femoris*, diaphragm, and cardiac muscle. As expected, the data showed a statistically significant difference between the WT groups (vehicle or GNT0004 treated) and the DMD^{mdx} + vehicle group. The global histopathological lesion score in WT animals, regardless of treatment, was unchanged 6 months p.i., while an improvement of this score was observed in DMD^{mdx} rats administered GNT0004 when compared to vehicle (Figure 3A). DMD^{mdx} vehicle-treated rats had more foci of cardiac fibrosis in ventricular and septal areas, particularly in the papillary muscle, compared with WT + vehicle and WT + GNT0004 rats (corresponding to 10.5% ± 5.4%, 2.5% ± 1.9%, and 2.6% ± 1.6%, respectively) as determined by the percentage of surface occupied by connective tissue (marker of fibrosis). By comparison, the fibrosis levels of DMD^{mdx} were clearly reduced in cardiac muscle in DMD^{mdx} rats treated with 4.2×10^{14} and 2.1×10^{14} vg/kg of GNT0004 (4.9% ± 2.8% and 4.9% ± 3.4%, respectively) (Figures 3B and 3C).

In line with these results, at 6 months p.i., the levels of circulating creatine kinase (CK), aspartate aminotransferase (AST), alanine aminotransferase (ALT), and lactate dehydrogenase (LDH) were higher in vehicle-treated DMD^{mdx} rats than in vehicle-treated WT rats. These biomarkers are released from damaged skeletal muscles and the heart during the active phase of the disease in both patients with DMD and animal models.[42] These parameters were unaffected by GNT0004 treatment in WT rats. By contrast, they were significantly decreased in all GNT0004-treated DMD^{mdx} rats, reaching values comparable to those of vehicle-injected WT rats, regardless of the dose, confirming the therapeutic efficacy of GNT0004 at these doses (Figure 3D).

Additional serum chemistry markers (urea, creatinine, total proteins, and bilirubin) and hematological parameters (including red and white blood cell counts, hemoglobin, hematocrit, mean corpuscular volume [MCV], mean corpuscular hemoglobin [MCH], mean corpuscular hemoglobin concentration [MCHC], reticulocytes, neutrophils, lymphocytes, monocytes, eosinophils, basophils, and platelets) remained unchanged in both WT and DMD^{mdx} animals, regardless of the injected item or dose. These findings confirm an absence of GNT0004-related toxicity on these parameters.

GNT0004 delivery improved muscle function in DMD^{mdx} rats

Muscle function was evaluated in all animals at 3 and 6 months p.i. using the grip force test. Muscle fatigue was estimated by a decrease in muscle strength following five consecutive limb force tests separated by 30 s. As already described for the DMD^{mdx} rat model,[22,33,43] DMD^{mdx} + vehicle animals showed a progressive and significant decrease in forelimb strength when compared to WT + vehicle animals (Figure 4). Moreover, compared to the WT + vehicle group, the relative forelimb grip force in the DMD^{mdx} + vehicle group was reduced at 3 and 6 months p.i. (15% ± 7% and 17% ± 4% decrease in relative grip force, respectively, at 3 and 6 months p.i.; Figure S2).

Administration of GNT0004 at both 2.1×10^{14} and 4.2×10^{14} vg/kg had no impact on muscle strength or fatigue in WT animals at either 3 or 6 months after injection. In contrast, in DMD^{mdx} rats, relative forelimb grip force was improved and muscle fatigue was completely abolished 3 and 6 months after GNT0004 administration. The values measured in the treated DMD^{mdx} animals were similar to those obtained in WT controls, regardless of the injected dose (2.1×10^{14} or 4.2×10^{14} vg/kg) (Figures 4 and S2), demonstrating the therapeutic efficacy of GNT0004 in DMD^{mdx} rats.

GNT0004 partially improved cardiac function in DMD^{mdx} rats but also induced dose-dependent rhythm disorders, cardiac remodeling, and associated dysfunction in both WT and DMD^{mdx} rats

At 6 months p.i., cardiac function was assessed by electrocardiography, 2D echocardiography, and Doppler analysis on anesthetized animals.

loading control. (B) Quantification of hMD1 protein expression by Simple Western from total proteins extracted from *biceps femoris*, heart, and diaphragm of injected rats. Quantification was done respective to the level of total proteins and expressed as arbitrary units (a.u.). (C) Quantification of hMD1-positive muscle fibers after immunolabeling of *biceps femoris* and heart sections using the NCL-DYS3 antibody, which specifically recognizes the human dystrophin. (D) Immunohistochemical images of hMD1 expression in the *biceps femoris* and heart of injected rats. Representative results are presented for one animal of each experimental group. The NCL-DYS3 antibody was used to specifically detect hMD1 (red) expression, with DRAQ5 fluorescent DNA dye to detect nuclei (cyan). Scale bar: 50 μm. For (B) and (C), individual and mean values (±SEM) are shown. Statistical analyses performed using a nonparametric Kruskal-Wallis test followed by a post hoc Dunn's multiple comparisons test showed no significant difference between the different groups injected with GNT0004.

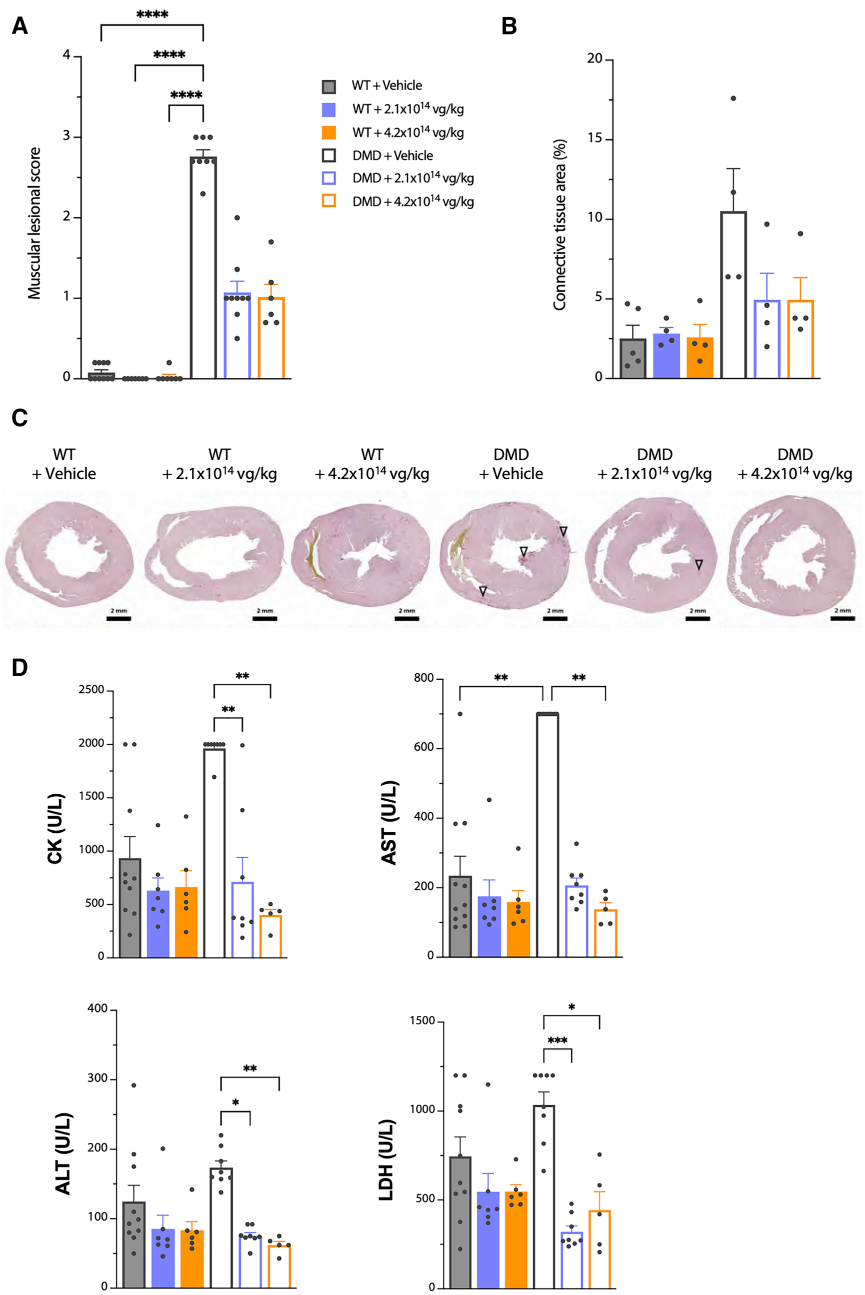

Figure 3. Muscle histopathology and circulating biomarkers values measured 6 months after vehicle or GNT0004 administration

Analyses were performed on samples obtained from animals still alive at 6 months p.i. (i.e., n = 6–10 per experimental group). (A) Semi-quantification of histopathological lesions evaluated in skeletal muscles (including diaphragm) and cardiac muscle. (B) Assessment of fibrosis in the heart. (C) Transverse heart sections stained with picrosirius red to visualize connective tissue (purple). Foci of ventricular fibrosis were sometimes observed (arrowhead). (D) Circulating creatine kinase (CK), aspartate aminotransferase (AST), aspartate aminotransferase (ALT), and lactate dehydrogenase (LDH) values. For (A), (B), and (D), individual and mean values (±SEM) are shown. Statistical analyses were performed using a nonparametric Kruskal-Wallis test followed by a post hoc Dunn's multiple comparisons test (****$p < 0.0001$, ***$p < 0.001$, **$p < 0.01$, and *$p < 0.05$).

ECG data, presented in Figure 5A, show a non-significant change in heart rates and PR intervals, independent of the experimental group. On the contrary, an increase in mean QT and QTc (QT corrected) intervals was observed in vehicle-treated DMD^{mdx} rats compared with their WT counterparts, although this effect was not statistically significant due to the high intra-group variability. Injection of 2.1 × 10^{14} vg/kg GNT0004 in WT or DMD^{mdx} rats did not significantly modify QT or QTc with respect to their vehicle-treated counterparts. Conversely, a significant increase in the QT interval was observed for the WT + 4.2 × 10^{14} vg/kg group compared to the vehicle-treated WT group (also presented in Figure S3). This significant effect persisted after normalization of this parameter to heart frequency (QTc). A significant increase in QTc was also observed in DMD^{mdx} + 4.2 × 10^{14} vg/kg animals as compared to WT + vehicle rats but not in DMD^{mdx} + vehicle rats. This observation ggests that high-dose GNT0004 (4.2 × 10^{14} vg/kg) may increase QT and therefore constitutes a potential source of arrhythmia in healthy WT rats but does not further prolong QT/QTc intervals in DMD^{mdx} rats.

As already described for the DMD^{mdx} rat model,[22,33,43] echocardiography analyses revealed a slight increase in left ventricular anterior wall thickness in vehicle-treated DMD^{mdx} animals and a slight decrease in left ventricular diameter when compared to vehicle-treated WT animals (Figure 5B). After GNT0004 administration, a trend to normalization of wall thickness was observed in the DMD^{mdx} animals injected with the 4.2 × 10^{14} vg/kg dose, whereas no modification was observed in the DMD^{mdx} animals injected with the 2.1 × 10^{14} vg/kg dose, nor in WT animals treated with either dose. However, compared to their vehicle-treated counterparts, there was a dose-related trend toward a decrease in left ventricular diameter in WT animals injected with 4.2 × 10^{14} vg/kg, as well as in DMD^{mdx} animals injected with both doses (2.1 × 10^{14} and 4.2 × 10^{14} vg/kg). These differences did not reach statistical significance owing to intra-group variability. Nevertheless, this trend could suggest that GNT0004 treatment induces concentric remodeling processes in WT and DMD^{mdx} animals that may be worsened with higher doses. In line with this structural remodeling, we observed an

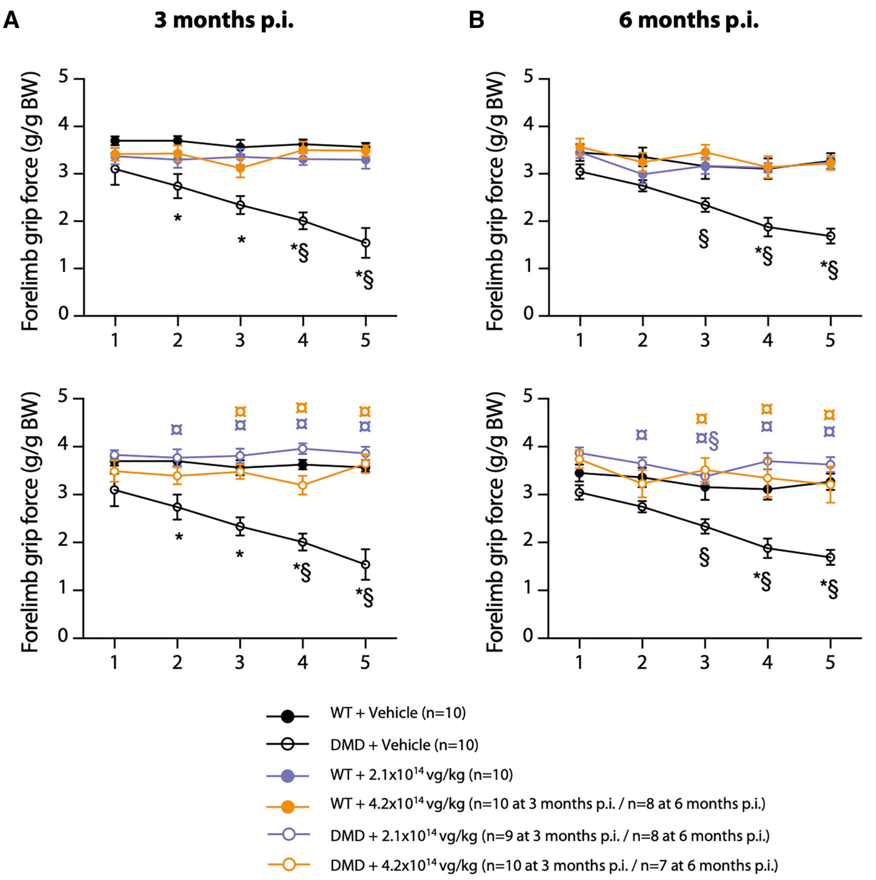

Figure 4. Grip test assessment of skeletal muscle function and fatigue after vehicle or GNT0004 administration

Analyses were performed at 3 (A) and 6 (B) months p.i. on animals still alive at the time of analysis (i.e., n = 7–10 per experimental group). Data plots show the mean forelimb grip force (g/g normalized to body weight [BW]) ± SEM collected over five successive grip test trials. Statistical analyses were performed using a nonparametric Kruskal-Wallis test followed by a post hoc Dunn's multiple comparisons test (*$p \leq 0.05$ vs. corresponding WT group [treated with vehicle or GNT0004] and ¤$p \leq 0.05$ vs. corresponding vehicle-treated group [WT or DMD^{mdx}]). Cross-trial evolution of force was analyzed using the Friedman test followed by Dunn's post-hoc test (§$p \leq 0.05$ vs. trial 1 for the same group).

increase in the ejection fraction in GNT0004-treated animals, regardless of dose (2.1×10^{14} or 4.2×10^{14} vg/kg) or genotype (WT or DMD^{mdx}). This increase was statistically significant in GNT0004-treated DMD^{mdx} rats compared to vehicle-treated DMD^{mdx} rats. This phenomenon may reflect a functional adaptation (compensation) to counteract the defects induced by the structural remodeling.

Doppler analysis confirmed the diastolic dysfunction, previously described in the DMD^{mdx} rat model,[22,33,43] with a significantly increased isovolumetric relaxation time (IVRT) and deceleration time (DT) and a significantly decreased E/A ratio when compared to the WT + vehicle group. At 6 months p.i., the diastolic dysfunction was improved in DMD^{mdx} animals treated with GNT0004, with normalization of IVRT and DT values and partial correction of E/A ratio values, with no dose effect. In WT animals, no alterations in IVRT values were observed after any GNT0004 treatment. Conversely, injection of GNT0004 resulted in a dose-dependent trend toward a decreased E/A mean ratio in WT animals and increased mean DT, even if the observed differences did not reach statistical differences.

Altogether, these analyses, performed at 6 months p.i., show that administration of GNT0004 improves diastolic dysfunction in DMD^{mdx} rats. However, the 4.2×10^{14} vg/kg dose may simultaneously induce pathological concentric cardiac remodeling, reflected by a reduced left ventricular diameter and emerging systolic dysfunction. In WT animals, similar structural and functional abnormalities (including diastolic dysfunction) were observed at both tested doses (2.1×10^{14} and 4.2×10^{14} vg/kg), potentially driven by arrhythmic events, such as prolonged QT/QTc intervals.

GNT0004 increased arrhythmic events and sudden death, predominantly in WT rats

To explore putative rhythm disorders, ECG in conscious animals was recorded by telemetry for 22-h periods once a week from 1 month p.i. up to 6 months p.i. Measurements were collected during the first 3 min of each hour of recording, and a decision tree was generated to empirically classify arrhythmic events into three categories. Minimal change in ECG tracing, i.e., "ripple type" or "burst type" events of variable intensity that were not associated with the animal's movements (which is a potential source of artifacts), were empirically considered as "low-impact events" that did not represent cardiac electrophysiology impairment. Ventricular extrasystoles were considered "medium-impact events" if they did not alter the sinus rhythm. Very frequent early ventricular beats (EVBs) were considered a risk factor for arrhythmia-induced cardiomyopathy, which is characterized by a decreased efficiency of cardiac muscle and the appearance of heart failure symptoms and an association with an increased risk of sudden cardiac death. Some of these extrasystoles can alter heart rate, resulting in more severe consequences. In this study, these former events were classified as "high-impact events." This category also included polymorphic extrasystole, tachycardia, and pauses (i.e., temporary stops of sinus activity). Both medium- and high-impact events were indicative of cardiac electrophysiological dysfunction. A color-coded schematic representation of the results obtained in each animal is presented in Figure S4. All of the animals included in the study displayed rhythm disorder events regardless of genotype (DMD^{mdx} or WT) or treatment (vehicle or GNT0004 at 2.1×10^{14} or

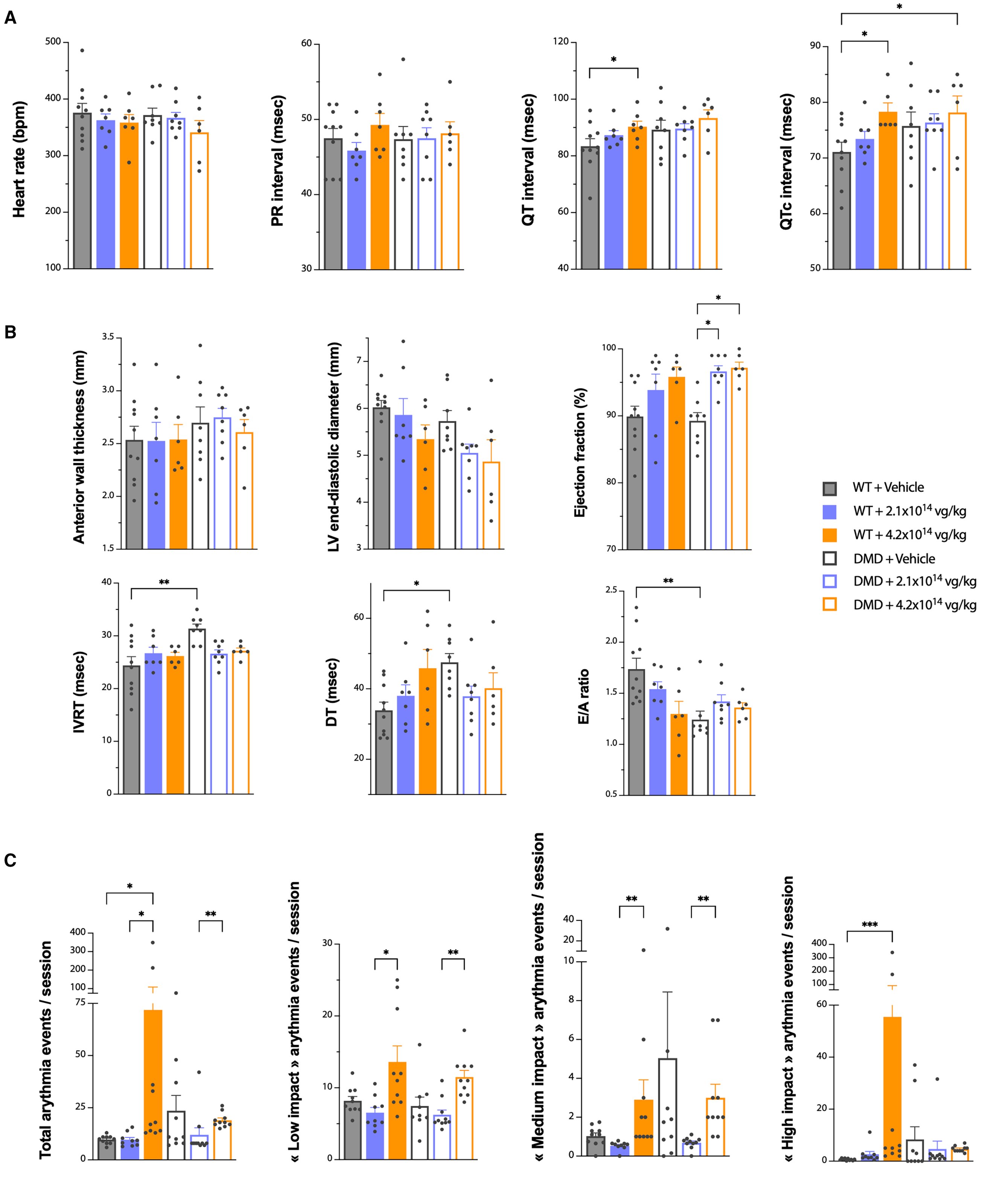

(legend on next page)

4.2×10^{14} vg/kg) and irrespective of the circadian cycle or the follow-up duration. The heatmap suggests that DMD^{mdx} rats (whether vehicle or GNT0004 treated) exhibited a higher risk of abnormal ECG events and death compared to vehicle-treated WT rats. GNT0004-related increases in impact events were observed only in WT animals. Of note, four rats died during the telemetry recording sessions. The cause of death of three of these animals was underdetermined by histopathology: one WT + 2.1×10^{14} vg/kg animal was deceased at day 113 p.i., one WT + 4.2×10^{14} vg/kg animal was deceased at day 180 p.i., and one DMD^{mdx} + 4.2×10^{14} vg/kg animal was deceased at day 157 p.i. (see Table S2 and Figure S4). Schematic representations of the ECG telemetric tracking results obtained from these particular animals, including at the time of death, are presented in Figures S5–S7. Despite the absence of major events in previous recordings, these animals presented major rhythm disorders in the hours and minutes before sudden death with final "torsade de pointes."

For a more comprehensive interpretation of these analyses, a quantitative evaluation of rhythm disorder events measured per session (i.e., each week) for each experimental group and per type of event (total and low, medium, or high impact) is presented in Figure 5C. Despite intra-group variability, vehicle-treated DMD^{mdx} animals presented a higher number of total, medium-, and high-impact events compared to WT rats, suggesting a disease-induced arrhythmogenic phenotype. Injection of 2.1×10^{14} vg/kg of GNT0004 in DMD^{mdx} rats resulted in a normalization in the number of these rhythm disorder events. However, a significant increase in the number of total, low-, and medium-impact events in the 4.2×10^{14} vg/kg DMD^{mdx} group was compared to the 2.1×10^{14} vg/kg DMD^{mdx} group. In WT rats, administration of 2.1×10^{14} vg/kg of GNT0004 induced only a mild increase in high-impact-event frequency. On the contrary and despite the variability observed within this group, significantly higher amounts of arrhythmic events were seen in the 4.2×10^{14} vg/kg WT group when compared to WT animals injected with vehicle or 2.1×10^{14} vg/kg of GNT0004.

The ECG data did not allow for a strict link between the type of rhythm disorder events and rat survival at the individual level. This could be due to the ECG recordings being done over limited periods. Nevertheless, such frequent arrhythmic events clearly constitute a major risk of sudden death in animals, especially in WT animals treated with GNT0004 at the 4.2×10^{14} vg/kg dose. In DMD^{mdx} animals, we observed a normalization of the number of arrhythmic events at the 2.1×10^{14} vg/kg dose. At the 4.2×10^{14} vg/kg dose, the return of arrhythmic event incidence to levels observed in vehicle-treated DMD^{mdx} animals suggests that this high dose may also exacerbate arrhythmic risk in the DMD context.

GNT0004 administration induced a reduction of endogenous dystrophin protein expression in WT rats

In order to understand which process could be at the origin of the cardiac dysfunction that occurred in some animals after GNT0004 administration, endogenous rDys protein expression was analyzed in a portion of the animals 6 months p.i.

A semi-quantitative western blot analysis of endogenous rDys protein expression levels was performed on the *biceps femoris*, diaphragm, and heart (Figure 6A). As expected, in all tissues from DMD^{mdx} rats and regardless of treatment (GNT0004 or vehicle), no endogenous rDys expression was detected, while the protein was observed in all muscles of the WT animals. Surprisingly, the expression of endogenous rDys was dose-dependently reduced upon GNT0004 administration in WT animals. In heart samples, this reduction reached 3- and 6-fold factors at the 2.1×10^{14} and 4.2×10^{14} vg/kg doses, respectively.

In parallel, sections from frozen *biceps femoris* and heart samples were immunolabeled using the NCL-DYS2 anti-dystrophin antibody. NCL-DYS2 specifically recognizes the C-terminal (C-term) domain of native dystrophin from different species. Because the hMD1 protein, encoded by GNT0004, does not contain the C-term domain of dystrophin, the NCL-DYS2 antibody allows for the detection of only the endogenous dystrophin in the rats, not the transgene. The results presented in Figure 6B show the percentage of NCL-DYS2-positive fibers calculated in the *biceps femoris* and cardiac muscle samples. In cardiac and skeletal muscle sampled from WT + vehicle animals, all muscle fibers displayed intense, homogeneous subsarcolemmal labeling of endogenous dystrophin. On the contrary, samples obtained from DMD^{mdx} + vehicle rats showed a subsarcolemmal NCL-DYS2 labeling only in a small percentage of revertant fibers scattered throughout the sample (in 0.1%–11.1% of fibers). These revertant fibers were previously described in patients with DMD and in DMD animal models, including DMD^{mdx} rats.[44,45] After injection of GNT0004, no modification of these

Figure 5. Assessment of hMD1 expression on cardiac function
Analyses were performed on animals still alive at the time of analysis (i.e., $n = 6$–10 per experimental group). (A) Electrocardiographic evaluation at 6 months after vehicle or GNT0004 administration. Heart rate, PR interval, QT interval, and QTc interval (QT interval corrected with heart rate) were measured. (B) Echocardiography and pulsed Doppler evaluation at 6 months after vehicle or GNT0004 administration. For structural parameters, left ventricle (LV) anterior wall thickness and diameter were measured during diastole from long-axis images obtained by M-mode echocardiogram. For diastolic function, isovolumetric relaxation time (IVRT), deceleration time (DT), and E/A ratio were measured using pulsed Doppler with an apical 4-chamber orientation. For systolic function, ejection fraction was also measured during diastole from long-axis images obtained by M-mode echocardiogram. (C) Quantitative analyses of ECG results obtained using telemetry. The mean number of rhythm disorder events measured per session (i.e., each week) was evaluated for each animal. The results of each experimental group were compared, considering the total number of events or the different types of events; potentially low-, medium-, and high-impact events. Individual and mean values (±SEM) are shown. Statistical analyses were performed using a nonparametric Kruskal-Wallis test followed by a post hoc Dunn's multiple comparisons test (***$p < 0.005$, **$p < 0.01$, and *$p < 0.05$).

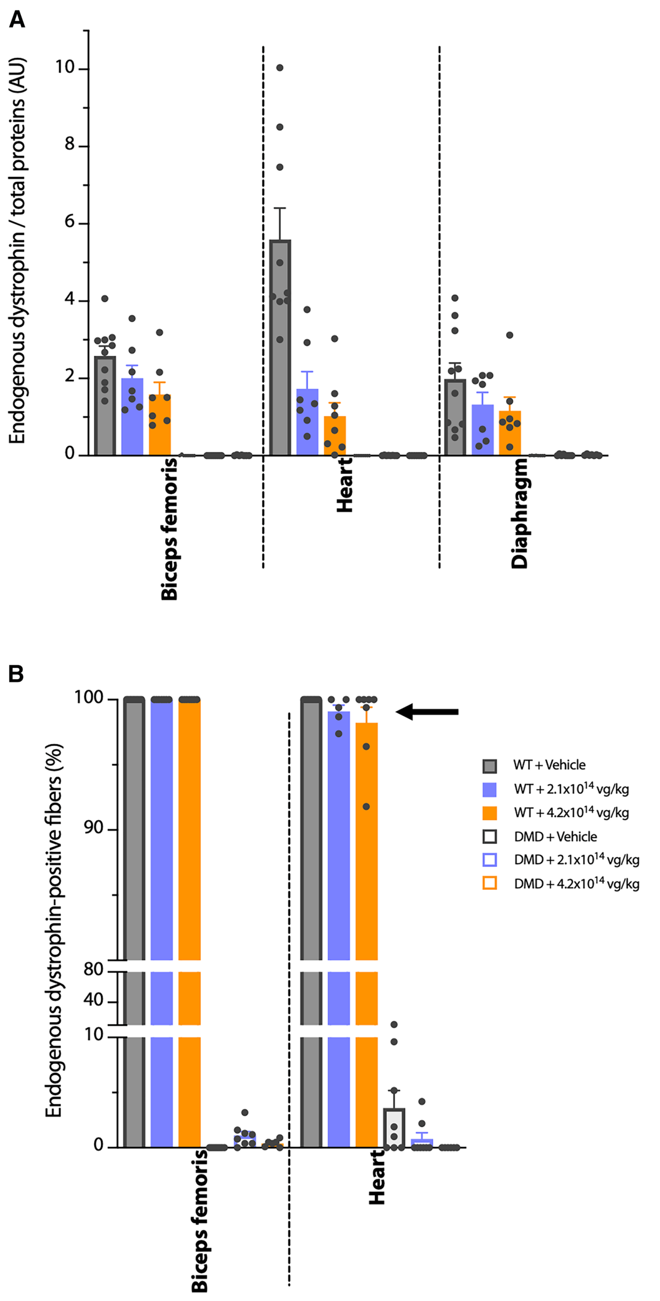

Figure 6. Quantification of endogenous rat dystrophin levels in WT and DMD^{mdx} rat muscles 6 months after administration of vehicle or GNT0004

Analyses were performed on samples obtained from animals still alive at 6 months p.i. (i.e., *n* = 6–10 per experimental group). (A) Quantification of endogenous rat dystrophin protein expression levels by western blot from total proteins extracted from *biceps femoris*, heart, and diaphragm of injected rats. Each blot was stained with MANEX-1011C to detect the presence of the 427-kDa dystrophin protein. Quantification was done respective to the level of total proteins and expressed as arbitrary units (a.u.). (B) Quantification of hMD1-positive muscle fibers after immunolabeling in *biceps femoris* and heart sections using the NCL-DYS2 antibody, which specifically recognizes the endogenous rat dystrophin. The black arrow shows the differences observed in the heart samples between the different groups of WT animals. Individual and mean values (±SEM) are shown. Statistical analyses, performed using a nonparametric Kruskal-Wallis test followed by a post hoc Dunn's multiple comparisons test, showed no significant differences between the different groups.

patterns was observed in the *biceps femoris* samples, whatever the genotype or injected dose. On the contrary, in heart samples from GNT0004-treated DMD^{mdx} rats, the number of NCL-DYS2-positive revertant fibers was inversely correlated with the dose of GNT0004 and the level of hMD1 expression (see Table S4). Additionally, six heart samples obtained from GNT0004-treated WT rats (3 WT + 2.1 × 10^{14} vg/kg and 3 WT + 4.2 × 10^{14} vg/kg) displayed lower numbers of endogenous rDys-positive fibers in the heart (97%–99% with 2.1 × 10^{14} vg/kg and 91%–99% 4.2 × 10^{14} vg/kg dose GNT0004 vs. 100% vehicle). This observation was associated with a generalized higher level of hMD1 expression in the same samples (90%–100% of DYS3-positive fibers). This was also seen in two WT + 4.2 × 10^{14} vg/kg rats that died prematurely but were still exploitable for immunohistochemistry studies. In the hearts of these animals, only 82.7% and 86.4% of native dystrophin-positive fibers were detected, while a high number of fibers expressing hMD1 were observed (93.7% and 95.9% of NCL-DYS3+ positive fibers, respectively). In one of these animals, a dramatic decrease in native dystrophin-positive fibers was also observed in the biceps femoris (30.6% of endogenous dystrophin-positive fibers), while 100% of the fibers expressed the transgene hMD1.

These results indicate that in the *biceps femoris* muscle, diaphragm, and heart, endogenous rDys expression is dose-dependently down-regulated in the muscles of GNT0004-treated WT animals, with a particularly dramatic reduction observed in the heart. This suggests a regulation and/or destabilization of endogenous dystrophin expression upon GNT0004 treatment and subsequent hMD1 expression.

High-dose GNT0004 administration induced remodeling of the DAPC in both the skeletal muscles and heart of WT rats

The consequences of GNT0004 administration on utrophin (the functional autosomal paralog of dystrophin) and on DAPC components were evaluated. For the animals 6 months p.i., immunostaining and Simple Western analyses showed no difference in utrophin expression in the skeletal and cardiac muscles of vehicle- and GNT0004-treated WT rats (Figures S8–S10). Compared to WT + vehicle rats, utrophin was overexpressed in DMD^{mdx} + vehicle rats, a phenomenon that has already been described as a compensatory mechanism of dystrophin deficiency.[46,47] Normal utrophin expression was restored at both tested doses in the GNT0004-treated DMD^{mdx} rats.

Serial transverse sections of heart and *biceps femoris* muscle obtained at 6 months p.i. were immunolabeled using antibodies for α-sarcoglycan, β-dystroglycan, and γ-sarcoglycan to analyze the DAPC, along with native rDys and hMD1. In-group findings were homogenous, with similar findings obtained for all muscle types analyzed. Representative results obtained in heart samples are presented in

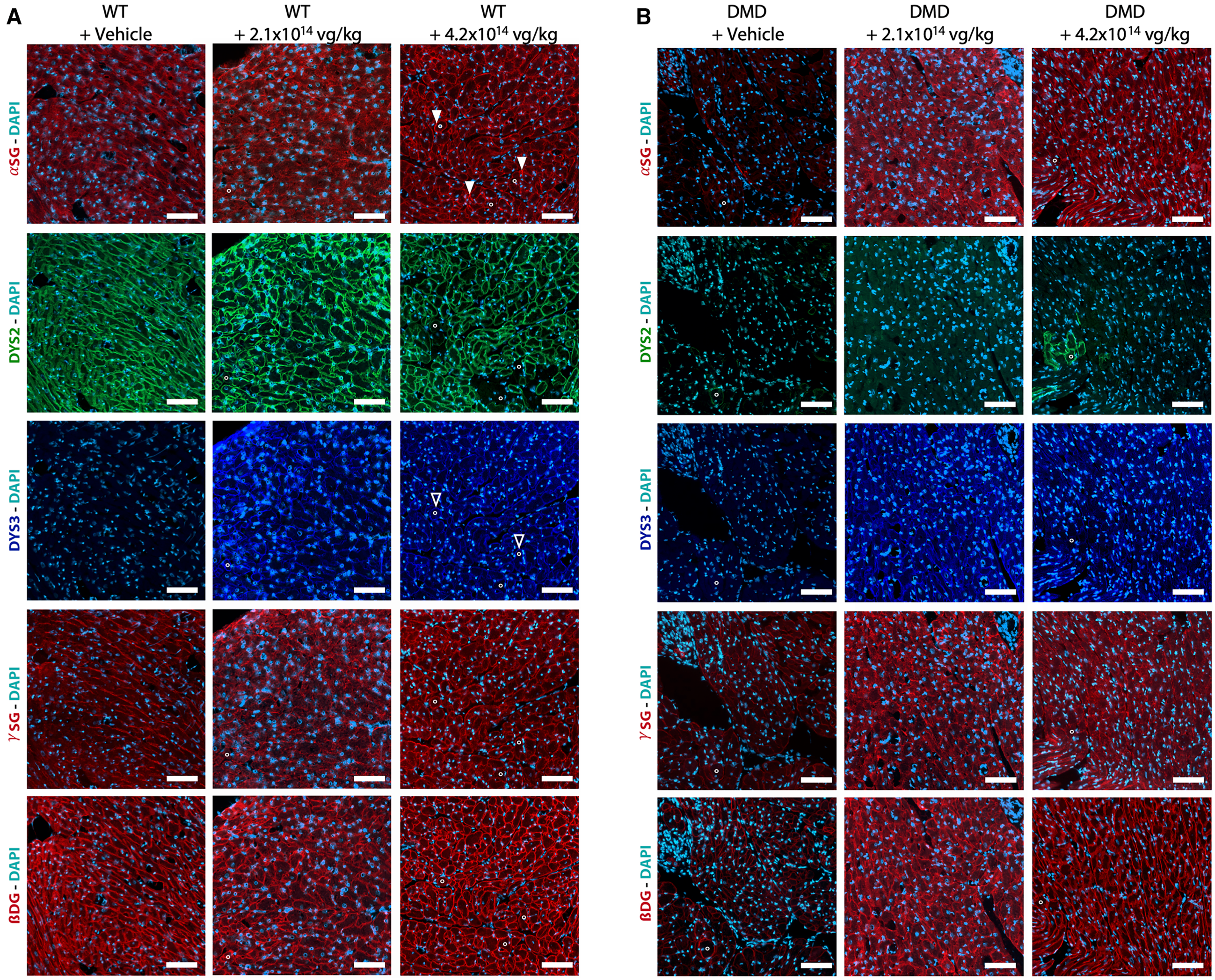

Figure 7. DAPC components in rat heart samples at 6 months after administration of vehicle or GNT0004

Analyses were performed on samples obtained from WT (A) and DMD^{mdx} (B) rats that were still alive at 6 months p.i. (i.e., n = 6–10 per experimental group). Representative images of immunolabeling with antibodies against α-sarcoglycan (α-SG), rat native dystrophin (DYS2), hMD1 (DYS3), γ-sarcoglycan (γ-SG), and β-dystroglycan (β-DG). Compared to vehicle-injected WT rats, the WT rats injected with 4.2×10^{14} vg/kg of GNT0004 displayed subsarcolemmal clusters of α-SG (arrowhead) in fibers with low residual rat dystrophin expression but with high levels of hMD1 expression (open arrowhead). Fibers of interest were identified (°) in serial sections. Scale bar: 50 μm.

Figure 7. As expected, the expression of α-sarcoglycan, β-dystroglycan, and γ-sarcoglycan in rats from the DMD^{mdx} + vehicle group was very low, except in revertant fibers. By contrast, all rats from the DMD^{mdx} + GNT004 groups displayed a restored normal expression pattern of the DAPC proteins analyzed. In WT + vehicle rats, continuous, homogeneous subsarcolemmal labeling for α-sarcoglycan, β-dystroglycan, γ-sarcoglycan, and rat native dystrophin was observed. WT rats treated with 4.2×10^{14} vg/kg of GNT0004 displayed an unchanged homogeneous and continuous pattern of β-dystroglycan and γ-sarcoglycan expression along the cardiac fibers. By contrast, α-sarcoglycan labeling was more heterogeneous, with dense multifocal deposits observed along the sarcolemma in fibers with low residual rDys expression but with high levels of hMD1 expression. No such abnormalities were seen in the WT rats treated with 2.1×10^{14} vg/kg of GNT0004.

WT animals were more sensitive to hMD1 overexpression, a cause of sudden premature death

To conclude the possible causes of premature death of some animals before the end of the study, the observations made on each of these animals (including clinical observations, ECG, histopathology [as described in Table S2], and, when available, levels of endogenous native dystrophin expression vs. hMD1 expression) were combined in Table 1.

Table 1. Summary of premature deaths that occurred during the study and related observations for each animal

Experimental group	Time and condition of death	Comments on previous conditions	Cause of death determined by histopathology (see Table S2)	Quantitative rDys/hMD1 expression	Death related to GNT0004 product?
WT + 2.1 × 10^{14} vg/kg	D3 p.i.; found dead in its housing cage	nothing to report; no ECG follow-up availabl	coagulation defect	not available	cannot be ruled out
	D78 p.i.; found dead in its housing cage	nothing to report that differs from vehicle-treated WT rats	inconclusive (autolysis of the tissues)	not available	cannot be ruled out
	D113 p.i.; found dead in its telemetry cage	major rhythm disorders and final torsade de pointes observed just before death (see Figure S4)	chronic progressive nephropathy	not available	cannot be ruled out
	D137 p.i.; cardiorespiratory arrest a few minutes after telemetry session	numerous potential high-impact arrhythmia events observed in the weeks before death (see Figure S3)	no identified cause of death	not available	cannot be ruled out
WT + 4.2 × 10^{14} vg/kg	D96; cardiorespiratory arrest during preparation for telemetry session	signs of stress[a] and reduced weight gain from 1 month p.i.; numerous potential high-impact arrhythmia events observed in the weeks before death (see Figure S3)	no identified cause of death	decreased levels of rDys-positive fibers in the heart (86.4%) associated with high levels of hMD1 expression (95.9%)	yes; decreased rDYS expression, probable destabilization of the DAPC, which may promote onset of a lethal arrhythmic event
	D105 p.i.; found dead in its housing cage	nothing to report	inconclusive (autolysis of the tissues)	not available	cannot be ruled out
	D180 p.i.; found dead in its telemetry cage	signs of stress[a] and reduced weight gain from 1.5 months p.i.; major rhythm disorders and final torsade de pointes observed just before death (see Figure S5)	no identified cause of death	decreased levels of rDys-positive fibers in the heart (82.7%) associated with high levels of hMD1 expression (93.7%); dramatically decreased levels of rDys-positive fibers in *biceps femoris* (only 30.6% of positive fibers) associated with a generalized expression of hMD1 (100%)	yes; decreased rDYS expression, probable destabilization of the DAPC, which may promote onset of a lethal torsade de pointes
DMD^{mdx} + vehicle	D29 p.i.; cardiorespiratory arrest during preparation for telemetry session	nothing to report; no ECG follow-up available	malignant hyperthermia-like syndrome + cardiac failure	not available	no (death linked to DMD pathology)
	D54 p.i.; cardiorespiratory arrest during preparation for telemetry session	nothing to report	malignant hyperthermia-like syndrome	not available	no (death linked to DMD pathology)
	D71 p.i.; cardiorespiratory arrest during preparation for telemetry session	nothing to report	malignant hyperthermia-like syndrome	not available	no (death linked to DMD pathology)
DMD^{mdx} + 2.1 × 10^{14} vg/kg	D60 p.i.; found dead in its telemetry cage	excessive bradycardia and decompensation observed just before the death (see Figure S6)	cardiac failure	not available	no (death linked to DMD pathology)

(*Continued on next page*)

Table 1. *Continued*

Experimental group	Time and condition of death	Comments on previous conditions	Cause of death determined by histopathology (see Table S2)	Quantitative rDys/ hMD1 expression	Death related to GNT0004 product?
	D107 p.i.; found dead in its housing cage	nothing to report that differs from vehicle-treated DMD^{mdx} rats	malignant hyperthermia-like syndrome	not available	no (death linked to DMD pathology)
DMD^{mdx} + 4.2 × 10^{14} vg/kg	D107 p.i.; found dead in its housing cage	nothing to report that differs from vehicle-treated DMD^{mdx} rats	malignant hyperthermia-like syndrome	not available	no (death linked to DMD pathology)
	D142 p.i.; found dead in its housing cage	nothing to report that differs from vehicle-treated DMD^{mdx} rats	no identified cause of death	not available	cannot be ruled out
	D157 p.i.; found dead in its telemetry cage	major rhythm disorders and final torsade de pointes observed just before death (see Figure S7)	no identified cause of death	not available	cannot be ruled out
	D174 p.i.; found dead in its housing cage	signs of stress[a] from 1.5 months p.i.	chronic progressive nephropathy	not available	no (lesions commonly observed in Sprague-Dawley rats)

rDys, rat endogenous dystrophin; D, day; p.i., post-injection.
[a]Including one or several of the following signs: chromodacryorrhea, passivity, alopecia, piloerection, dyspnea, vocalization, dehydration, and/or sensitivity to handling.

As previously described, histopathological analyses performed on tissues obtained after death indicated that the cause of death differed between WT and DMD^{mdx} animals.

In DMD^{mdx} rats, most deaths were demonstrated to be unrelated to the GNT0004 treatment but were instead linked to the DMD pathology itself or to non-specific lesions commonly observed in Sprague-Dawley rats. These findings suggest that the two deaths observed following the administration of 2.1 × 10^{14} vg/kg GNT0004 in DMD^{mdx} animals were likely linked to disease progression rather than to the treatment. However, for the two deaths that occurred in DMD^{mdx} rats after administration of the 4.2 × 10^{14} vg/kg dose, a contribution of GNT0004 cannot be excluded, as no definitive cause could be established.

A link with the GNT0004 product cannot be ruled out for one of the premature deaths in the WT animals injected with the 4.2 × 10^{14} vg/kg dose but was clearly established for the two other deaths. In these two animals, cumulative hMD1 protein expression coincided with a GNT0004 dose-related reduction of the endogenous dystrophin in muscle tissues and, more dramatically, in the heart, pointing to a potential competition between hMD1 and the endogenous dystrophin protein. A correlation between the premature deaths and the GNT0004 product at the 2.1 × 10^{14} vg/kg dose in WT animals cannot be ruled out for the four relevant animals, two of which presented major ECG disorders before death. One death, which had large hemorrhages, was observed at 3 days p.i. in one rat from this group and could possibly be associated with a coagulation defect.

In summary, these results suggest that a single i.v. injection of the highest tested dose of 4.2 × 10^{14} vg/kg GNT0004 can induce mortality in WT rats, and to a lesser extent in DMD^{mdx} rats, within several weeks of GNT0004 injection (mainly from 3 months p.i.). GNT0004-related mortality could also be observed in some WT rats (but not in DMD^{mdx} rats) injected with 2.1 × 10^{14} vg/kg of GNT0004, suggesting a greater toxicity of GNT0004 in WT animals.

DISCUSSION

The objective of the present study was to determine the safety of the GNT0004 product, an rAAV8-hMD1 vector designed for the treatment of DMD, and to further characterize and understand the observed cardiac toxicity after the injection of high doses of this product in WT and DMD^{mdx} rats.

Administration of GNT0004 at 2.1 × 10^{14} and 4.2 × 10^{14} vg/kg doses was associated with a marked induction of hMD1 in both WT and DMD^{mdx} rats. It induced striking beneficial effects in DMD^{mdx} rat skeletal muscles and hearts at the histological and functional levels, including the correction of circulating biomarker levels, grip strength, and diastolic function, the impairment of which is a hallmark of DMD.

In parallel, several premature death events were observed within this study. Confounding deaths were either related to stress-induced malignant hyperthermia-like syndrome (as described in patients with DMD and in DMD^{mdx} rats[35,36]); chronic nephropathy, known to occur in Sprague-Dawley rats[34]; an early incidental case at 3 days p.i. due to coagulation abnormalities; or one disease-related decompensation process in a DMD^{mdx} rat. The remaining deaths (5/7 in WT animals and 2/9 in DMD^{mdx} animals) could not be explained by histopathological analyses.

ECG telemetry performed during the 6 months p.i. and classical ECG analyses performed at 6 months p.i. showed that administration of GNT0004 in WT animals at a 4.2×10^{14} vg/kg dose promotes arrhythmic events and an increase in QT/QTc intervals that could lead to sudden death. At the 2.1×10^{14} vg/kg dose, even if the mean number of arrhythmic events was similar to that observed in the WT + vehicle group, a sudden death after a severe arrhythmic episode was still observed in one animal. This suggests that the injection of 2.1×10^{14} vg/kg GNT0004 can also be detrimental in the WT context. The arrhythmic events observed in these animals could contribute to structural (concentric remodeling) and functional (onset of systolic and diastolic dysfunctions) pathological adaptations as observed at 6 months p.i. using 2D echocardiography/pulsed Doppler analyses and could constitute a major risk of sudden death.

In DMD^{mdx} rats, the effect of GNT0004 is difficult to distinguish from the normal evolution of the DMD pathological status. Indeed, high-impact arrhythmic events, such as ventricular extrasystoles, may occur spontaneously in untreated DMD^{mdx} rats and are possibly related to the abnormal structure of cardiac muscle with fibrosis, inflammation, and/or necrosis.[48–50] It was not possible to accurately establish a link between the frequency/presence of rhythm disorder events and survival in DMD^{mdx} rats, as some animals died prematurely due to major rhythm disorders, despite the absence of any detectable prior severe events.

Regardless of the injected dose, GNT0004 did not alter QT/QTc intervals in DMD^{mdx} rats and improved diastolic dysfunction compared to vehicle-treated counterparts. At the 2.1×10^{14} vg/kg dose, the number of arrhythmic events was normalized. In contrast, at the 4.2×10^{14} vg/kg dose, the arrhythmic event incidence returned to levels observed in vehicle-treated DMD^{mdx} rats, suggesting that this higher dose may exacerbate arrhythmic risk in the DMD context. Combined with evidence that GNT0004 at a 4.2×10^{14} vg/kg dose induced pathological cardiac remodeling and systolic dysfunction, the possibility that some of the sudden deaths observed in this group were related to the overdosed treatment cannot be excluded.

In terms of gene transfer efficacy, no differences were seen in the amounts of VCN between WT and DMD^{mdx} rats in any of the skeletal muscles, heart, liver, and blood tissues analyzed, indicating a similar biodistribution of GNT0004, regardless of the injected dose and the genotype of the animals. Administration of 2.1×10^{14} or 4.2×10^{14} vg/kg GNT0004 induces high and saturating levels of hMD1 protein expression in skeletal muscles and hearts of both WT and DMD^{mdx} rats. This apparent plateau in transgene expression may reflect a saturation of rAAV vector expression capacity at these high doses. This is a phenomenon that has already been described. Nakai et al.[51] reported a plateau in rAAV-mediated liver transduction in mice beyond a dose of approximately 1×10^{14} vg/kg, suggesting saturation of vector uptake or intracellular processing mechanisms. In line with this, concerns about diminished efficacy and increased toxicity at doses exceeding 1×10^{14} vg/kg were also raised during recent FDA advisory meetings, where Angela Lek et al. highlighted similar dose-plateau effects in different clinical and preclinical studies.[52] The findings presented in the current study are consistent with these publications in showing that a dose beyond a certain threshold does not necessarily improve transgene expression and may instead introduce more variability or adverse effects.

In GNT0004-treated WT rats, the level of hMD1 protein expression coincides with a dose-related reduction of the endogenous dystrophin in skeletal muscles and heart, pointing to a potential competition between the exogenous hMD1 and the endogenous rDys. This, in turn, may lead to DAPC destabilization, based on the observed concomitant abnormal distribution of α-sarcoglycan, one of the components of the DAPC. The consecutive impairment of some of the DAPC functions could include a perturbed structural link between laminin-2 in the extracellular matrix and F-actin of the cytoskeleton.[53,54] Patients with mutations associated with members of the DAPC may or may not develop cardiomyopathy,[55] in some cases because of compensatory mechanisms.[56] Based on the presented data, it can be speculated that (1) in the context of a normal tissue (with 100% endogenous dystrophin) that evolved with a normal, fully functional network of submembrane and transmembrane proteins (that includes dystrophin and components of the DAPC), the strong hMD1 expression feature perturbs the normal arrangement of the dystrophin and the DAPC network by entering into competition with the endogenous dystrophin; (2) this, in turn, may negatively impact the interactions of the endogenous dystrophin and proteins of the DAPC with other important proteins, such as ion channels involved in cardiomyocyte excitability and repolarization,[57] which, together with the fibrotic remodeling, may explain the increased number of arrhythmic events and therefore a dose-related increased risk of sudden death. Further investigations are warranted to understand the underlying link to the disturbance of the DAPC with the imbalance between hMD1 and endogenous dystrophin.

In DMD^{mdx} animals, native dystrophin is only expressed at very low levels, in less than 5% of the so-called revertant muscle fibers. The functional consequences of the revertant fibers are unclear.[45,58] Due to the low level of native dystrophin, the repercussions of the competition between hMD1 and native dystrophin isoforms in DMD^{mdx} rats are likely limited. However, although the administration of GNT0004 exerted therapeutic effects in a DMD context, rats from the DMD^{mdx} group treated with the high dose of 4.2×10^{14} vg/kg GNT0004 still presented an increased arrhythmic risk and cardiac pathological remodeling, as well as reduced gain of BW. Therefore, the dose of 4.2×10^{14} vg/kg should also be considered potentially toxic in the DMD^{mdx} rats. One could hypothesize that the overexpression of MD may disturb some specific compensatory mechanisms for the absence of full-length dystrophin in DMD^{mdx} rats. Interestingly, a link between a potential overexpression of MD and arrhythmia was observed in the *mdx* mouse model using a transgenic approach. Transgenic *mdx* mice that selectively expressed a minidystrophin gene in the heart at 100-fold of the normal levels showed worsening of several ECG parameters.[59] These

coincidental observations highlight the interest of investigating further the pathophysiological mechanisms of cardiomyopathy and the evaluations of DMD gene therapies. A more recent communication by Hart et al.[60] suggests that the treatment of D2.*mdx* mice with a clinical dose (i.e., 2×10^{14} vg/kg) of AAV10-MDs leads to MD-induced cardiomyopathy and a concomitant decrease in utrophin expression. Strikingly, in Hart et al.'s study, rAAV-treated mice showed a shorter mean life span compared to untreated D2.*mdx* animals, suggesting a real toxicity of the evaluated MDs. In the current study, no deleterious impact on utrophin expression was observed. Utrophin expression was decreased in treated DMD^{mdx} rats. This effect is consistent with a beneficial impact of MD expression in DMD animals, as utrophin is known to be upregulated in dystrophin-deficient muscle.[46,47] No modifications of utrophin expression levels were observed in WT animals. Discrepancies between the presented results and the ones obtained by Hart et al.[60] may be due to several factors, including different animal models, AAV serotype, manufacturing process, muscle-specific promoters, doses, MD protein overexpression, and time points of analysis (12–18 months of follow-up in the D2.*mdx* study vs. 6 months of follow-up in the present study).

Excessive transgene expression can have harmful effects, particularly when heightened protein synthesis overwhelms cellular degradative systems, potentially increasing metabolic demand on an already compromised heart. As also pointed out by Hart et al.,[60] the accumulation of MD may saturate the ubiquitin-proteasome system (UPS). This proteostatic imbalance is especially problematic in cardiomyocytes, which are vulnerable to stress from misfolded or excess proteins.[61] Disruption of protein homeostasis in these cells has been observed in hypertrophic and dilated cardiomyopathy.[62] In dystrophic muscle expressing an MD variant that retains only a subset of the native dystrophin's binding domains and interactors, such an imbalance in protein homeostasis may disrupt the fragile functional equilibrium of the cardiomyocytes. In any case, further investigations are needed to elucidate the mechanisms that could be involved in the cardiac abnormalities observed in the DMD context following MD overexpression.

Finally, as the current study does not exclude that exogenous MD-overexpression-induced adverse effects on cardiac electrophysiology at very high doses, even in the DMD context, cardiac rhythm should be carefully monitored in DMD clinical trials.

MATERIALS AND METHODS

Experimental design

The objective of this study was to investigate the safety of the systemic administration of high doses of the GNT0004 product in both WT and DMD^{mdx} rats and, more specifically, to determine the biological process that could explain the occurrence of sudden deaths observed in WT rats after the injection of a 4.2×10^{14} vg/kg dose. Sample sizes were determined using power analyses based on the effect size observed during preliminary experiments. All animals were randomly selected for GNT0004 or vehicle treatment. To avoid any bias, all data acquisition and analyses performed during this study were done by experimenters who were blind to the animal genotype (WT or DMD^{mdx}) and treatment conditions (vehicle or GNT0004). Because of the technical constraints inherent to the DMD^{mdx} rat model and the cardiac evaluations, the present study was performed in non-GLP conditions. The facilities or teams where the study was conducted are under quality management systems (GLP accreditation, ISO9001:2015, or internal quality management systems implemented to ensure the quality and integrity of the nonclinical study, including organization, personnel, training, facilities and equipment, written procedures, and global documentation).

Vector design and production

The GNT0004 product (rAAV2/8-Spc5.12-hMD1-spA) contained a species-specific codon and mRNA-sequence-optimized hMD cDNA (hMD1, ΔR4–23/ΔCT) under the control of a synthetic muscle and cardiac Spc5.12 promoter.[18,30] The GNT0004 product batch used in this study was produced in HEK293T cells, cultured in suspension, using a manufacturing process similar to the subsequent phase 1/2 clinical trial. Briefly, cells were tri-transfected with the AAV cassette plasmid, the AAV Rep and Cap gene plasmid, and a "helper" plasmid containing adenoviral genes that are essential for the AAV life cycle. Twenty-four hours after transfection, the cells were treated with benzonase (Merck Millipore, Molsheim, France). Three days after transfection, the cells were chemically lysed and filtered. Viral capsids were purified by affinity chromatography (Poros AAV8, Thermo Fisher Scientific, Illkirch, France) concentrated on hollow fibers (Repligen, Boston, Massachusetts), formulated in sterile Ringer's lactate solution (Lavoisier, Paris, France) containing 0.001% of pluronic-F68 (Merck Millipore), sterile filtered, aliquoted, and frozen at $\leq -65°C$ in a temperature-controlled freezer. Several quality control tests (physical titer, infectious titer, identity of viral proteins (VP), *in vitro* expression, pH, osmolality, level of endotoxins, aggregates, sterility, and stability) were performed before administration to animals.

Animals

A total of 31 DMD^{mdx} rats and 31 Sprague-Dawley WT rats (littermates) were used in this study. As DMD affects mainly male patients, only male rats were used in this study. Animals were randomly assigned to the different experimental groups. All DMD^{mdx} and WT rats were from the same genetic background (Sprague-Dawley) and derived from the same litters. All rats were bred (using carrier females and WT rats), housed, and handled at the Boisbonne Center for Gene Therapy (ONIRIS, Nantes, France). In accordance with French and European regulations for the protection of animals used for experimental and other scientific purposes, the Boisbonne Center's study protocol was approved by the French Ministry for Higher Education, Research and Innovation (MESRI) following an evaluation by the Comité d'Ethique en Expérimentation Animale, CEEA, Pays de la Loire (national committee for the protection of animals used for scientific purposes). Final approval of the MESRI was under #APAFIS#18179-2018122108594677 v.2, dated March 28th, 2019. Animals were housed (temperature, air changes, light cycles, and housing) according to Directive 2010/63/EU of the

European Parliament and the Council of September 22nd, 2010, on the protection of animals used for scientific purposes and national transposition, February 1st, 2013, and according to biosafety level 1 requirements.

Vector delivery, animal follow-up, and euthanasia

GNT0004 and the corresponding vehicle were administered on injection day (day 0) by a single i.v. injection into the tail vein of anesthetized animals. At day 0, all animals were ≈4 weeks old (28–34 days). The same vector batch was administered to all animals in the study. The vehicle contained Ringer's lactate solution (Lavoisier) containing 0.001% of pluronic-F68 (Merck Millipore). Prior to injection, the rAAV vector was diluted in vehicle solution to obtain the required amount of GNT0004 per injection (4.2×10^{14} or 2.1×10^{14} vg/kg) in a final volume of 16.8 mL/kg. The maximum flow rate for injection was ≈0.5 mL/min. The anesthesia protocol was adapted, given the potential for malignant hyperthermia-like syndrome resulting in death in DMD^{mdx} rats anesthetized with isoflurane.[36] The analgesic buprenorphine (0.04 mg/kg, subcutaneous) was administered between 30 min and 6 h before anesthesia with etomidate (8 mg/kg single intraperitoneal [i.p.] administration).

After injection, general condition, behavior, and activity were assessed daily, as well as morbidity/mortality. Full clinical observation was performed once per week. Functional and neurobehavioral tests were performed once per month on animals observed individually in a cage without sawdust in a quiet room. BW was recorded at day 0 and then weekly after injection until euthanasia.

At the end of the study (6 months p.i.), premedication analgesia of morphine (2.5 mg/kg, i.p.) was delivered before induction of deeper anesthesia with ketamine (20 mg/kg, i.p.). After creating an incision in the abdomen, 3–4 mL of whole blood was obtained at the level of the vena cava or the renal vein. After blood sample collection, animals were euthanized by i.v. injection of 30–35 mg/kg potassium chloride. Necropsy and pathological analyses were conducted blindly under the supervision of a Doctor of Veterinary Medicine (DVM), European Board-certified pathologist.

hMD1 and endogenous rDys protein expression analysis

For the animals that survived to 6 months p.i., qualitative hMD1 protein expression was analyzed by western blot using the MANEX 1011C antibody (which recognizes exons 10–11 of dystrophin, obtained from the MDA monoclonal antibody resource). Analyses were done on *biceps femoris*, heart, and diaphragm tissue samples obtained at euthanasia and directly snap frozen in liquid nitrogen before storage at $\leq -65^{\circ}$C. Total protein was extracted from tissue samples using a homemade radioimmunoprecipitation assay (RIPA) buffer containing protease inhibitor cocktail, and total protein extracts were loaded onto a NuPAGE Novex 3%–8% Tris acetate gel and analyzed using the NuPAGE large protein blotting kit (Thermo Fischer Scientific). Membranes were hybridized with the anti-dystrophin MANEX 1011C antibody and with a secondary anti-mouse IgG HRP-conjugated antibody (Dako, Les Ulis, France). As a protein loading control, the same membrane was also hybridized with an anti-rat α-tubulin antibody (Sigma-Aldrich, Saint-Quentin-Fallavier, France) and with a secondary anti-mouse IgG HRP-conjugated antibody (Dako). Immunoblots were visualized using the enhanced chemiluminescence (ECL) analysis system (Thermo Fisher Scientific).

On the same samples, quantitative hMD1 protein expression was analyzed by Simple Western (for hMD1) using the NCL-DYSB antibody (Leica Biosystems, Nanterre, France). Total proteins were extracted from 20×15 μm tissue sections using RIPA extraction buffer (Thermo Fischer Scientific) containing Halt 3× (Thermo Fischer Scientific) and 5 mM EDTA. Simple Western was performed using the capillary-based immunoassay system JESS (Protein Simple from Bio-Techne, Minneapolis, Minnesota). Total proteins were diluted to the appropriate concentration in sample buffer (Bio-Techne) and denatured at 95°C for 5 min. The NCL-DYSB antibody was diluted to 1/10 in antibody diluent, and an anti-mouse poly HRP (Bio-Techne) was used for detection. For total protein normalization, samples were run separately on capillaries containing the total protein assay (DM-TP01). The GA 721 calibrating sample was loaded on each Simple Western run to avoid experimental bias. Total proteins and hMD1 protein signals were analyzed using the Compass software from ProteinSimple (Bio-Techne), and data were transferred to Excel for final quantification. To avoid bias in the analysis, samples were distributed evenly between groups on each run.

As the antibody used in Simple Western does not recognize rat endogenous dystrophin, this protein was quantified using a traditional western blot using the MANEX 1011C antibody (DHSB, Iowa City, Iowa). The same protein extracts (15 μg) were diluted in lysis solution and denatured with a 4× Laemmli sample buffer (Bio-Rad, Marnes-la-coquette, France), containing 10% β-mercapthoethanol. Denaturation was carried out at 95°C for 5 min. Total proteins were then loaded onto a 4%–15% Criterion TGX Precast Midi Protein Gel (Bio-Rad). Proteins were then transferred onto a 0.45 μM nitrocellulose membrane. Membranes were stained with Revert total protein stain (Li-Cor, Bad Homburg, Germany). Total proteins were imaged at 700 nm using the laser scanning Odyssey Infrared Imaging System (Li-Cor) and quantified by the Empiria Studio Software (Li-Cor). After total protein imaging, membranes were hybridized with the anti-dystrophin MANEX 1011C (DHSB) antibody and then with a secondary antibody coupled to an IRDye 800 CW infrared dye (LiCor). Rat endogenous dystrophin signals were detected using the Odyssey system and quantified with Image Studio.

Immunohistochemistry

For all animals euthanized at 6 months p.i., tissue samples obtained during necropsy (*biceps femoris* muscle and heart) were snap frozen in liquid-nitrogen-cooled isopentane and then stored at $\leq -65^{\circ}$C. Sections were labeled for detection of endogenous rDys and hMD1 using the mouse monoclonal anti-dystrophin antibodies NCL-DYS2 (Leica Biosystems) and NCL-DYS3 (Leica Biosystems), respectively. NCL-DYS3, a mouse monoclonal IgG2a antibody,

specifically recognizing the N-terminal domain (amino acids 67–173) of the human dystrophin (and thus not the native rDys), was used for hMD1 staining. NCL-DYS2, a mouse monoclonal IgG1 antibody that recognizes the C-term domain of the native dystrophin from different species, was used for specific staining of native rDys. hMD1 does not contain the C-term domain of dystrophin; therefore, the NCL-DYS2 antibody does not stain hMD1-positive fibers. For tissue topography analysis, basal membranes were labeled using a polyclonal antibody against laminin (1:1,000; Sigma-Aldrich), and nuclei were counterstained with DAPI (BioStatus, Loughborough, United Kingdom). The percentages of DYS2- and DYS3-positive fibers were calculated in 3 randomly selected microscopic fields per muscle. Labeling of two-thirds of the fiber was required to be considered positive for the purposes of quantification. All measurements were automatically performed using Nikon Imaging software (Nikon, Cergy-Pontoise, France).

For DAPC and utrophin analyses, section samples were co-immunostained for α-sarcoglycan (DHSB), γ-sarcoglycan (Leica Biosystems), β-dystroglycan (Leica Biosystems), or utrophin (Santa Cruz Biotechnology, Heidelberg, France) antibodies.

Histopathological analysis

For all animals, tissue samples obtained during necropsy (*biceps femoris* muscle, diaphragm, and heart) were fixed in 10% neutral buffered formalin and embedded in paraffin wax. Sections were cut and stained with hematoxylin, eosin, and saffron (HES) for histopathological evaluation. For the samples obtained 6 months p.i., muscle lesions were then semi-quantitatively scored (in *biceps femoris*, diaphragm, and cardiac muscles) using the following scoring system: 0, absence of lesions; 1, presence of some regenerative activity as evidenced by centronucleated fibers and small foci of regeneration; 2, presence of degenerated fibers, isolated or in small clusters; and 3, tissue remodeling and fiber replacement with fibrotic or adipose tissue. In hearts, scoring was based on the intensity of fibrosis (1, focal; 2, focal extensive) and the presence of degenerative fibers (score of 3). A mean muscle score was then calculated to identify any improvement in the histopathological pattern between the different experimental groups. Additional sections from paraffin-embedded samples of the heart were stained with picrosirius red F3B (Sigma-Aldrich) for collagen visualization in the connective tissue. Picrosirius-positive areas in heart sections were quantified using Nikon Imaging software (Nikon).

Skeletal muscle function analysis

Muscle function was assessed in all animals using the grip test at ≈3 and ≈6 months p.i., for the animals that survived to these time points. Rats were placed with their forepaws on a T-bar and gently pulled backward until they released their grip.[33] A grip meter (Bio-GT3, BIOSEB, Vitrolles, France) attached to a force transducer measured the peak force generated. Five tests (trials) were performed in sequence with an interval of 20–30 s between tests. The highest grip value obtained during these five trials was considered the maximum forelimb grip force. The five values obtained at each time point were also compared (cross-trial evolution), and the reduction in strength between the first and the last test was taken as an index of fatigue. Results were normalized to BW (g/g BW).

Cardiac function analysis: 2D echocardiography and pulsed Doppler

At the end of the experimental study period (i.e., 6 months p.i., just before euthanasia), ECG parameters were measured conventionally under anesthesia (16 mg/kg etomidate i.p., administered in 2 injections separated by 3–5 min) in all animals that were still alive at this time point. Six-lead ECG recordings were taken using subcutaneous 25G electrodes and an analog-digital converter (IOX 1.585, EMKA Technologies, France) for monitoring and offline analysis (ECG Auto v.3.2.0.2, EMKA Technologies). Classical intervals and segments were measured. The QT interval was normalized to the heart rate to calculate the QTc, enabling comparison of the duration of depolarization-repolarization between individual rats and experimental groups. After recording the aforementioned parameters (still at 6 months p.i., just before euthanasia), 2D echocardiography was performed using a Vivid 7 Dimension ultrasound system (GE Healthcare) with a 14-MHz transducer. To detect possible structural remodeling, the left ventricular end-diastolic diameter and the free wall end-diastolic thickness were measured during diastole from long- and short-axis images obtained by M-mode echocardiography. In addition, systolic function was assessed by measuring the ejection fraction, and ventricular filling velocity was evaluated by measuring transmitral flow using pulsed Doppler with an apical 4-chamber orientation. Diastolic dysfunction was evaluated based on Doppler-derived early (E) and late (A) diastolic velocities, the E/A ratio, and determination of isovolumetric relaxation and DTs.

Cardiac function analysis: Non-invasive ECG telemetry

Cardiac function was assessed on all animals using non-invasive conscious ECG telemetry (rate and rhythm of heartbeats). The electrocardiographic leads were attached to the skin using sticky patches in a Lead II configuration and specific jackets developed by Data Science International (DSI, Saint Paul, Minnesota). Cardiac rhythm was monitored using the radiotelemetry data acquisition program Dataquest ART (v.4.39, DSI). To minimize variability, ECG analyses were conducted by a single reader using semi-automated methods. ECG monitoring by telemetry in conscious animals was performed continuously every 8 days (from 1 month p.i. to the week before euthanasia) for 22-h cycles. For each day of ECG monitoring, 2 h were required to equip and remove the equipment from the rats. Rats were monitored from approximately 11:00 a.m. to 9:00 a.m. the following day. Data were analyzed and presented using Microsoft Excel (Microsoft, Redmond, Washington). To evaluate the presence of rhythm disorders, sampling was performed at the first 3 min of each hour to measure the number of events, qualify the nature of the observed events, and determine their potential severity. A decision tree was generated to empirically classify these events into three categories: events with a low potential impact on cardiac electrical function (ripple-type events and burst-type events); events with a

middle (medium) impact on cardiac electrical function (more conventional arrhythmias [e.g., ventricular extrasystoles] that did not alter the sinus rhythm); and events with a high impact on cardiac electrical function (very frequent EVBs, polymorphic extrasystole, tachycardia, pauses, and a pause corresponding to a temporary stop of sinus activity). For each rhythm disorder subtype, the number of events during each analyzed period was determined. These results were qualitatively analyzed using hierarchical clustering but were also quantitatively analyzed by comparing the mean number of events measured in each animal in each recording session.

Statistics

All statistical analyses were performed using GraphPad Prism 8 (Prism, Boston, Massachusetts). Data are presented as mean ± SEM. For all parameters, the potential differences between groups were evaluated using the nonparametric Kruskal-Wallis test. When significant overall effects were detected, differences between experimental groups were assessed by Dunn's post hoc test. Repeated grip force values were analyzed using the Friedman test, followed by Dunn's post hoc test. $p \leq 0.05$ were considered statistically significant.

DATA AND CODE AVAILABILITY

All data are available in the main text or the supplemental information. Upon request and subject to review, the corresponding authors will provide the data that support the findings of this study.

ACKNOWLEDGMENTS

This work has been achieved in the frame of a collaboration license agreement with Sarepta Therapeutics. We thank Sarepta Therapeutics for supporting scientific discussions. We also thank all personnel of the Boisbonne Center for Gene Therapy (ONIRIS, INSERM, Nantes, France) for handling and care of the animals included in this study. We also thank the staff of the Preclinical Analytics Core (TaRGeT lab – UMR1089, Nantes Université, CHU Nantes, INSERM, Nantes, France), the Gene Therapy Immunology Core (TaRGeT lab – UMR1089, Nantes Université, CHU Nantes, INSERM, Nantes, France), the Therassay Core (Capacités, Nantes Université, France), the APEX Core (INRAE, ONIRIS, Nantes, France), GenoSafe (Evry, France), and the Genethon preclinical evaluation platform (Evry, France) for technical assistance. We thank the MDA Monoclonal Antibody Resource for providing the MANEX 1011C antibody. We thank Coralie Maizeray (Genethon, Evry, France) for her assistance in generating the graphical abstract and Amanda Finan-Marchi (Genethon, Evry, France) for reviewing the language of the manuscript. The graphical abstract was created using BioRender.com. The published version of the figure is available at https://BioRender.com/9uzynww.

AUTHOR CONTRIBUTIONS

Conceptualization, C.L.G., S.M., S. Blaie, and S. Braun; investigation, C.L.G., G.T., T.L., L.B., G.C., C.V.M., A. Lancelot, C.G., A. Lafoux, D.A., C.J., and A.H.; methodology, G.T., T.L., L.B., A. Lafoux, C.C., O.A., C.H., and N.D.; project administration, C.L.G., S.M., E. C., S. Blaie, and G.P.; supervision, C.L.G. and S. Braun; visualization, C.L.G., G.T., T.L., L.B., A. Lafoux, C.C., and E.C.; writing – original draft, C.L.G. and S. Braun; writing – review & editing, all co-authors.

DECLARATION OF INTERESTS

C.L.G. is a co-author of a patent for systemic treatment of dystrophic pathologies (EP3044319A1, dated June 27, 2014).

SUPPLEMENTAL INFORMATION

Supplemental information can be found online at https://doi.org/10.1016/j.ymthe.2025.10.041.

REFERENCES

1. Hoffman, E.P., Brown, R.H., and Kunkel, L.M. (1987). Dystrophin: The Protein Product of the Duchenne Muscular Dystrophy Locus. Cell *51*, 919–928.
2. Judge, L.M., Haraguchiln, M., and Chamberlain, J.S. (2006). Dissecting the signaling and mechanical functions of the dystrophin-glycoprotein complex. J. Cell Sci. *119*, 1537–1546. https://doi.org/10.1242/jcs.02857.
3. Duan, D., Goemans, N., Takeda, S., Mercuri, E., and Aartsma-Rus, A. (2021). Duchenne muscular dystrophy. Nat. Rev. *7*, 13.
4. Peng, Y.-M., Bai, Y.-R., Li, R.-F., Xu, Y.-X., Liu, H.-M., Yuan, S., and Jin, Z.-P. (2025). The prospects and challenges of small molecule drugs in the treatment of Duchenne muscular dystrophy. Eur. J. Med. Chem. *297*, 117980. https://doi.org/10.1016/j.ejmech.2025.117980.
5. Bushby, K., Finkel, R., Birnkrant, D.J., Case, L.E., Clemens, P.R., Cripe, L., Kaul, A., Kinnett, K., McDonald, C., Pandya, S., et al. (2010). Diagnosis and management of Duchenne muscular dystrophy, part 2: implementation of multidisciplinary care. Lancet Neurol. *9*, 177–189. https://doi.org/10.1016/S1474-4422(09)70272-8.
6. Mendell, J.R., and Lloyd-Puryear, M. (2013). Report of MDA muscle disease symposium on newborn screening for Duchenne muscular dystrophy. Muscle Nerve *48*, 21–26. https://doi.org/10.1002/mus.23810.
7. Rall, S., and Grimm, T. (2012). Survival in Duchenne muscular dystrophy. Acta Myol. *31*, 117–120.
8. Birnkrant, D.J., Bushby, K., Bann, C.M., Apkon, S.D., Blackwell, A., Brumbaugh, D., Case, L.E., Clemens, P.R., Hadjiyannakis, S., Pandya, S., et al. (2018). Diagnosis and management of Duchenne muscular dystrophy, part 1: diagnosis, and neuromuscular, rehabilitation, endocrine, and gastrointestinal and nutritional management. Lancet Neurol. *17*, 251–267. https://doi.org/10.1016/S1474-4422(18)30024-3.
9. Szabo, S.M., Salhany, R.M., Deighton, A., Harwood, M., Mah, J., and Gooch, K.L. (2021). The clinical course of Duchenne muscular dystrophy in the corticosteroid treatment era: a systematic literature review. Orphanet J. Rare Dis. *16*, 237. https://doi.org/10.1186/s13023-021-01862-w.
10. Fortunato, F., Rossi, R., Falzarano, M.S., and Ferlini, A. (2021). Innovative Therapeutic Approaches for Duchenne Muscular Dystrophy. J. Clin. Med. *10*, 820. https://doi.org/10.3390/jcm10040820.
11. Mackenzie, S.J., Nicolau, S., Connolly, A.M., and Mendell, J.R. (2021). Therapeutic Approaches for Duchenne Muscular Dystrophy: Old and New. Semin. Pediatr. Neurol. *37*, 100877. https://doi.org/10.1016/j.spen.2021.100877.
12. Takeda, S., Clemens, P.R., and Hoffman, E.P. (2021). Exon-Skipping in Duchenne Muscular Dystrophy. J. Neuromuscul. Dis. *8*, S343–S358. https://doi.org/10.3233/JND-210682.
13. Politano, L. (2021). Read-through approach for stop mutations in Duchenne muscular dystrophy. An update. Acta Myol. *40*, 43–50.
14. Pupo, A., Fernández, A., Low, S.H., François, A., Suárez-Amarán, L., and Samulski, R.J. (2022). AAV vectors: The Rubik's cube of human gene therapy. Mol. Ther. *30*, 3515–3541. https://doi.org/10.1016/j.ymthe.2022.09.015.
15. Issa, S.S., Shaimardanova, A.A., Solovyeva, V.V., and Rizvanov, A.A. (2023). Various AAV Serotypes and Their Applications in Gene Therapy: An Overview. Cells *12*, 785. https://doi.org/10.3390/cells12050785.
16. Harper, S.Q., Hauser, M.A., DelloRusso, C., Duan, D., Crawford, R.W., Phelps, S.F., Harper, H.A., Robinson, A.S., Engelhardt, J.F., Brooks, S.V., and Chamberlain, J.S. (2002). Modular flexibility of dystrophin: implications for gene therapy of Duchenne muscular dystrophy. Nat. Med. *8*, 253–261. https://doi.org/10.1038/nm0302-253.
17. Duan, D. (2018). Systemic AAV Micro-dystrophin Gene Therapy for Duchenne Muscular Dystrophy. Mol. Ther. *26*, 2337–2356. https://doi.org/10.1016/j.ymthe.2018.07.011.
18. Foster, H., Sharp, P.S., Athanasopoulos, T., Trollet, C., Graham, I.R., Foster, K., Wells, D.J., and Dickson, G. (2008). Codon and mRNA Sequence Optimization of Microdystrophin Transgenes Improves Expression and Physiological Outcome in Dystrophic mdx Mice Following AAV2/8 Gene Transfer. Mol. Ther. *16*, 1825–1832. https://doi.org/10.1038/mt.2008.186.
19. Le Guiner, C., Servais, L., Montus, M., Larcher, T., Fraysse, B., Moullec, S., Allais, M., François, V., Dutilleul, M., Malerba, A., et al. (2017). Long-term microdystrophin

gene therapy is effective in a canine model of Duchenne muscular dystrophy. Nat. Commun. *8*, 16105. https://doi.org/10.1038/ncomms16105.

20. Ramos, J.N., Hollinger, K., Bengtsson, N.E., Allen, J.M., Hauschka, S.D., and Chamberlain, J.S. (2019). Development of Novel Micro-dystrophins with Enhanced Functionality. Mol. Ther. *27*, 623–635. https://doi.org/10.1016/j.ymthe.2019.01.002.
21. Potter, R.A., Griffin, D.A., Heller, K.N., Peterson, E.L., Clark, E.K., Mendell, J.R., and Rodino-Klapac, L.R. (2021). Dose-Escalation Study of Systemically Delivered rAAVrh74.MHCK7.micro-dystrophin in the *mdx* Mouse Model of Duchenne Muscular Dystrophy. Hum. Gene Ther. *32*, 375–389. https://doi.org/10.1089/hum.2019.255.
22. Le Guiner, C., Xiao, X., Larcher, T., Lafoux, A., Huchet, C., Toumaniantz, G., Adjali, O., Anegon, I., Remy, S., Grieger, J., et al. (2023). Evaluation of an AAV9-mini-dystrophin gene therapy candidate in a rat model of Duchenne muscular dystrophy. Mol. Ther. Methods Clin. Dev. *30*, 30–47. https://doi.org/10.1016/j.omtm.2023.05.017.
23. Le Guiner, C., Fraysse, B., Testault, I., Wahbi, K., Couzinie, C., Adjali, O., Dickson, G., Creoff, E., Veron, P., and Braun, S. (2023). More than Nine Year Survival of a GRMD Dog after Injection of AAV-Microdystrophin Gene Therapy. In 26th Annual Meeting of the American Society of Gene & Cell Therapy.
24. Birch, S.M., Lawlor, M.W., Conlon, T.J., Guo, L.-J., Crudele, J.M., Hawkins, E.C., Nghiem, P.P., Ahn, M., Meng, H., Beatka, M.J., et al. (2023). Assessment of systemic AAV-microdystrophin gene therapy in the GRMD model of Duchenne muscular dystrophy. Sci. Transl. Med. *15*, eabo1815. https://doi.org/10.1126/scitranslmed.abo1815.
25. Foltz, S.J., Qian, R., Yang, J., Elliott, K., Heithoff, B.P., Jasick, H., Smith, J.B., Karumuthil-Melethil, S., Qiao, C., Liu, Y., and Danos, O. (2025). Enhanced therapeutic potential of a microdystrophin with an extended C-terminal domain. Mol. Ther. Methods Clin. Dev. *33*, 101519. https://doi.org/10.1016/j.omtm.2025.101519.
26. Deng, J., Zhang, J., Shi, K., and Liu, Z. (2022). Drug development progress in duchenne muscular dystrophy. Front. Pharmacol. *13*, 950651. https://doi.org/10.3389/fphar.2022.950651.
27. Braun, S. (2025). Duchenne muscular dystrophy, one of the most complicated diseases for gene therapy. J. Transl. Genet. Genom. *9*, 35–47. https://doi.org/10.20517/jtgg.2024.79.
28. Byrne, B.J., Flanigan, K.M., Matesanz, S.E., Finkel, R.S., Waldrop, M.A., D'Ambrosio, E.S., Johnson, N.E., Smith, B.K., Bönnemann, C., Carrig, S., et al. (2025). Current clinical applications of AAV-mediated gene therapy. Mol. Ther. *33*, 2479–2516. https://doi.org/10.1016/j.ymthe.2025.04.045.
29. Mendell, J.R., Sahenk, Z., Lehman, K.J., Lowes, L.P., Reash, N.F., Iammarino, M.A., Alfano, L.N., Lewis, S., Church, K., Shell, R., et al. (2024). Long-term safety and functional outcomes of delandistrogene moxeparvovec gene therapy in patients with Duchenne muscular dystrophy: A phase 1/2a nonrandomized trial. Muscle Nerve *69*, 93–98. https://doi.org/10.1002/mus.27955.
30. Li, X., Eastman, E.M., Schwartz, R.J., and Draghia-Akli, R. (1999). Synthetic muscle promoters: activities exceeding naturally occurring regulatory sequences. Nat. Biotechnol. *17*, 241–245. https://doi.org/10.1038/6981.
31. Escobar, H., Schöwel, V., Spuler, S., Marg, A., and Izsvák, Z. (2016). Full-length Dysferlin Transfer by the Hyperactive Sleeping Beauty Transposase Restores Dysferlin-deficient Muscle. Mol. Ther. Nucleic Acids *5*, e277. https://doi.org/10.1038/mtna.2015.52.
32. Liu, M., Cook, E., Dai, Y., Ehlert, E., Du Plessis, F., Lubelski, J., Sleczka, B.G., Shipkova, P., Li, Z., Gamse, J., et al. (2025). Systemic delivery of AAV5, AAV8, and AAV9 packaging a C5-12-microdystrophin-FLAG expression cassette in non-human primates. Mol. Ther. Methods Clin. Dev. *33*, 101411. https://doi.org/10.1016/j.omtm.2025.101411.
33. Larcher, T., Lafoux, A., Tesson, L., Remy, S., Thepenier, V., François, V., Le Guiner, C., Goubin, H., Dutilleul, M., Guigand, L., et al. (2014). Characterization of Dystrophin Deficient Rats: A New Model for Duchenne Muscular Dystrophy. PLoS One *9*, e110371. https://doi.org/10.1371/journal.pone.0110371.
34. Owen, R.A., and Heywood, R. (1986). Age-Related Variations in Renal Structure and Function in Sprague-Dawley Rats. Toxicol. Pathol. *14*, 158–167. https://doi.org/10.1177/019262338601400203.
35. Gurnaney, H., Brown, A., and Litman, R.S. (2009). Malignant Hyperthermia and Muscular Dystrophies. Anesth. Analg. *109*, 1043–1048. https://doi.org/10.1213/ane.0b013e3181aa5cf6.
36. Creisméas, A., Gazaille, C., Bourdon, A., Lallemand, M.-A., François, V., Allais, M., Ledevin, M., Larcher, T., Toumaniantz, G., Lafoux, A., et al. (2021). TRPC3, but not TRPC1, as a good therapeutic target for standalone or complementary treatment of DMD. J. Transl. Med. *19*, 519. https://doi.org/10.1186/s12967-021-03191-9.
37. Vaillend, C., and Chaussenot, R. (2017). Relationships linking emotional, motor, cognitive and GABAergic dysfunctions in dystrophin-deficient *mdx* mice. Hum. Mol. Genet. *26*, 1041–1055. https://doi.org/10.1093/hmg/ddx013.
38. Caudal, D., François, V., Lafoux, A., Ledevin, M., Anegon, I., Le Guiner, C., Larcher, T., and Huchet, C. (2020). Characterization of brain dystrophins absence and impact in dystrophin-deficient Dmdmdx rat model. PLoS One *15*, e0230083. https://doi.org/10.1371/journal.pone.0230083.
39. Szabó, P.L., Ebner, J., Koenig, X., Hamza, O., Watzinger, S., Trojanek, S., Abraham, D., Todt, H., Kubista, H., Schicker, K., et al. (2021). Cardiovascular phenotype of the *Dmdmdx* rat – a suitable animal model for Duchenne muscular dystrophy. Dis. Model. Mech. *14*, dmm047704. https://doi.org/10.1242/dmm.047704.
40. Festing, M.F.W. (2014). Evidence Should Trump Intuition by Preferring Inbred Strains to Outbred Stocks in Preclinical Research. ILAR J. *55*, 399–404. https://doi.org/10.1093/ilar/ilu036.
41. Martin, M.D., Danahy, D.B., Hartwig, S.M., Harty, J.T., and Badovinac, V.P. (2017). Revealing the Complexity in CD8 T Cell Responses to Infection in Inbred C57B/6 versus Outbred Swiss Mice. Front. Immunol. *8*, 1527. https://doi.org/10.3389/fimmu.2017.01527.
42. Rodriguez-Cruz, M., Almeida-Becerril, T., Atilano-Miguel, S., Cárdenas-Conejo, A., and Bernabe-García, M. (2020). Natural History of Serum Enzyme Levels in Duchenne Muscular Dystrophy and Implications for Clinical Practice. Am. J. Phys. Med. Rehabil. *99*, 1121–1128.
43. Bourdon, A., François, V., Zhang, L., Lafoux, A., Fraysse, B., Toumaniantz, G., Larcher, T., Girard, T., Ledevin, M., Lebreton, C., et al. (2022). Evaluation of the dystrophin carboxy-terminal domain for micro-dystrophin gene therapy in cardiac and skeletal muscles in the DMDmdx rat model. Gene Ther. *29*, 520–535. https://doi.org/10.1038/s41434-022-00317-6.
44. Arechavala-Gomeza, V., Kinali, M., Feng, L., Guglieri, M., Edge, G., Main, M., Hunt, D., Lehovsky, J., Straub, V., Bushby, K., et al. (2010). Revertant fibres and dystrophin traces in Duchenne muscular dystrophy: implication for clinical trials. Neuromuscul. Disord. *20*, 295–301. https://doi.org/10.1016/j.nmd.2010.03.007.
45. Pigozzo, S.R., Da Re, L., Romualdi, C., Mazzara, P.G., Galletta, E., Fletcher, S., Wilton, S.D., and Vitiello, L. (2013). Revertant Fibers in the mdx Murine Model of Duchenne Muscular Dystrophy: An Age- and Muscle-Related Reappraisal. PLoS One *8*, e72147. https://doi.org/10.1371/journal.pone.0072147.
46. Kleopa, K.A., Drousiotou, A., Mavrikiou, E., Ormiston, A., and Kyriakides, T. (2006). Naturally occurring utrophin correlates with disease severity in Duchenne muscular dystrophy. Hum. Mol. Genet. *15*, 1623–1628. https://doi.org/10.1093/hmg/ddl083.
47. Janghra, N., Morgan, J.E., Sewry, C.A., Wilson, F.X., Davies, K.E., Muntoni, F., and Tinsley, J. (2016). Correlation of Utrophin Levels with the Dystrophin Protein Complex and Muscle Fibre Regeneration in Duchenne and Becker Muscular Dystrophy Muscle Biopsies. PLoS One *11*, e0150818. https://doi.org/10.1371/journal.pone.0150818.
48. de Jong, S., van Veen, T.A.B., van Rijen, H.V.M., and de Bakker, J.M.T. (2011). Fibrosis and cardiac arrhythmias. J. Cardiovasc. Pharmacol. *57*, 630–638. https://doi.org/10.1097/FJC.0b013e318207a35f.
49. Morita, N., Mandel, W.J., Kobayashi, Y., and Karagueuzian, H.S. (2014). Cardiac fibrosis as a determinant of ventricular tachyarrhythmias. J. Arrhythm. *30*, 389–394. https://doi.org/10.1016/j.joa.2013.12.008.
50. Nguyen, M.-N., Kiriazis, H., Gao, X.-M., and Du, X.-J. (2017). Cardiac Fibrosis and Arrhythmogenesis. Compr. Physiol. *7*, 1009–1049. https://doi.org/10.1002/cphy.c160046.
51. Nakai, H., Thomas, C.E., Storm, T.A., Fuess, S., Powell, S., Wright, J.F., and Kay, M.A. (2002). A Limited Number of Transducible Hepatocytes Restricts a Wide-Range Linear Vector Dose Response in Recombinant Adeno-Associated

Virus-Mediated Liver Transduction. J. Virol. *76*, 11343–11349. https://doi.org/10.1128/jvi.76.22.11343-11349.2002.

52. Lek, A., Atas, E., Hesterlee, S.E., Byrne, B.J., and Bönnemann, C.G. (2023). Meeting Report: 2022 Muscular Dystrophy Association Summit on 'Safety and Challenges in Gene Transfer Therapy'. J. Neuromuscul. Dis. *10*, 327–336. https://doi.org/10.3233/JND-221639.

53. Ervasti, J.M., and Campbell, K.P. (1993). Dystrophin and the membrane skeleton. Curr. Opin. Cell Biol. *5*, 82–87. https://doi.org/10.1016/S0955-0674(05)80012-2.

54. Mias-Lucquin, D., Dos Santos Morais, R., Chéron, A., Lagarrigue, M., Winder, S.J., Chenuel, T., Pérez, J., Appavou, M.-S., Martel, A., Alviset, G., et al. (2020). How the central domain of dystrophin acts to bridge F-actin to sarcolemmal lipids. J. Struct. Biol. *209*, 107411. https://doi.org/10.1016/j.jsb.2019.107411.

55. Sveen, M.-L., Thune, J.J., Køber, L., and Vissing, J. (2008). Cardiac involvement in patients with limb-girdle muscular dystrophy type 2 and Becker muscular dystrophy. Arch. Neurol. *65*, 1196–1201. https://doi.org/10.1001/archneur.65.9.1196.

56. Lancioni, A., Rotundo, I.L., Kobayashi, Y.M., D'Orsi, L., Aurino, S., Nigro, G., Piluso, G., Acampora, D., Cacciottolo, M., Campbell, K.P., and Nigro, V. (2011). Combined deficiency of alpha and epsilon sarcoglycan disrupts the cardiac dystrophin complex. Hum. Mol. Genet. *20*, 4644–4654. https://doi.org/10.1093/hmg/ddr398.

57. Abriel, H., Rougier, J.-S., and Jalife, J. (2015). Ion Channel Macromolecular Complexes in Cardiomyocytes: Roles in Sudden Cardiac Death. Circ. Res. *116*, 1971–1988. https://doi.org/10.1161/CIRCRESAHA.116.305017.

58. Fanin, M., Danieli, G.A., Cadaldini, M., Miorin, M., Vitiello, L., and Angelini, C. (1995). Dystrophin-positive fibers in duchenne dystrophy: Origin and correlation to clinical course. Muscle Nerve *18*, 1115–1120. https://doi.org/10.1002/mus.880181007.

59. Yue, Y., Wasala, N.B., Bostick, B., and Duan, D. (2016). 100-fold but not 50-fold dystrophin overexpression aggravates electrocardiographic defects in the mdx model of Duchenne muscular dystrophy. Mol. Ther. Methods Clin. Dev. *3*, 16045. https://doi.org/10.1038/mtm.2016.45.

60. Hart, C.C., Lee, Y.I., Xie, J., Gao, G., Lin, B.L., Hammers, D.W., and Sweeney, H.L. (2024). Potential limitations of microdystrophin gene therapy for Duchenne muscular dystrophy. JCI Insight 9, e165869.

61. McLendon, P.M., and Robbins, J. (2015). Proteotoxicity and Cardiac Dysfunction. Circ. Res. *116*, 1863–1882. https://doi.org/10.1161/CIRCRESAHA.116.305372.

62. Predmore, J.M., Wang, P., Davis, F., Bartolone, S., Westfall, M.V., Dyke, D.B., Pagani, F., Powell, S.R., and Day, S.M. (2010). Ubiquitin Proteasome Dysfunction in Human Hypertrophic and Dilated Cardiomyopathies. Circulation *121*, 997–1004. https://doi.org/10.1161/CIRCULATIONAHA.109.904557.

YMTHE, Volume *34*

Supplemental Information

Dose- and genotype-dependent cardiac arrhythmia and sudden death in rats following microdystrophin gene therapy

Caroline Le Guiner, Gilles Toumaniantz, Thibaut Larcher, Sylvie Marchand, Laurine Buscara, Grégory Cedrone, Cladice Varela Moreira, Amandine Lancelot, Christophe Georger, Aude Lafoux, Célia Couzinié, David Augereau, Corinne Jounier, Agnès Hivonnait, Estelle Creoff, Stéphanie Blaie, Corinne Huchet, Oumeya Adjali, Nathalie Daniele, Gérald Perret, and Serge Braun

SUPPLEMENTAL MATERIALS AND METHODS

Vector genome quantification

For the animals which survived to 6 months p.i., vector quantification was based on transgene (hMD1)-specific qPCR analysis of genomic DNA(gDNA) from tissue samples (biceps femoris muscle, heart [left ventricle, right ventricle, left atrium, right atrium, interventricular septum], diaphragm, liver [central lobe], lung, kidney, adrenal glands) obtained just after sacrifice under conditions that minimized cross-contamination and avoided qPCR inhibition. In particular, the samples were directly snap-frozen in liquid nitrogen and then stored at ≤-65°C. gDNA was extracted from snap frozen tissues using TissueLyserII (Qiagen) and NucleoSpin® Tissue kits (Macherey Nagel) according to the manufacturer's instructions. Simplex qPCR analyses were conducted on a QuantStudio7 light cycler (Applied Biosystems) using 600 ng of gDNA. Reactions were performed in triplicate in a final volume of 30 µL containing template DNA, qPCR Rox mix (Abgene), 200 nmol/L of each primer, and 100 nmol/L of Taqman probe. Vector copy numbers were determined using the following primer/probe combination, designed to amplify a region of the hMD1 transgene: Forward primer : 5'- CCAACAAAGTGCCCTACTACATC-3', Reverse primer : 5'- GGTTGTGCTGGTCCAGGGCGT- 3', Probe : 5'- CCGAGCTGTACCAGAGCCTGGCC- 3'. For each sample, cycle threshold (Ct) values were compared with those obtained from a linearized standard curve derived from various dilutions of hMD1 expression plasmid. The Lower Limit of Quantification (LLOQ) of the assay was determined as 50 vector genomes per µg of gDNA.

Immunological analyses

For the animals which survived to 6 months p.i. and injected with Vehicle or with $4.2x10^{14}$ vg/kg of GNT0004, anti-hMD1 IgG was analyzed in sera obtained before injection and at 6 months p.i. by Western-blot. Briefly, hMD1-positive protein extracts acquired from HEK293 cells transfected with a pCMV-hMD1 plasmid were subjected to precast polyacrylamide gel electrophoresis using the NuPAGE Large Protein Blotting Kit (3%–8% Tris acetate precast polyacrylamide gel; Invitrogen) and subsequently blotted. After overnight saturation, the membranes were incubated with rat sera collected before and after treatment at a dilution of 1/500. After hybridization with a peroxidase-conjugated anti-rat IgG antibody, proteins were visualized by enhanced chemiluminescence. For each Western-blot membrane, an anti-Dystrophin antibody (MANEX1011C) was used as a positive control. To validate the specificity of the assay (anti-hMD1 IgG detection in rat sera), the results from sera tested on non-transfected (hMD1-negative) cell extracts were compared to those obtained for transfected (hMD1-positive) cell extracts.

On the same animals, anti-hMD1 and anti-AAV8 cellular responses were analyzed using IFNγ ELISpot assays (Mabtech) on splenocytes isolated from fresh spleen samples obtained at sacrifice. T-cell responses were evaluated with an IFN-γ ELISpot assay using the following antigens: overlapping peptide libraries covering the entire sequences of the hMDl protein (length, 15 mers; overlap, 10 amino acids; total peptides, 238) or of the AAV8 capsid protein (length, 15 mers; overlap, 10 amino acids; total peptides, 146). The hMDl peptide library was divided into five peptide pools, and the AAV8 peptide library into three peptide pools. For each specimen, the negative control consisted of unstimulated cells (Medium) and the positive control was cells stimulated with mitogenic Concanavalin A (ConA). Each stimulation antigen and control condition were tested in triplicate. IFN-γ secretion was measured as the number of spot-forming cells (SFC)

per 10^6 cells. The threshold of positivity for IFN- γ secretion was defined for each sample as three-fold the spot-forming cell (SFC)/10^6 value obtained for the negative control (Medium) or at least 50 SFC/10^6 cells.

<u>Table S1</u>: Summary of premature deaths observed during the course of the previous GLP toxicology study, per experimental group.

Experimental group	Duration of follow-up	Premature deaths / total animals	Days (D) of occurrence	Comments on previous condition	Cause of death determined by histopathology
**WT + Vehicle**	8 days p.i.	1/11	D4	Low body weight. Pallor, dyspnoea, aggressivity.	Cecal ulcer, considered as the cause of the death.
	93 days p.i.	0/10	N/A	N/A	N/A
	183 days p.i.	1/10	D182	Death just after water gavage for urine collection.<br>No clinical signs prior the death.	No lesion to report.<br>Misgavage was considered as the cause of the death.
**WT + 6x10^{13} vg/kg**	8 days p.i.	0/10	N/A	N/A	N/A
	93 days p.i.	0/10	N/A	N/A	N/A
	183 days p.i.	0/10	N/A	N/A	N/A
**WT + 4.2x10^{14} vg/kg**	8 days p.i.	0/10	N/A	N/A	N/A
	93 days p.i.	1/10	D76	No clinical signs before death	<u>Heart</u>: Minimal to slight focal or multifocal inflammatory cell infiltration and multifocal to diffuse vacuolation of myofibers, occasionally accompanied by congestion<br><u>Liver</u>: Minimal to moderate centrilobular hepatocytic vacuolation and congestion<br>**➔ Lesions suggest the heart being the primary organ for toxicity and death, with liver changes being anoxic in nature and consecutive to heart failure**
	183 days p.i.	7/10	D61 - D85 - D87<br>D93 - D121<br>D140 - D149	No clinical signs before death	
**TOTAL**		**10/91**			

p.i. = post-injection – N/A = not applicable

Table S2: Summary of premature deaths during the course of the present study, per experimental group.

Experimental group	Premature deaths / total animals	Death occurring during telemetry*	Animal found dead in housing cage	Day (D) of occurrence	Histopathological observations**	Cause of death determined by histopathology
**WT + Vehicle**	0 / 10	0	0	N/A	N/A	N/A
**WT + 2.1x10^{14} vg/kg**	4 / 11	0	1	D3	Thoracic and jugular hemorrhages<br>Lu: mediastinal hemorrhage<br>Li: Extra-medullary hematopoiesis<br>S: Extra-medullary hematopoiesis	Coagulation defect
		0	1	D78	Lu: Acute passive congestion	Inconclusive (autolysis of most of the tissues)
		1	0	D113	Lu: Acute vascular congestion and alveolar edema<br>K: Nephropathy (hyalin casts, glomerulopathy)	Chronic progressive nephropathy
		1	0	D137	Lu: Alveolar emphysema (terminal dyspnea)	No identified cause of death
**WT + 4.2x10^{14} vg/kg**	3 / 10	1	0	D96	Lu: Foci of spumous macrophage<br>Li: acute passive congestion	No identified cause of death
		0	1	D105	Lu: Acute passive congestion	Inconclusive (autolysis of most of the tissues)
		1	0	D180	Lu: Alveolar emphysema<br>K: Bowman capsule thickening	No identified cause of death
**TOTAL**	**7 / 31**	**4**	**3**			

Experimental group	Premature deaths / total animals	Death occurring during telemetry*	Animal found dead in housing cage	Day (D) of occurrence	Histopathological observations**	Cause of death determined by histopathology
**DMD^{mdx} + Vehicle**	3 / 11	1	0	D29	M: Dystrophic lesions + Malignant-hyperthermia lesions***<br>H: Ventricular dilatation + inflammatory foci<br>Lu: Acute passive congestion	Malignant hyperthermia-like syndrome / Cardiac failure (linked to DMD pathology)
		1	0	D54	M: Dystrophic lesions + Malignant-hyperthermia lesions<br>H: Dilated cardiomyopathy + inflammatory foci<br>Lu: Focal atelectasia<br>Li: Acute passive congestion	Malignant hyperthermia-like syndrome (linked to DMD pathology)
		1	0	D71	M: Dystrophic lesions + Malignant-hyperthermia lesions<br>H: Dilated cardiomyopathy + inflammatory foci<br>Lu: Avelolar emphysema<br>Li: Acute passive congestion	Malignant hyperthermia-like syndrome (linked to DMD pathology)
**DMD^{mdx} + 2.1x10^{14} vg/kg**	2 / 10	1	0	D60	M: Few centronucleated fibers<br>H: Right ventricular dilatation + blood vascular stasis + small inflammatory cell foci<br>Li: Acute passive congestion	Cardiac failure (linked to DMD pathology)
		0	1	D107	M: Early signs of MH<br>Lu: Alveolar emphysema (terminal dyspnea)<br>K: glomerulopathy-related lesions	Malignant hyperthermia-like syndrome (linked to DMD pathology)
**DMD^{mdx} + 4.2x10^{14} vg/kg**	4 / 10	0	1	D107	M: Dystrophic lesions + Malignant-hyperthermia lesions<br>Lu: Alveolar transudate	Malignant hyperthermia-like syndrome (linked to DMD pathology)
		0	1	D142	Mu: Very mild dystrophic lesions<br>Lu: Alveolar emphysema (terminal dyspnea)	No identified cause of death
		1	0	D157	M: Dystrophic lesions<br>H: Foci of inflammation and fibrosis<br>Lu: Alveolar emphysema (terminal dyspnea)	No identified cause of death
		0	1	D174	Mu: Very mild dystrophic lesions<br>Lu: Acute vascular congestion and alveolar edema<br>K: Hyaline casts, glomerulopathy-related lesions	Chronic progressive nephropathy
**TOTAL**	**9 / 31**	**5**	**4**			

*: cardiorespiratory arrest during handling before or after the telemetry session (i.e. installation or deinstallation of the telemetry probe + jacket, and placement in the telemetry cage) or animal found dead in its telemetry cage.
**: the following tissues were obtained and analyzed: biceps femoris muscle (BM) / heart (H) / lung (Lu) / liver (Li) / kidney (K) / spleen (S). Only observed lesions are reported in the table. If not noted, no abnormalities were detected in the other analyzed tissues.
***: Dystrophic lesions = isolated hyalin fibers, small clusters of degenerative fibers associated with muscle fiber regeneration foci, centro-nucleated fibers and anisocytosis with a few inflammatory cells in a slightly increased endomysial space corresponding to mild fibrosis. Malignant-hyperthermia lesions = large clusters of round hypereosinophilic fibers (hypercontracted fibers), some of them displaying fragmented cytoplasm and/or hyperchromatic condensed or fragmented nucleus indicative of necrosis (severe rhabdomyolysis) + huge optically empty space between fibers corresponding to massive edema.

<u>Table S3</u>: Proportion of animals presenting anti-hMD1 or anti-AAV8 cellular responses at 6 months after administration of vehicle or GNT0004 at the 4.2x10^{14} vg/kg dose. Analyses were performed on samples obtained from animals which survived to 6 months p.i. (number of positive rats / number of rats analyzed at sacrifice)

Experimental group	Number of rats positive for anti-hMD1 IgG antibodies	Number of rats positive for anti-hMD1 cellular response	Number of rats positive for anti-AAV8 cellular response
WT + Vehicle	0/10 (0%)	0/10 (0%)	0/10 (0%)
DMD^{mdx} + Vehicle	0/8 (0%)	0/8 (0%)	0/8 (0%)
**TOTAL (Vehicle-treated animals)**	**0/18 (0%)**	**0/18 (0%)**	**0/18 (0%)**
WT + 4.2x10^{14} vg/kg	1/6 (17%)	0/8 (0%)	5/8 (63%)
DMD^{mdx} + 4.2x10^{14} vg/kg	5/6 (83%)	0/6 (0%)	2/6 (33%)
**TOTAL (GNT0004-treated animals)**	**6/12 (50%)**	**0/14 (0%)**	**7/14 (50%)**

<u>Table S4</u>: Mean percentage of endogenous dystrophin-positive fibers *versus* hMD1-positive fibers in cardiac muscle.

Analyses were performed on samples obtained from animals which survived to 6 months p.i.

Data are presented as mean ± SEM

Experimental group	Number of animals	Mean percentage of endogenous dystrophin-positive fibers (NCL-DYS2 staining)	Mean percentage of hMD1-positive fibers (NCL-DYS3 staining)
**WT + Vehicle**	10	100% ± 0.0	0.0% ± 0.0
**WT + 2.1x10^{14} vg/kg**	7	99.1% ± 1.1	93.2% ± 3.9
**WT + 4.2x10^{14} vg/kg**	7	98.2% ± 3.1	94.6% ± 14.2
**DMD^{mdx} + Vehicle**	8	3.6% ± 4.5	0.0% ± 0
**DMD^{mdx} + 2.1x10^{14} vg/kg**	8	0.8% ± 1.6	96.6% ± 2.7
**DMD^{mdx} + 4.2x10^{14} vg/kg**	6	0.0% ± 0.0	100% ± 0.0

A

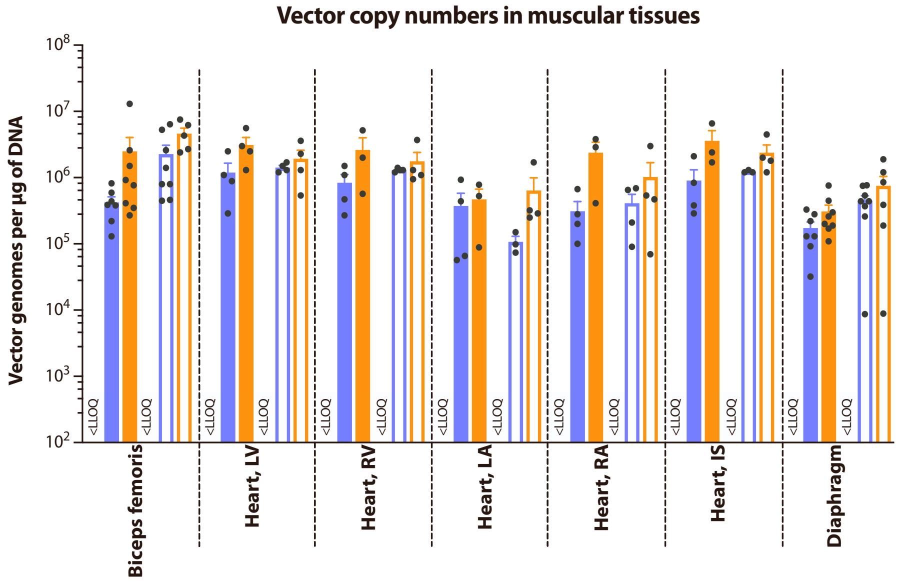

B

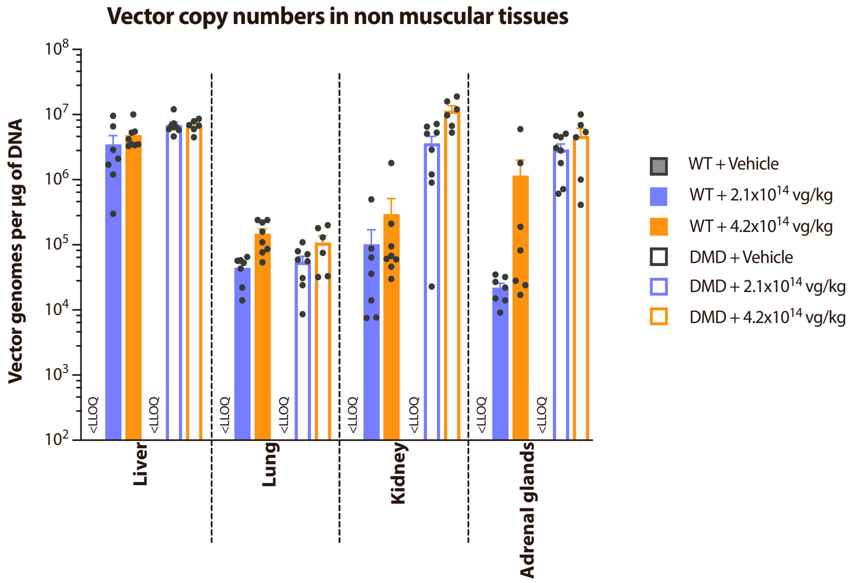

<u>Figure S1</u>: GNT0004 biodistribution (vector genome copies per µg of DNA) in tissue samples from WT and DMD*mdx* rats sacrificed at 6 months post-injection.

Analyses were performed on samples obtained from animals which survived to 6 months p.i. (i.e. n=3 to 5 per experimental group for the heart samples and n=6 to 10 per experimental group for the other tissues). (A) Vector genome copies per µg of DNA in muscle tissues, (B) Vector genome copies per µg of DNA in non-muscle tissues. Vector copy numbers per diploid genomes were determined using qPCR. Results are expressed as the mean ± SEM.

Statistical analyses, performed using nonparametric Kruskal-Wallis test followed by a post hoc Dunn's multiple comparisons test, showed no significant differences between the different groups injected with GNT0004. LLOQ = Lower Limit of Quantification = 50 vector genomes per µg of genomic DNA.

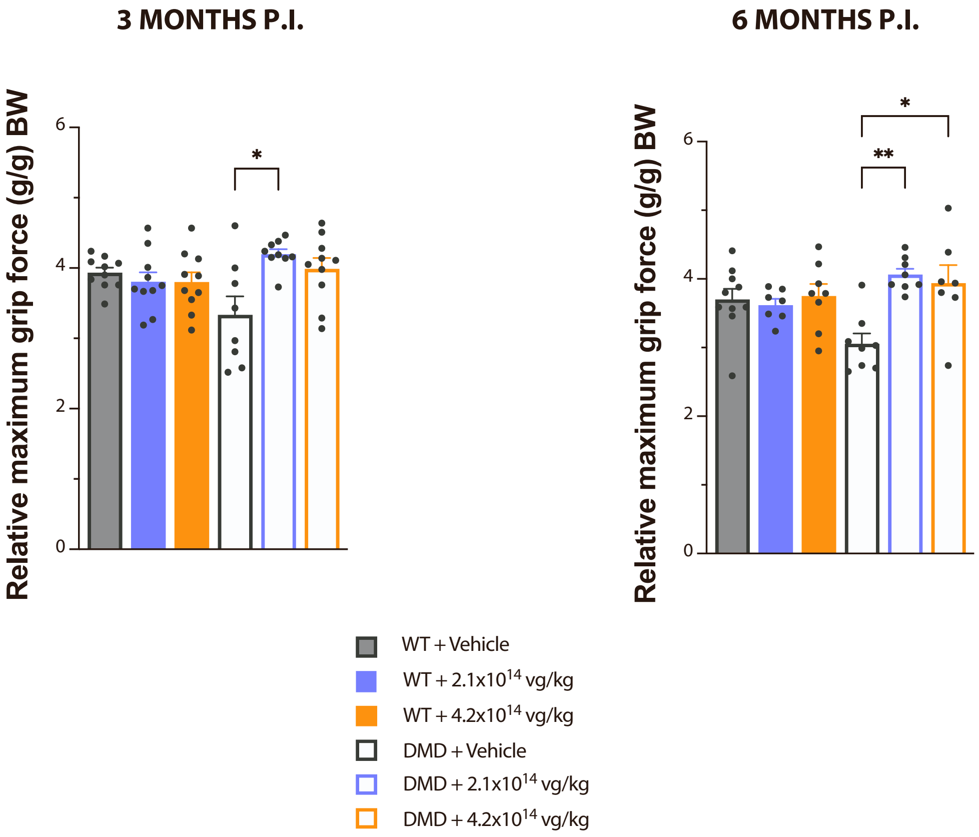

<u>Figure S2</u>: Maximum forelimb grip forces measured at 3 and 6 months after vehicle or GNT0004 administration.

Analyses were performed on animals which survived to the time of analysis (i.e. n=7 to 10 per experimental group).The highest grip force value obtained over five successive grip test trials was considered as the maximum forelimb grip force and was plotted as relative force (in g/g normalized to body weight [BW]). Data are shown as mean values ± SEM. Statistical analyses were performed using nonparametric Kruskal-Wallis test followed by a post hoc Dunn's multiple comparisons test (**p<0.01; *p<0.05).

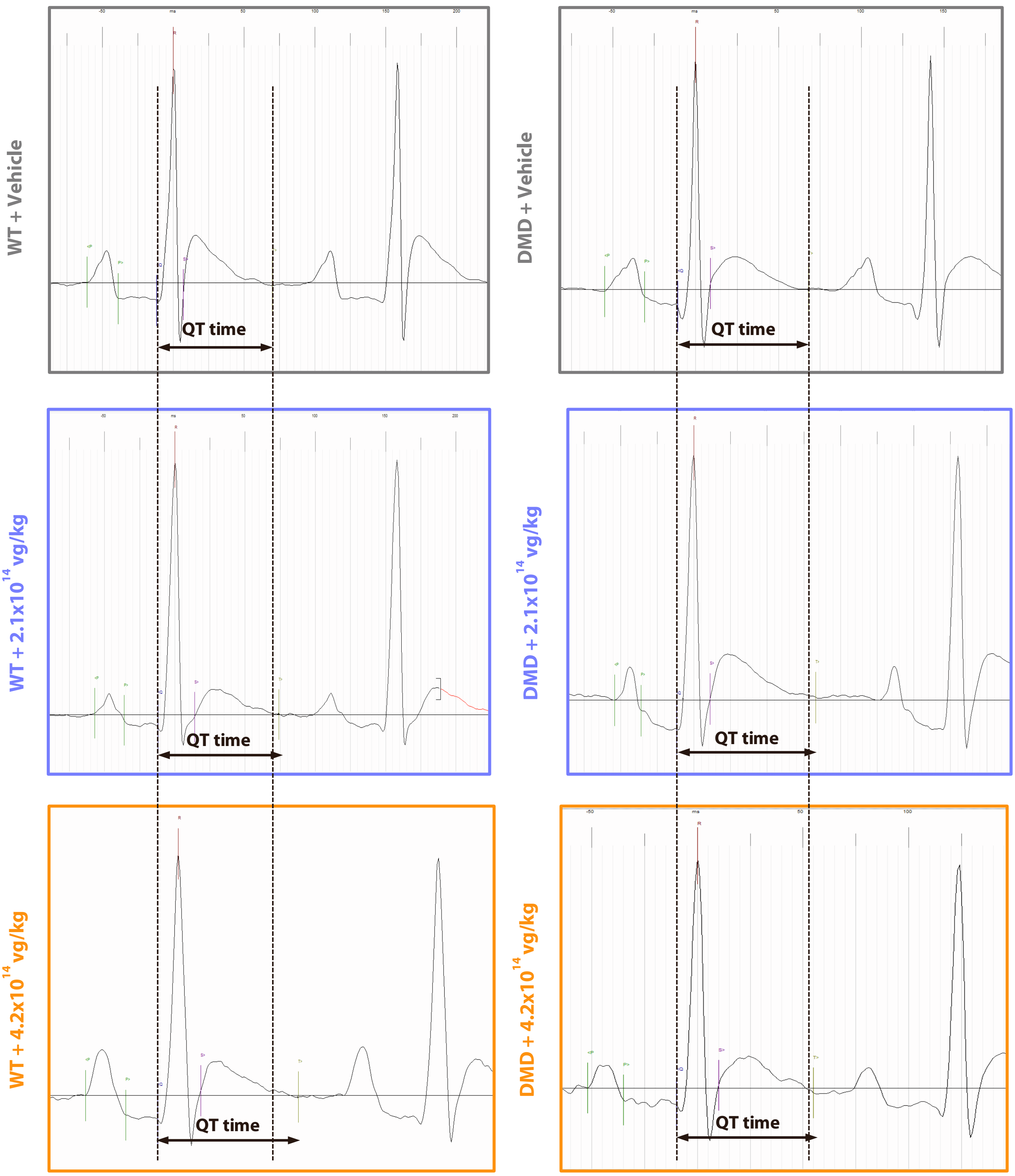

Figure S3: Assessment of hMD1 expression on electrocardiographic (ECG) parameters at 6 months after vehicle or GNT0004 administration. Representative ECG traces from each experimental group. QT intervals measurements are shown, with the dashed lines indicating the QT interval measured in each vehicle-injected control group.

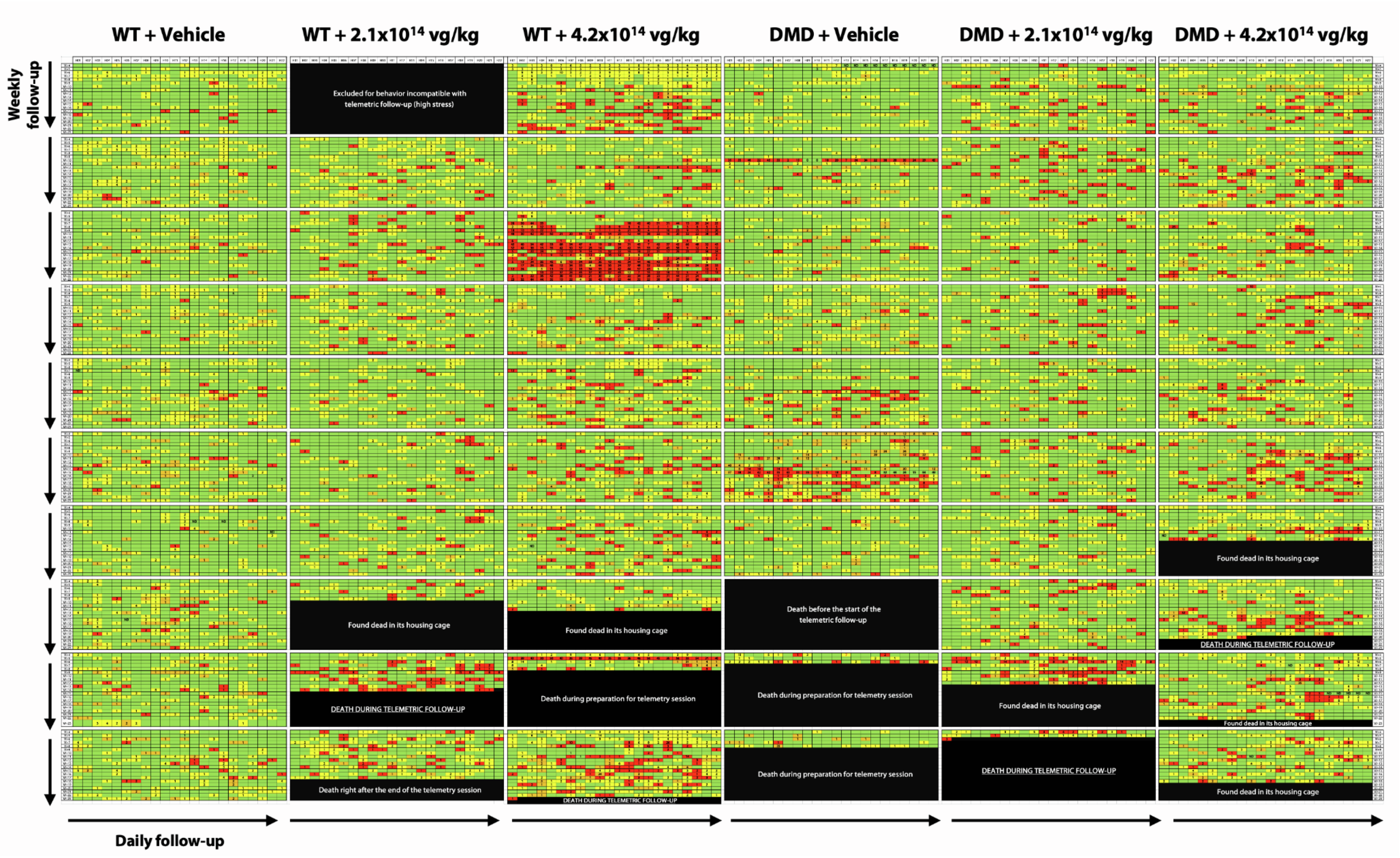

WT + Vehicle
WT + 2.1x10^{14} vg/kg
WT + 4.2x10^{14} vg/kg
DMD + Vehicle
DMD + 2.1x10^{14} vg/kg
DMD + 4.2x10^{14} vg/kg
Weekly follow-up
Excluded for behavior incompatible with telemetric follow-up (high stress)
Found dead in its housing cage
Found dead in its housing cage
Death before the start of the telemetric follow-up
Found dead in its housing cage
DEATH DURING TELEMETRIC FOLLOW-UP
DEATH DURING TELEMETRIC FOLLOW-UP
Death during preparation for telemetry session
Death during preparation for telemetry session
Found dead in its housing cage
Found dead in its housing cage
Death right after the end of the telemetry session
DEATH DURING TELEMETRIC FOLLOW-UP
Death during preparation for telemetry session
DEATH DURING TELEMETRIC FOLLOW-UP
Found dead in its housing cage
Daily follow-up

Figure S4 (previous page): Schematic representation of ECG results obtained using telemetry in the different groups of animals.

A color coding system was used to represent the rhythm disorders for each hour of ECG recorded during 20 sessions of 22 hours (daily follow-up) conducted every week during the course of the study (weekly follow-up). The events observed during each one-hour period were classified into three categories: events with low (yellow), medium (orange), or high (red) potential impact on cardiac electrical function. Absence of events are represented in green. The number of events observed each hour is indicated in the corresponding box. Black boxes denote animals that died prematurely during the study.

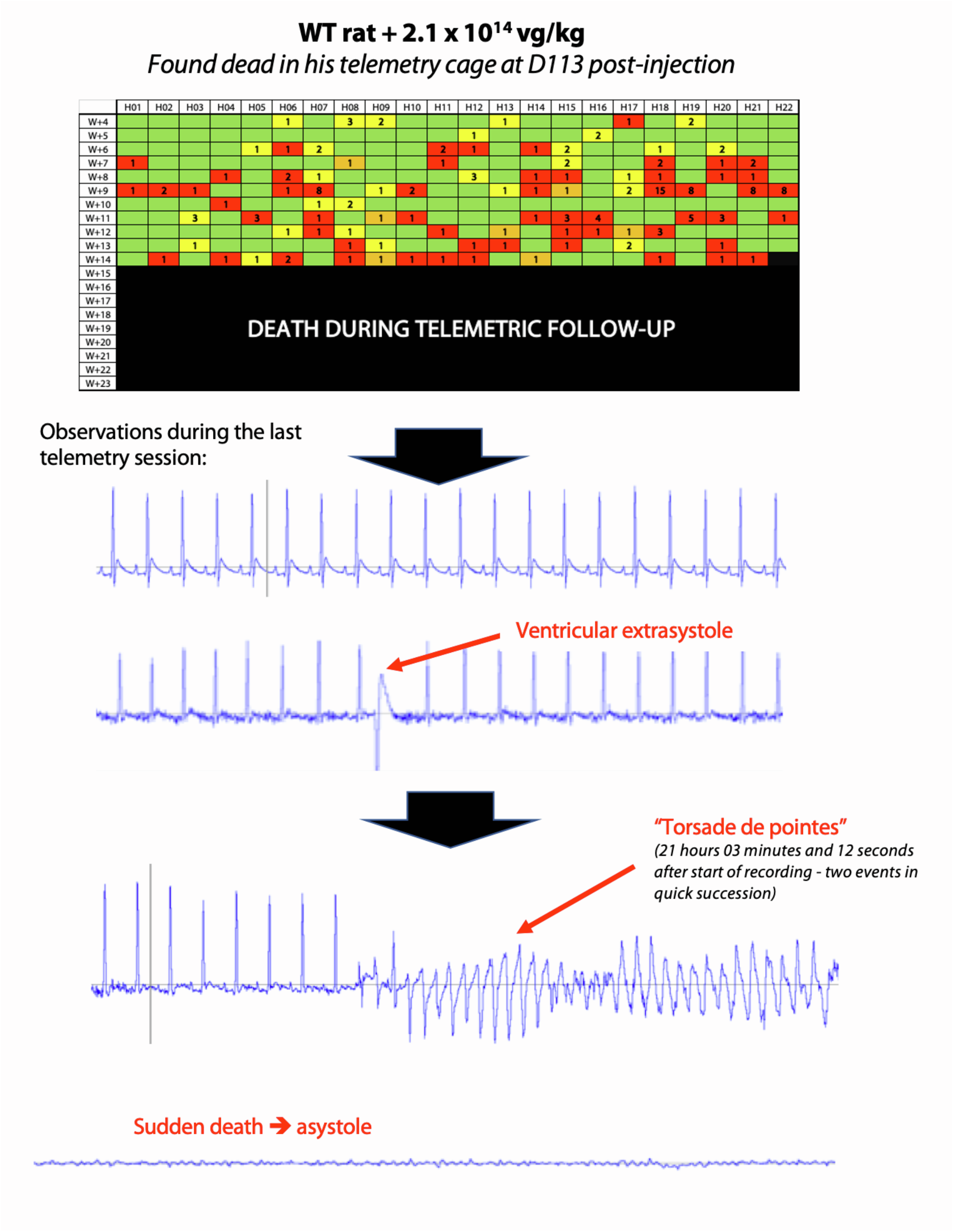

	H01	H02	H03	H04	H05	H06	H07	H08	H09	H10	H11	H12	H13	H14	H15	H16	H17	H18	H19	H20	H21	H22
W+4						1		3	2				1				1		2			
W+5												1				2						
W+6					1	1	2				2	1		1	2			1		2		
W+7	1							1			1				2			2		1	2	
W+8				1		2	1					3		1	1		1	1		1	1	
W+9	1	2	1			1	8		1	2			1	1	1		2	15	8		8	8
W+10				1			1	2														
W+11			3		3		1		1	1				1	3	4			5	3		1
W+12						1	1	1			1		1		1	1	1	3				
W+13			1					1	1			1	1		1		2			1		
W+14		1		1	1	2		1	1	1	1	1		1				1		1	1	
W+15	DEATH DURING TELEMETRIC FOLLOW-UP																					
W+16																						
W+17																						
W+18																						
W+19																						
W+20																						
W+21																						
W+22																						
W+23																						

<u>Figure S5</u>: Schematic representation of ECG telemetry results in a rat from the WT + 2.1x10^{14} vg/kg group that died prematurely during a telemetry session (during the 21st hour of the 11th session).

Representative ECG traces obtained during this recording session are presented. One ventricular extrasystole was observed during this session. Major rhythm disorders were observed prior to "torsade de pointes", leading to sudden death (asystole) of the animal the following minute.

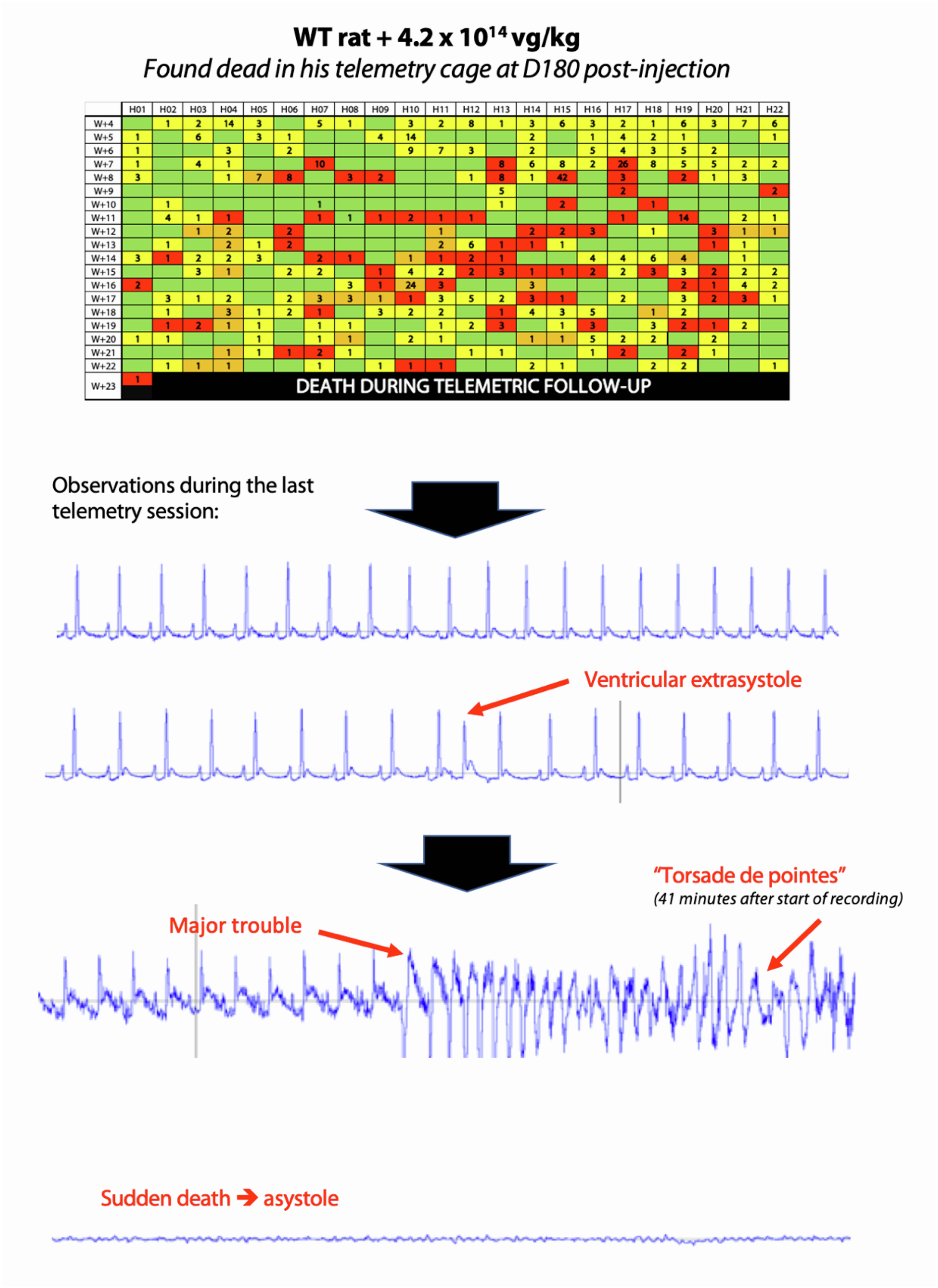

	H01	H02	H03	H04	H05	H06	H07	H08	H09	H10	H11	H12	H13	H14	H15	H16	H17	H18	H19	H20	H21	H22
W+4		1	2	14	3		5	1		3	2	8	1	3	6	3	2	1	6	3	7	6
W+5	1		6		3	1			4	14				2		1	4	2	1			1
W+6	1			3		2				9	7	3		2		5	4	3	5	2		
W+7	1		4	1			10						8	6	8	2	26	8	5	5	2	2
W+8	3			1	7	8		3	2			1	8	1	42		3		2	1	3	
W+9													5				2					2
W+10		1					1						1		2			1				
W+11		4	1	1			1	1	1	2	1	1					1		14		2	1
W+12			1	2		2					1			2	2	3		1		3	1	1
W+13		1		2	1	2					2	6	1	1	1					1	1	
W+14	3	1	2	2	3		2	1		1	1	2	1			4	4	6	4		1	
W+15			3	1		2	2		1	4	2	2	3	1	1	2	2	3	3	2	2	2
W+16	2							3	1	24	3			3					2	1	4	2
W+17		3	1	2		2	3	3	1	1	3	5	2	3	1		2		3	2	3	1
W+18		1		3	1	2	1		3	2	2		1	4	3	5		1	2			
W+19		1	2	1	1		1	1			1	2	3		1	3		3	2	1	2	
W+20	1	1			1		1	1		2	1			1	1	5	2	2		2		
W+21				1	1	1	2	1				1	1			1	2		2	1		
W+22		1	1	1			1		1	1	1			2	1			2	2			1
W+23	1	DEATH DURING TELEMETRIC FOLLOW-UP																				

<u>Figure S6</u>: Schematic representation of ECG telemetry results in a rat from the WT + 4.2x10^{14} vg/kg group that died prematurely during a telemetry session (during the 1st hour of the 20th session).

Representative ECG traces obtained during this recording session are presented. One ventricular extrasystole was observed during this session. Major rhythm disorders were observed prior to "torsade de pointes", leading to sudden death (asystole) of the animal the following minute.

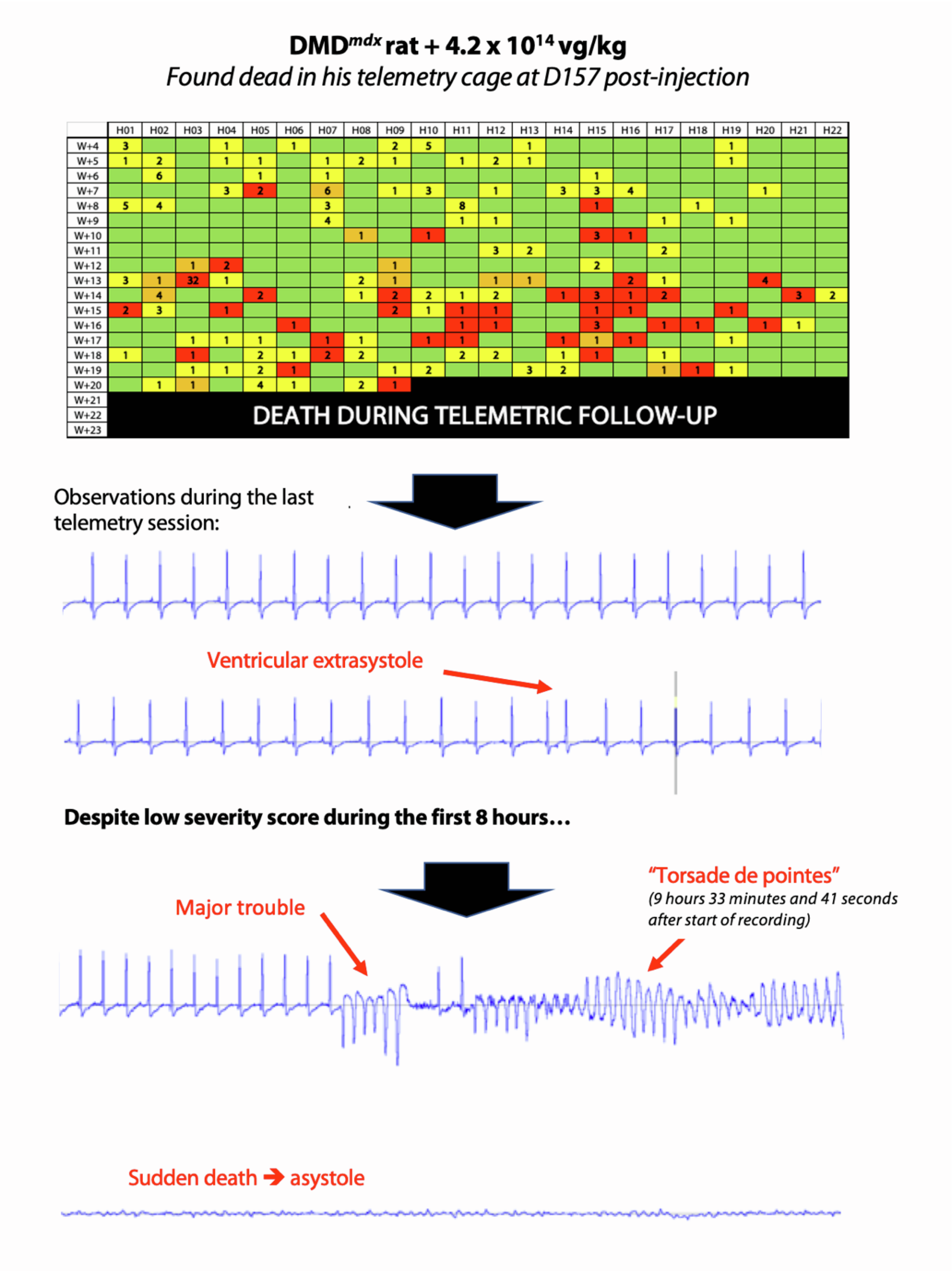

<u>Figure S7</u>: Schematic representation of ECG telemetry results in a rat from the DMD^{mdx} + 4.2x10^{14} vg/kg group that died prematurely during a telemetry session (during the 9th hour of the 17th session).

Representative ECG traces obtained during this recording session are presented. One ventricular extrasystole was observed during this session. Major rhythm disorders were observed prior to "torsade de pointes", leading to sudden death (asystole) of the animal the following minute.

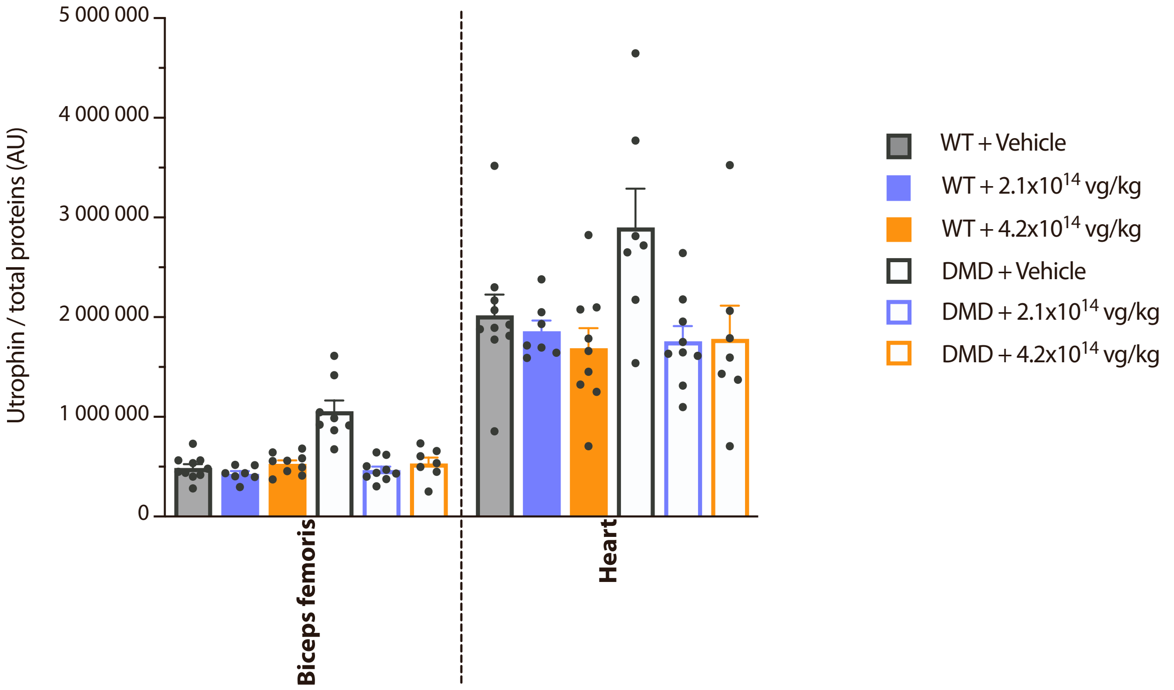

<u>Figure S8</u>: Levels of utrophin protein expression in muscles from WT and DMD^{mdx} rats 6 months after administration of vehicle or GNT0004.

Analyses were performed on samples obtained from animals at 6 months p.i. (i.e. n=6 to 10 per experimental group). Quantification of utrophin protein expression by Simple Western from total proteins extracted from biceps femoris and heart. Quantification was done respective to the level of total proteins and expressed as arbitrary units (AU). Individual and mean values (± SEM) are shown. Statistical analyses, performed using nonparametric Kruskal-Wallis test followed by a post hoc Dunn's multiple comparisons test, showed no significant differences between the different groups injected with GNT0004.

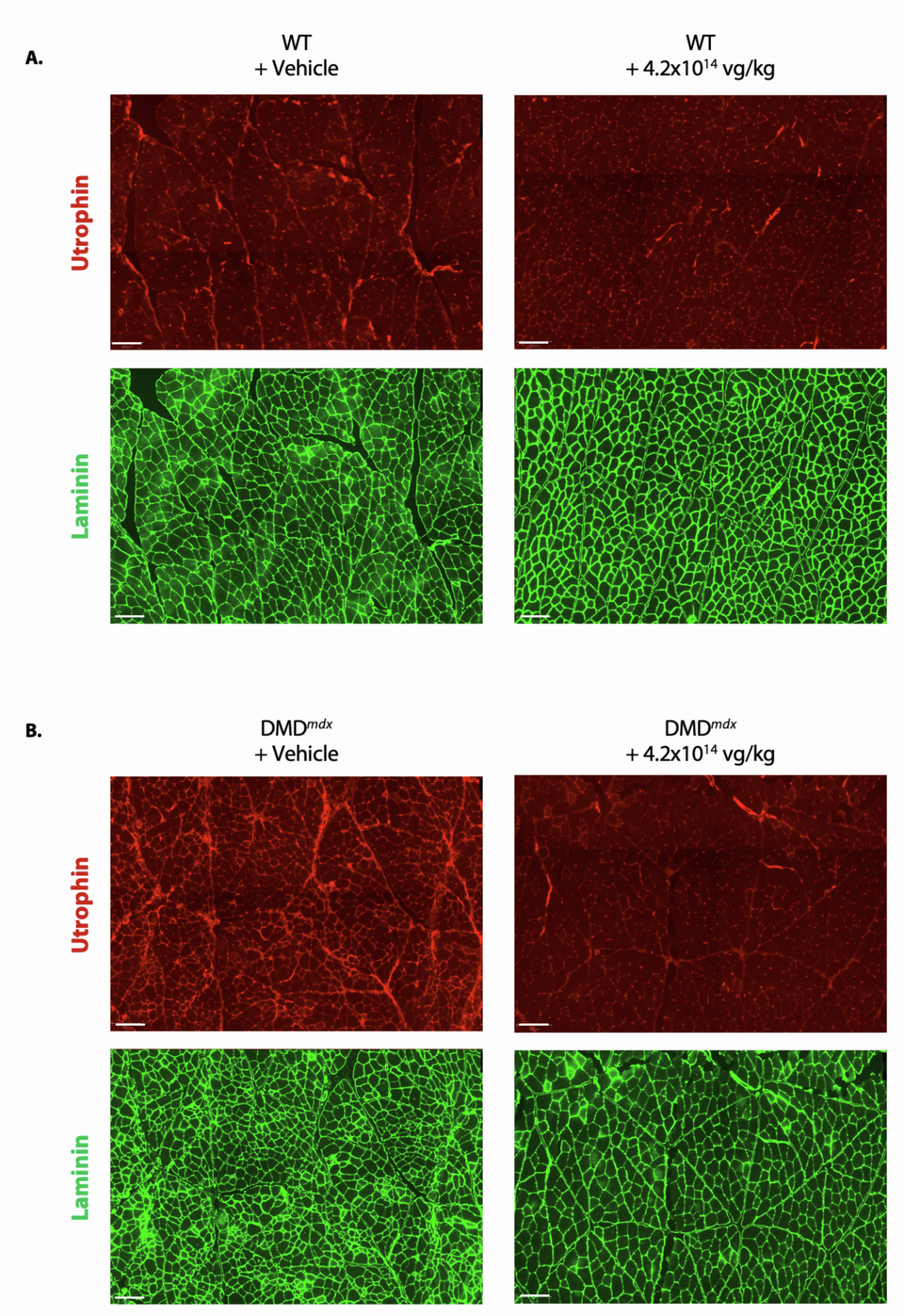

<u>Figure S9</u>: Utrophin protein immunostaining in *biceps femoris* muscle of WT and DMD^{mdx} rats 6 months after administration of vehicle or GNT0004.
Immunohistochemical analysis of utrophin expression in the *biceps femoris* of injected WT rats (A) and injected DMD^{mdx} rats (B). Representative results are presented for one animal of each experimental group. Laminin immunostaining was used to identify the basal membrane. Scale bar = 200µm.

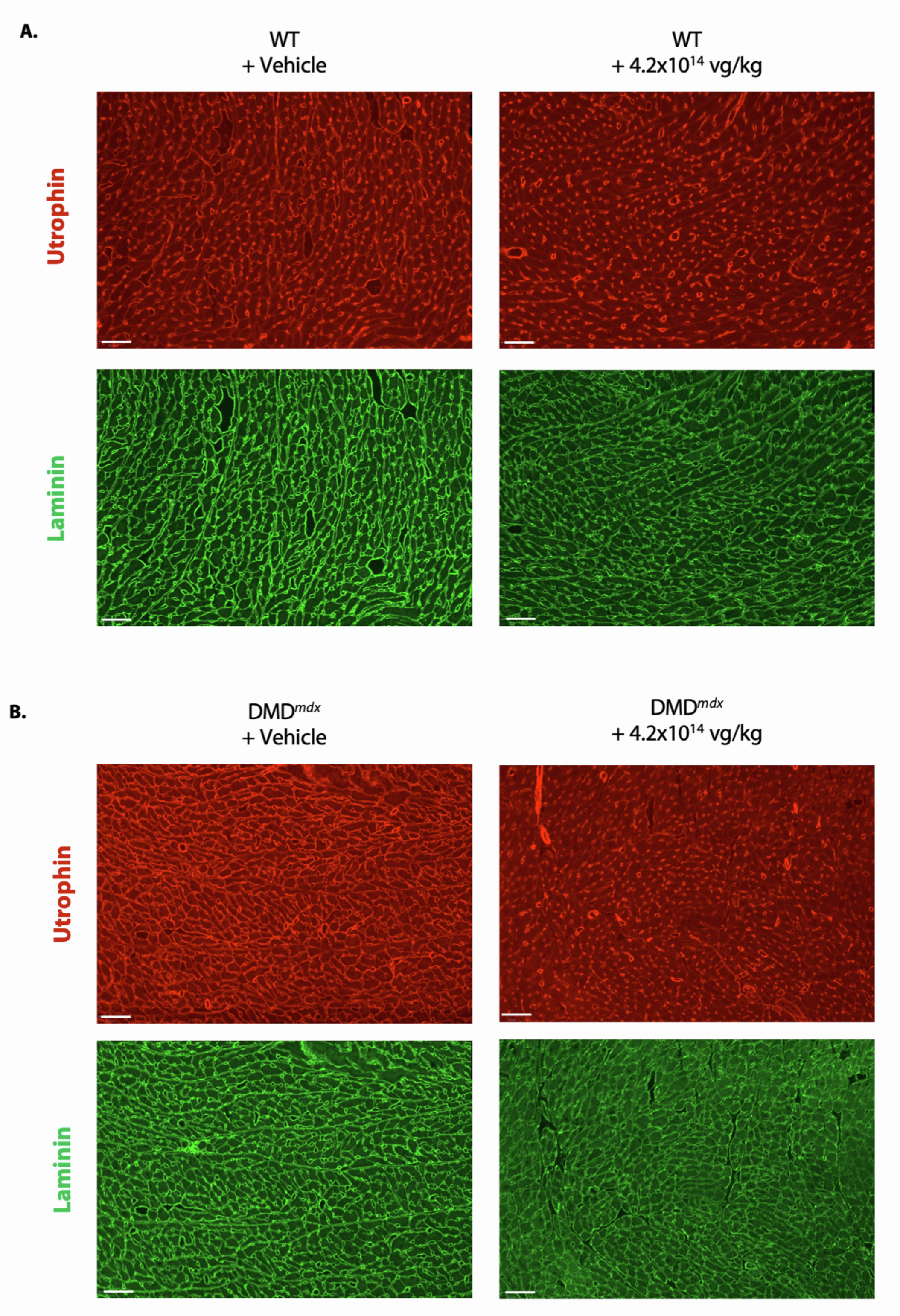

Figure S10: Utrophin protein immunostaining in cardiac muscle of WT and DMD^{mdx} rats 6 months after administration of vehicle or GNT0004.

Immunohistochemical analysis of utrophin expression in the hearts of injected WT rats (A) and injected DMD^{mdx} rats (B). Representative results are presented for one animal of each experimental group. Laminin immunostaining was used to identify the basal membrane. Scale bar = 200µm.
